# Supplementary material for: Acute Pancreatitis—Drivers of Hospitalisation Cost—A Seven-Year Retrospective Study from a Large Tertiary Center
Source: Healthcare (Basel). 2023 Sep 7;11(18):2482. doi: 10.3390/healthcare11182482 (PMC10531218; doi:10.3390/healthcare11182482)
Supplement: Supplementary file 1 [file healthcare-11-02482-s001.zip › healthcare-2571337-supplementary.pdf]

Supplementary Materials :

All processed statistical data of paper: ACUTE PANCREATITIS – DRIVERS OF HOSPITALISATION COST– A SEVEN-YEAR RETROSPECTIVE STUDY FROM A LARGE TERTIARY CENTER

Case Processing Summary

	Cases					
	Valid		Missing		Total	
	N	Percent	N	Percent	N	Percent
Cost/day	285	100.0%	0	0.0%	285	100.0%

Extreme Values

			Case Number	Value
Cost/day	Highest	1	1319	2137.000000000000000
		2	341	1733.627647000000000
		3	807	1537.666666666666700
		4	713	1517.303333333333300
		5	1015	1504.383333333333200
	Lowest	1	492	250.358000000000000
		2	206	252.000000000000000
		3	257	277.444444444444460
		4	88	344.142857142857170
		5	6	440.285714285714300

Tests of Normality

	Kolmogorov-Smirnov[a]			Shapiro-Wilk		
	Statistic	df	Sig.	Statistic	df	Sig.
Cost/day	.139	285	<.001	.897	285	<.001

a. Lilliefors Significance Correction

Cost/day

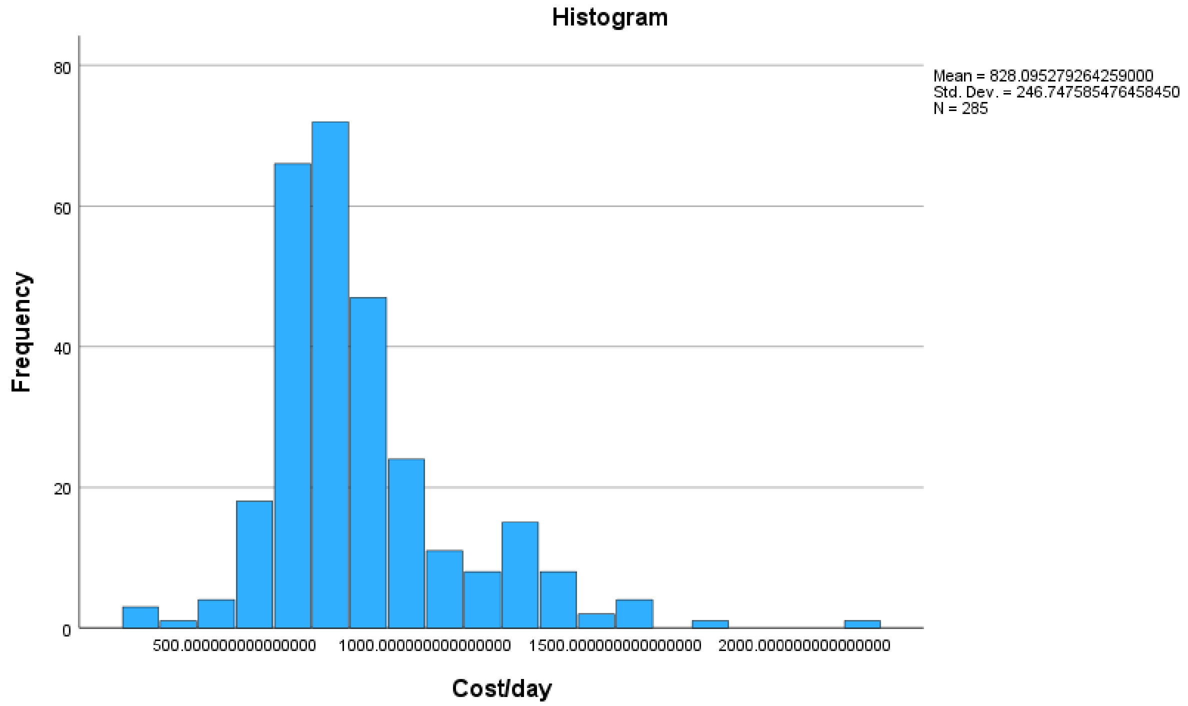


```
Cost/day Stem-and-Leaf Plot

 Frequency    Stem &  Leaf

     3.00 Extremes    (=<277)
     1.00        3 .  4
      .00        3 .
     2.00        4 .  44
     2.00        4 .  69
```

```
    5.00        5 .  00223
   13.00        5 .  5666789999999
   27.00        6 .  000001111122223333444444444
   39.00        6 .  555555556666666777777777778888899999999
   36.00        7 .  000000001111111222222233333334444444
   36.00        7 .  555555556666666666777777778888889999
   26.00        8 .  00011111122222333333444444
   21.00        8 .  555555677777778888999
    6.00        9 .  000014
   18.00        9 .  555566667888888899
    7.00       10 .  0112344
    4.00       10 .  5678
    7.00       11 .  0112334
    1.00       11 .  6
    9.00       12 .  000112334
    1.00       12 .  5
   21.00 Extremes    (>=1278)

Stem width:  100.0000
Each leaf:        1 case(s)
```

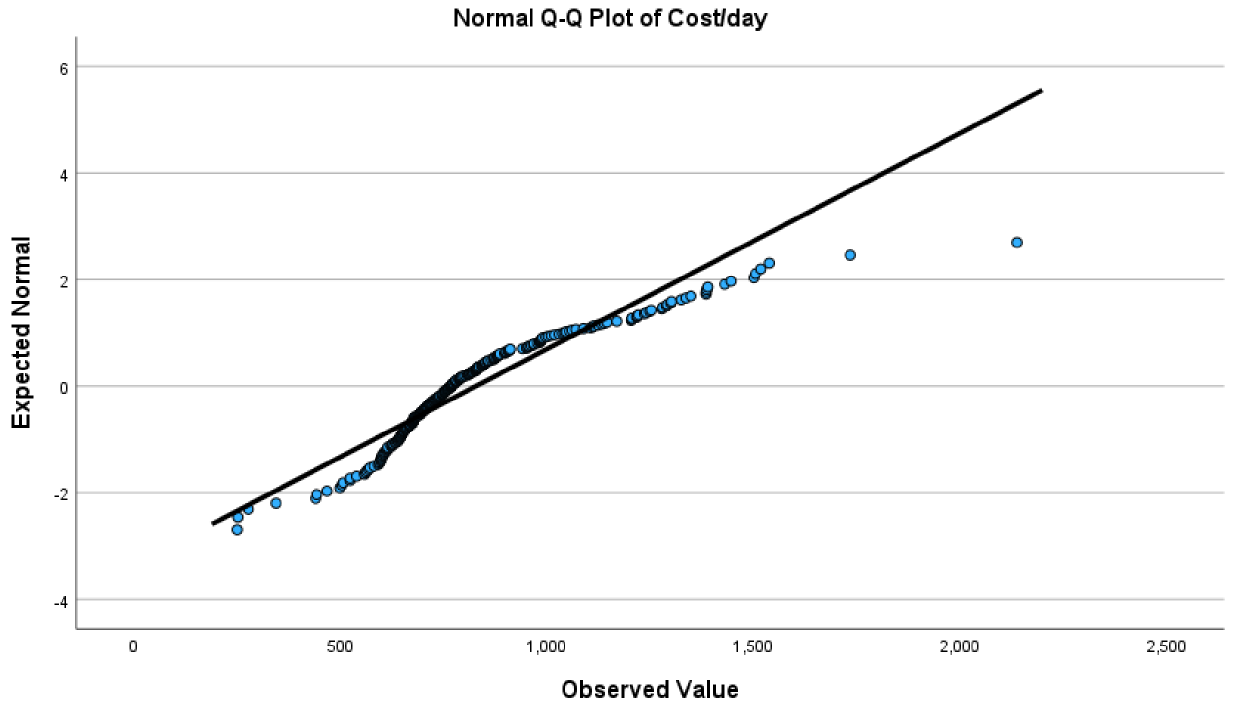

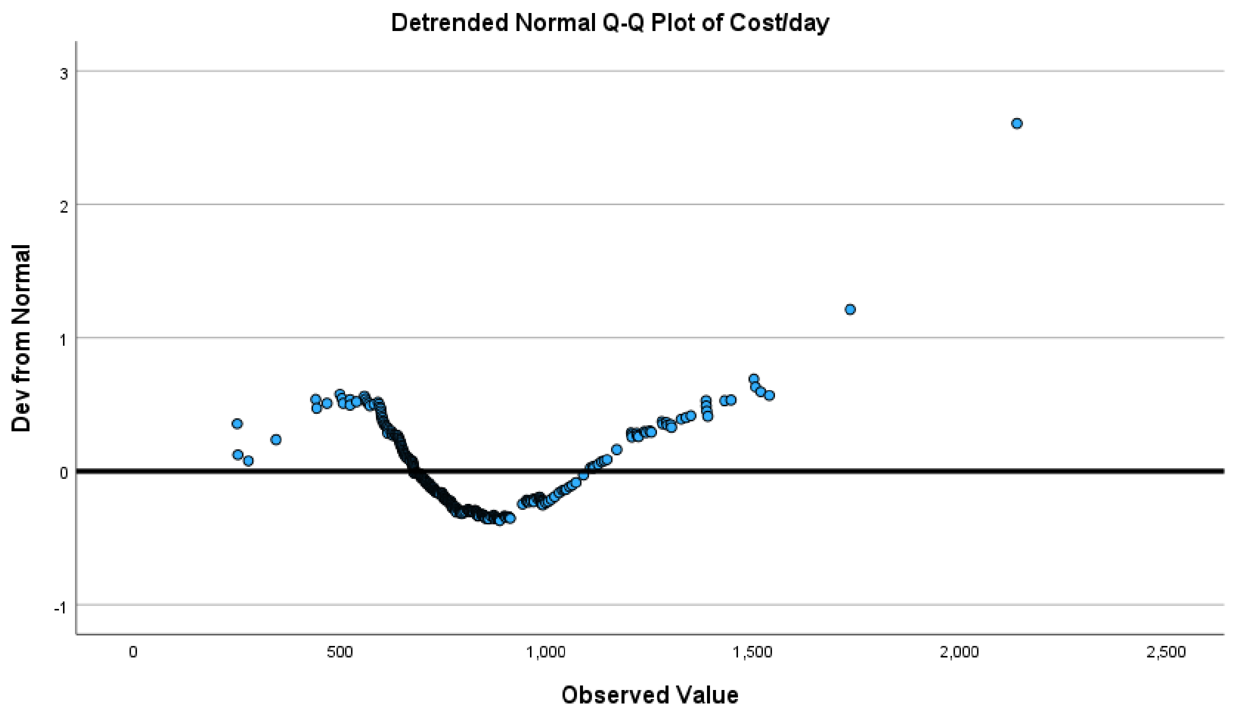
Detrended Normal Q-Q Plot of Cost/day
Dev from Normal
Observed Value
3
2
1
0
-1
0
500
1,000
1,500
2,000
2,500

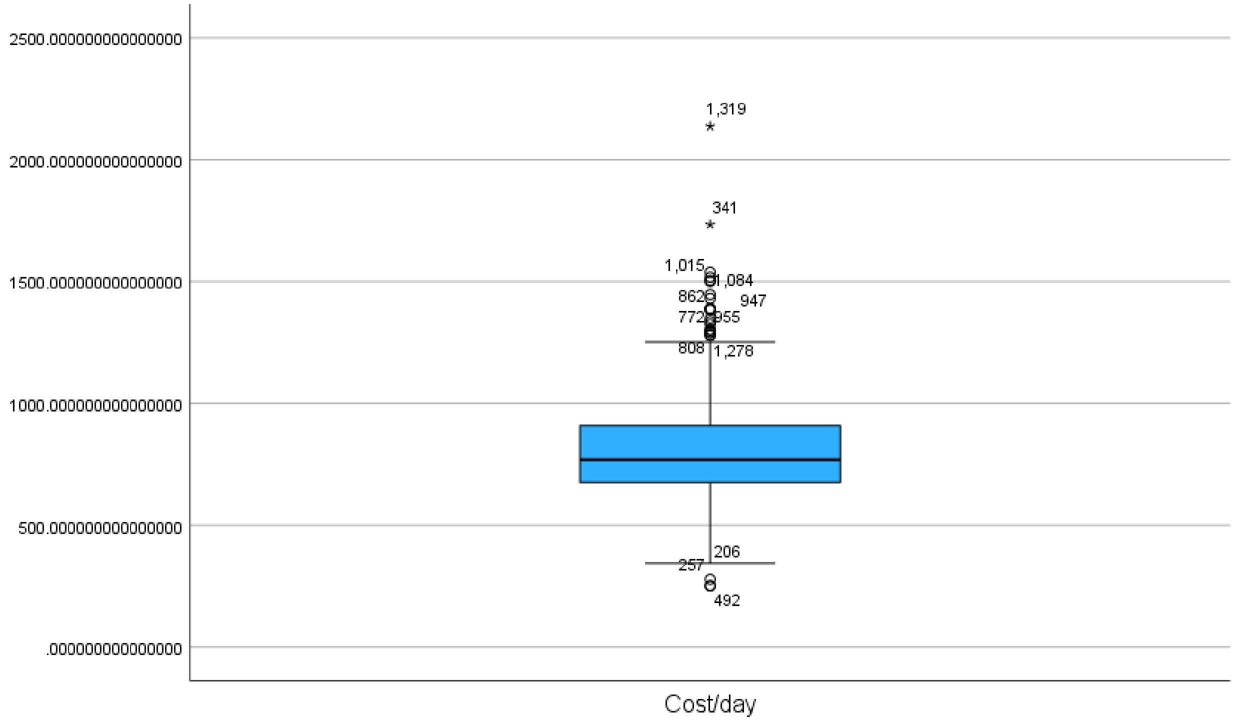
2500.000000000000000
2000.000000000000000
1500.000000000000000
1000.000000000000000
500.000000000000000
.000000000000000
1,319
341
1,015
1,084
862
947
772
955
808
1,278
206
257
492
Cost/day

Explore FORMER SMOKER

Notes

Output Created		07-MAY-2023 16:19:23
Comments		
Input	Data	C:\Users\paho9\OneDrive\Documente\Doctorat - Stratificarea severității și predicția prognosticului în faza incipientă a Pancreatitei Acute\Registru Pancreatite Acute - BUC-API\Baza date nou\Articole\Articol cost\DB_corect_COST.sav
	Active Dataset	DataSet1
	Filter	Smoking = 2 (FILTER)
	Weight	<none>
	Split File	<none>
	N of Rows in Working Data File	85
Missing Value Handling	Definition of Missing	User-defined missing values for dependent variables are treated as missing.
	Cases Used	Statistics are based on cases with no missing values for any dependent variable or factor used.

Syntax		EXAMINE VARIABLES=Costday<br>/PLOT BOXPLOT STEMLEAF HISTOGRAM NPPLOT<br>/COMPARE GROUPS<br>/STATISTICS DESCRIPTIVES EXTREME<br>/CINTERVAL 95<br>/MISSING LISTWISE<br>/NOTOTAL.
Resources	Processor Time	00:00:00.67
	Elapsed Time	00:00:00.69

Case Processing Summary

	Cases					
	Valid		Missing		Total	
	N	Percent	N	Percent	N	Percent
Cost/day	85	100.0%	0	0.0%	85	100.0%

Descriptives

			Statistic	Std. Error
Cost/day	Mean		819.398968621477900	29.668361479704053
	95% Confidence Interval for Mean	Lower Bound	760.400178179620500	
		Upper Bound	878.397759063335300	
	5% Trimmed Mean		793.169972137776500	
	Median		802.571428571428600	
	Variance		74817.992	
	Std. Deviation		273.528777637167300	

	Minimum	534.000000000000000	
	Maximum	2942.240000000000000	
	Range	2408.240000000000000	
	Interquartile Range	195.556111111111020	
	Skewness	5.674	.261
	Kurtosis	43.520	.517

Extreme Values

			Case Number	Value
Cost/day	Highest	1	602	2942.240000000000000
		2	762	1260.497500000000000
		3	248	1075.428571428571300
		4	1263	1074.387500000000000
		5	1467	1046.786000000000000
	Lowest	1	13	534.000000000000000
		2	622	548.857142857142900
		3	271	563.700000000000000
		4	1457	565.016000000000000
		5	465	582.345000000000000

Tests of Normality

Kolmogorov-Smirnov[a]	Shapiro-Wilk

	Statistic	df	Sig.	Statistic	df	Sig.
Cost/day	.188	85	<.001	.549	85	<.001

a. Lilliefors Significance Correction

Cost/day

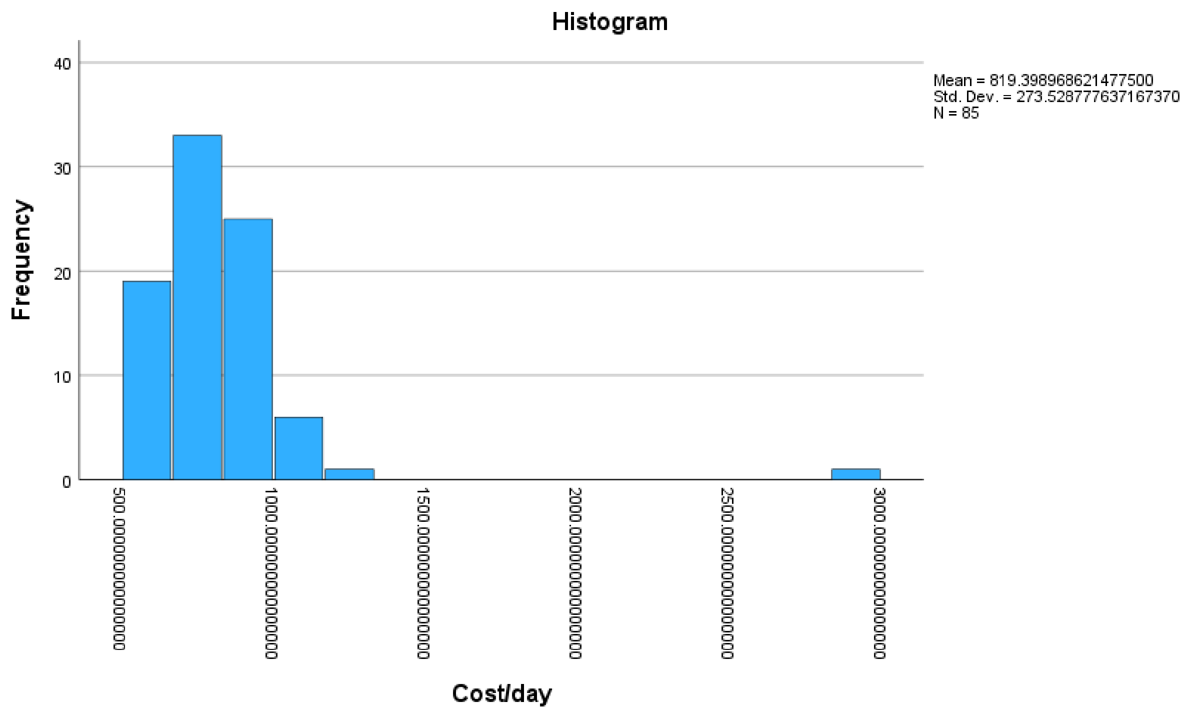


```
Cost/day Stem-and-Leaf Plot

 Frequency    Stem &  Leaf

     9.00        5 .  346688999
    15.00        6 .  012233444567899
    17.00        7 .  01124444557778899
    25.00        8 .  0001111122244445555667788
    11.00        9 .  02334556678
     6.00       10 .  012477
     2.00 Extremes    (>=1260)
```

```
Stem width:  100.0000
Each leaf:        1 case(s)
```

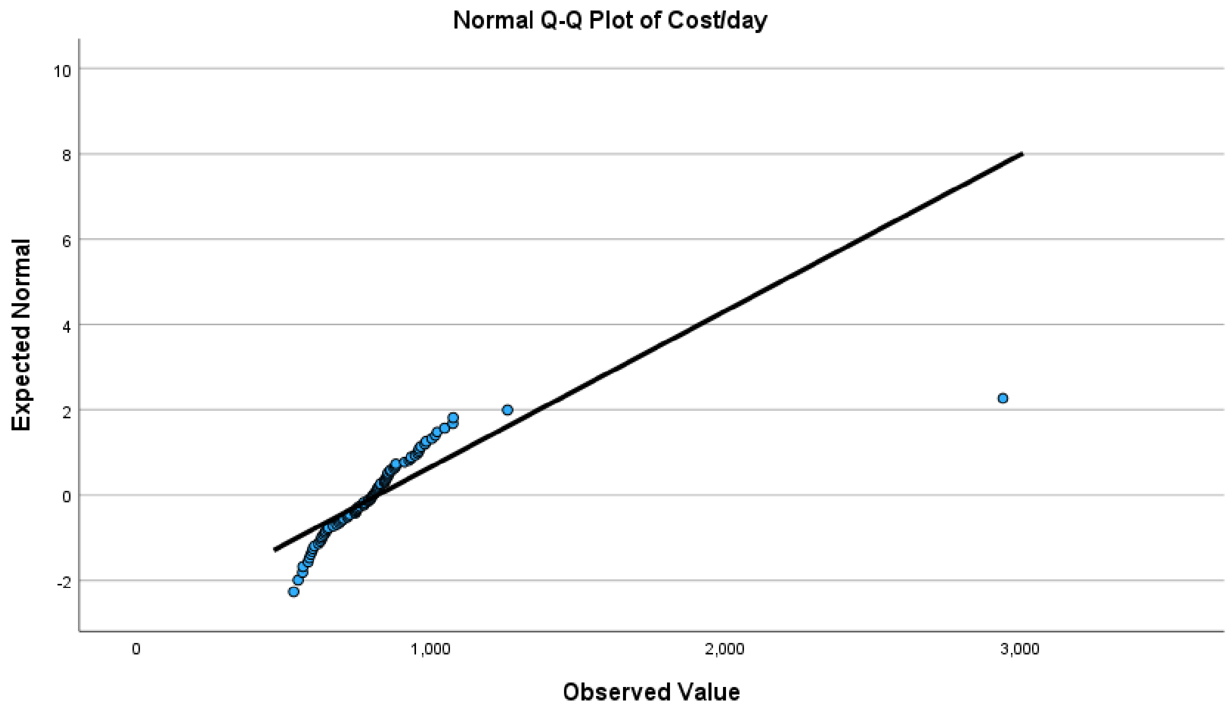

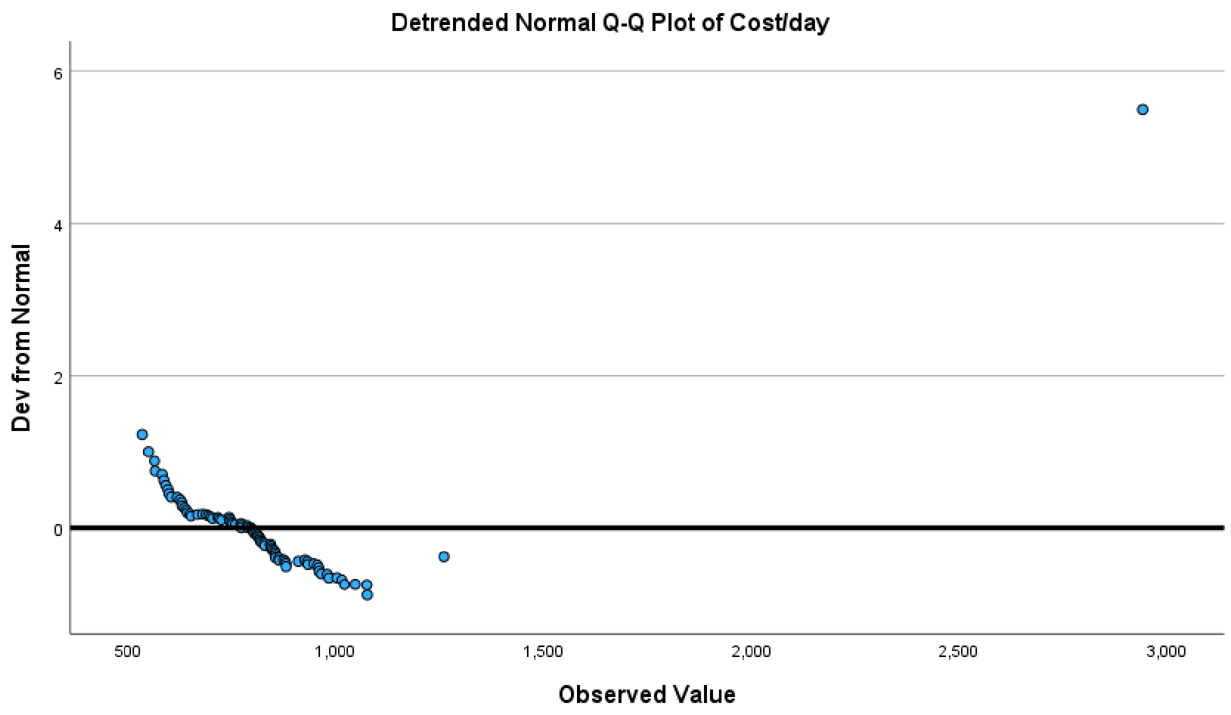

Detrended Normal Q-Q Plot of Cost/day
Dev from Normal
6
4
2
0
500
1,000
1,500
2,000
2,500
3,000
Observed Value

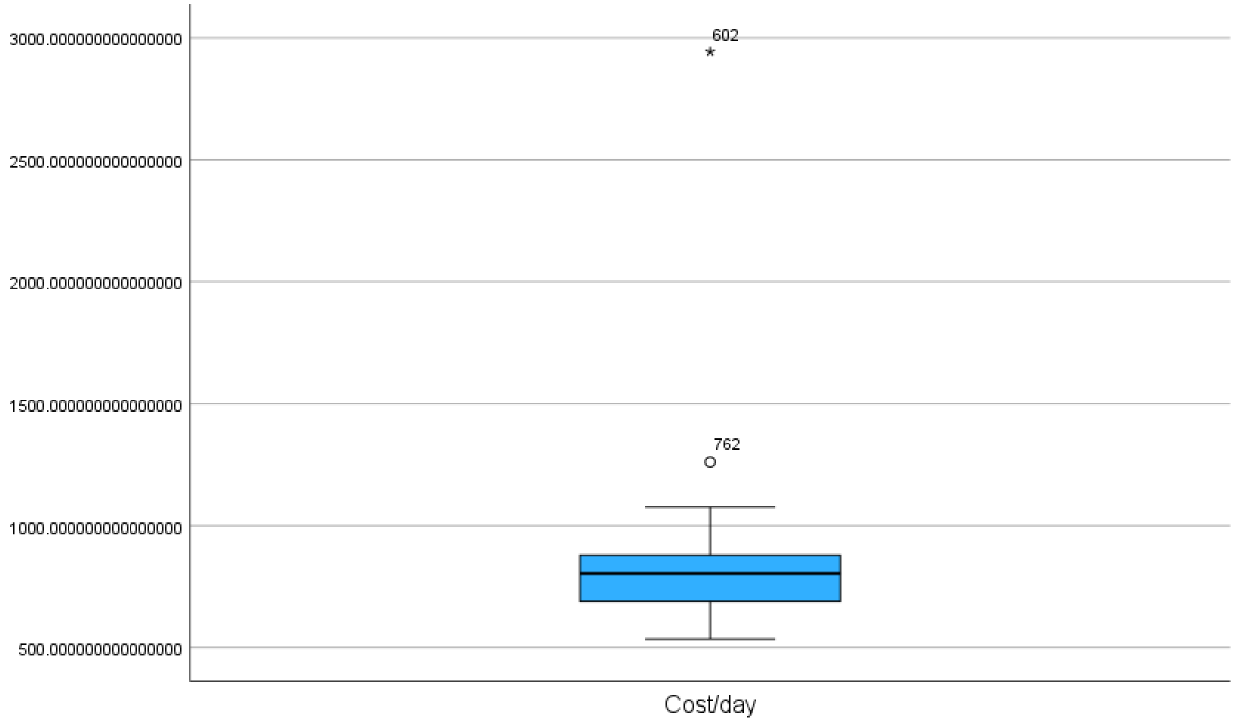

3000.000000000000000
2500.000000000000000
2000.000000000000000
1500.000000000000000
1000.000000000000000
500.000000000000000
602
762
Cost/day

Explore NOT SMOKING

Notes

Output Created		07-MAY-2023 16:20:12
Comments		
Input	Data	C:\Users\paho9\OneDrive\Documente\Doctorat - Stratificarea severității și predicția prognosticului în faza incipientă a Pancreatitei Acute\Registru Pancreatite Acute - BUC-API\Baza date nou\Articole\Articol cost\DB_corect_COST.sav
	Active Dataset	DataSet1
	Filter	Smoking = 3 (FILTER)
	Weight	<none>
	Split File	<none>
	N of Rows in Working Data File	29
Missing Value Handling	Definition of Missing	User-defined missing values for dependent variables are treated as missing.
	Cases Used	Statistics are based on cases with no missing values for any dependent variable or factor used.

Syntax		EXAMINE VARIABLES=Costday<br>/PLOT BOXPLOT STEMLEAF HISTOGRAM NPPLOT<br>/COMPARE GROUPS<br>/STATISTICS DESCRIPTIVES EXTREME<br>/CINTERVAL 95<br>/MISSING LISTWISE<br>/NOTOTAL.
Resources	Processor Time	00:00:00.58
	Elapsed Time	00:00:00.68

Case Processing Summary

	Cases					
	Valid		Missing		Total	
	N	Percent	N	Percent	N	Percent
Cost/day	29	100.0%	0	0.0%	29	100.0%

Descriptives

			Statistic	Std. Error
Cost/day	Mean		907.858538915107800	80.448761515688990
	95% Confidence Interval for Mean	Lower Bound	743.066721277803000	
		Upper Bound	1072.650356552412600	
	5% Trimmed Mean		842.067108099435700	
	Median		854.021999999999800	
	Variance		187688.094	
	Std. Deviation		433.229839291845000	

	Minimum	542.942857142857200	
	Maximum	2998.000000000000000	
	Range	2455.057142857143000	
	Interquartile Range	240.085833333333430	
	Skewness	4.229	.434
	Kurtosis	20.732	.845

Extreme Values

			Case Number	Value
Cost/day	Highest	1	131	2998.000000000000000
		2	168	1253.000000000000000
		3	305	1000.416666666666600
		4	179	994.333333333333400
		5	242	984.000000000000000
	Lowest	1	661	542.942857142857200
		2	507	544.785000000000000
		3	570	553.666666666666600
		4	204	641.625000000000000
		5	136	644.125000000000000

Tests of Normality

Kolmogorov-Smirnov[a]	Shapiro-Wilk

	Statistic	df	Sig.	Statistic	df	Sig.
Cost/day	.346	29	<.001	.525	29	<.001

a. Lilliefors Significance Correction

Cost/day

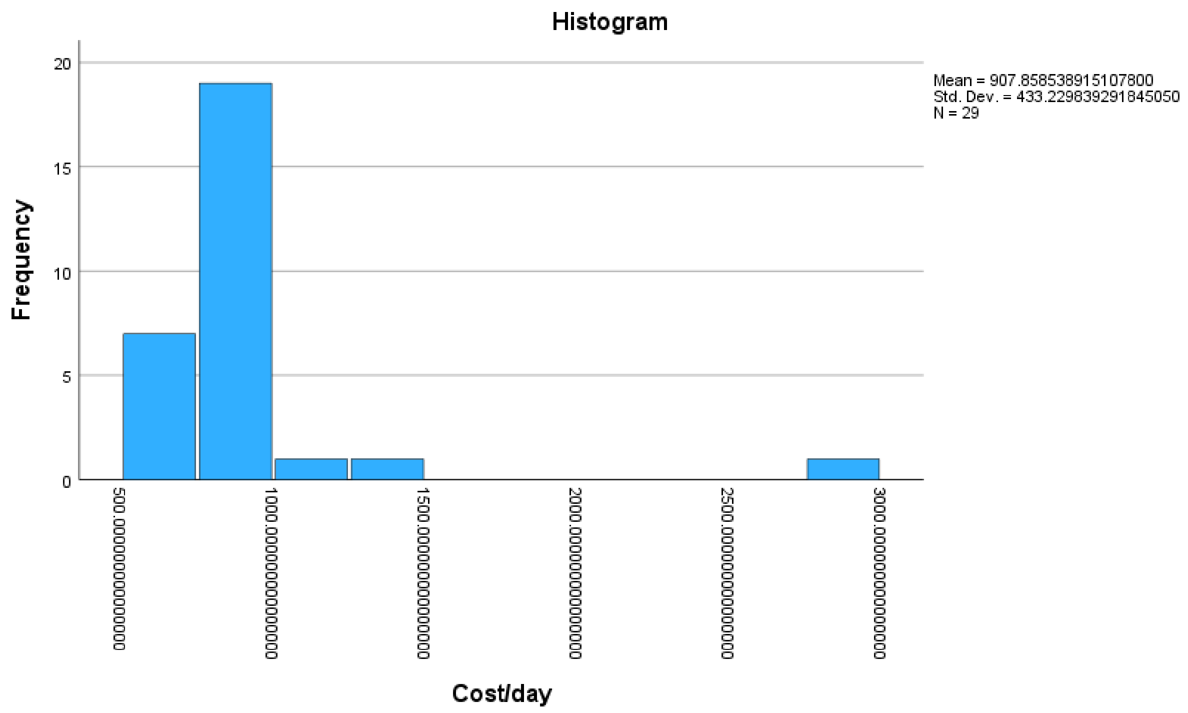


```
Cost/day Stem-and-Leaf Plot

 Frequency     Stem &  Leaf

     3.00        5 .  445
     3.00        6 .  448
     6.00        7 .  057889
     6.00        8 .  125799
     8.00        9 .  34566789
     1.00       10 .  0
      .00       11 .
     1.00       12 .  5
```

```
    1.00 Extremes    (>=2998)

Stem width:  100.0000
Each leaf:        1 case(s)
```

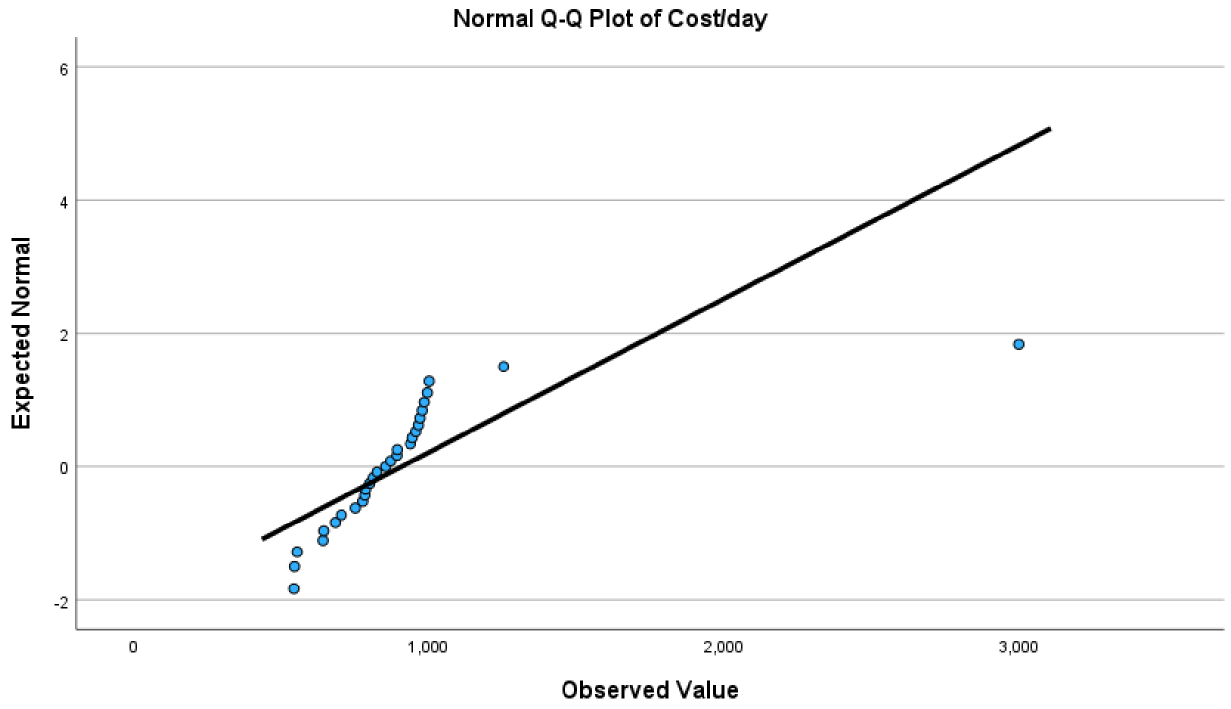

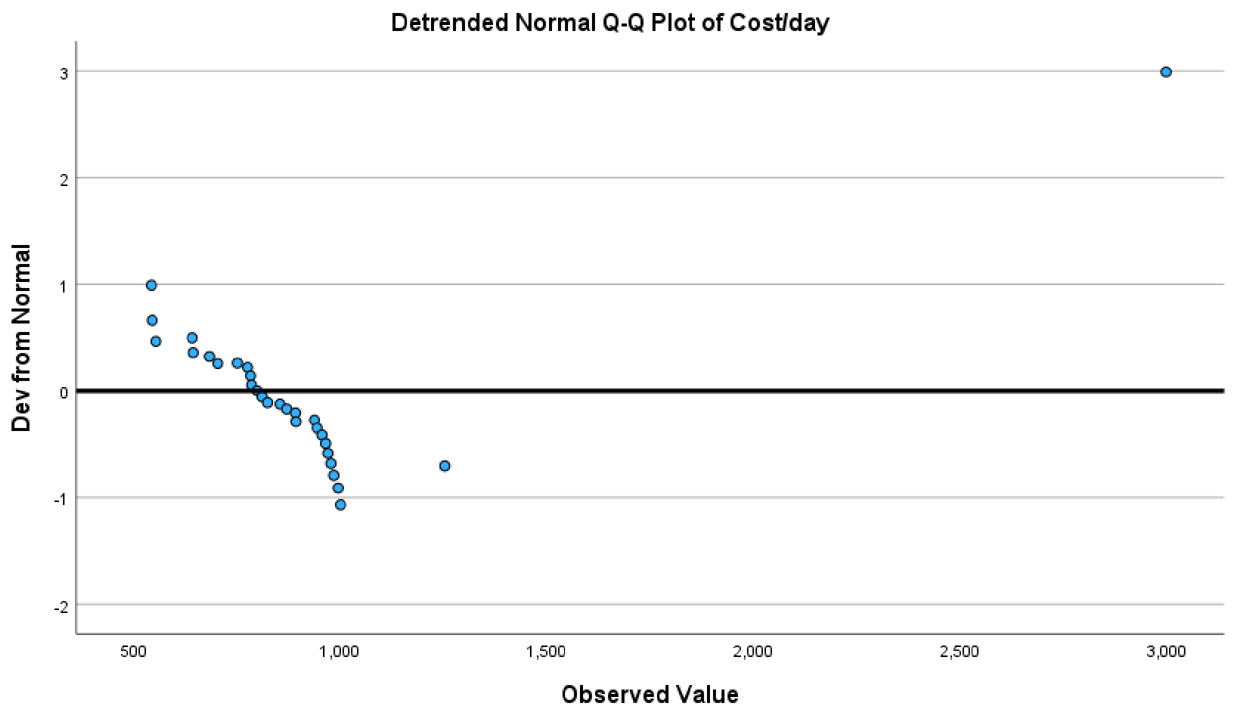
Detrended Normal Q-Q Plot of Cost/day
Dev from Normal
Observed Value
3
2
1
0
-1
-2
500
1,000
1,500
2,000
2,500
3,000

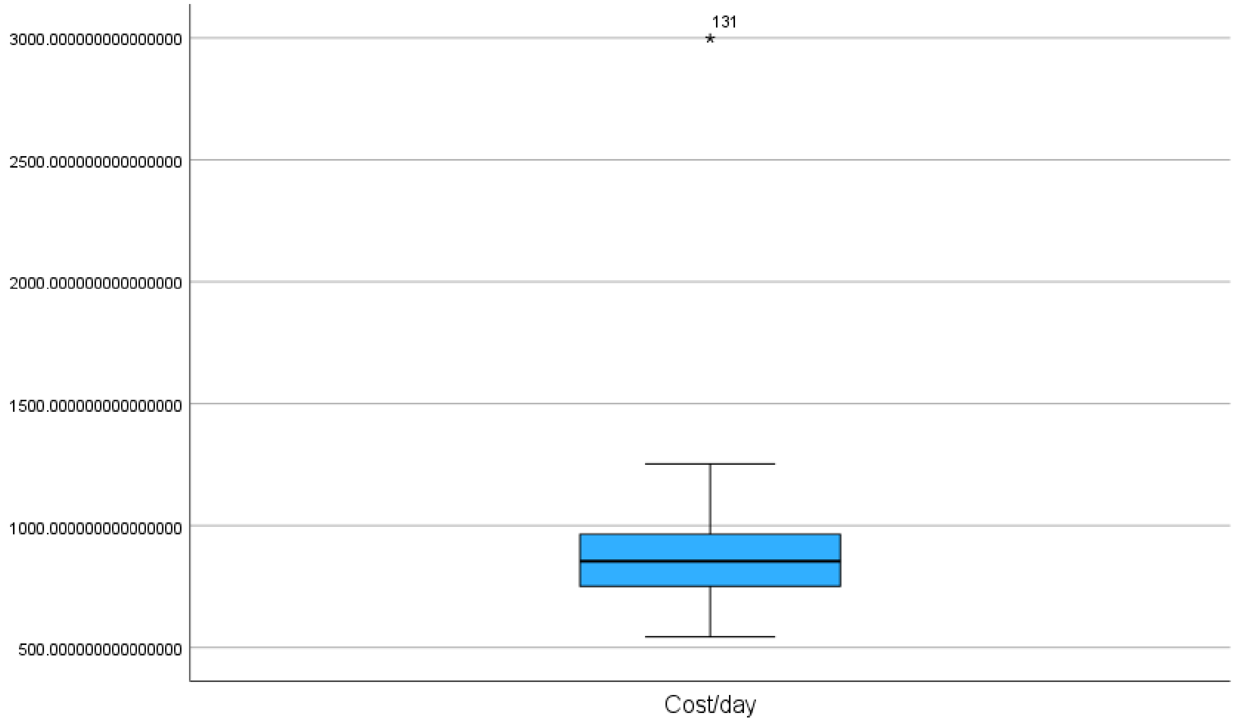
131
3000.000000000000000
2500.000000000000000
2000.000000000000000
1500.000000000000000
1000.000000000000000
500.000000000000000
Cost/day

Explore ALCOHOL

Notes

Output Created		08-MAY-2023 00:10:15
Comments		
Input	Data	C:\Users\paho9\OneDrive\Documente\Doctorat - Stratificarea severității și predicția prognosticului în faza incipientă a Pancreatitei Acute\Registru Pancreatite Acute - BUC-API\Baza date nou\Articole\Articol cost\DB_corect_COST.sav
	Active Dataset	DataSet1
	Filter	Etiology = 1 (FILTER)
	Weight	<none>
	Split File	<none>
	N of Rows in Working Data File	517
Missing Value Handling	Definition of Missing	User-defined missing values for dependent variables are treated as missing.
	Cases Used	Statistics are based on cases with no missing values for any dependent variable or factor used.

Syntax		EXAMINE VARIABLES=Costday<br>/PLOT BOXPLOT STEMLEAF HISTOGRAM NPPLOT<br>/COMPARE GROUPS<br>/STATISTICS DESCRIPTIVES EXTREME<br>/CINTERVAL 95<br>/MISSING LISTWISE<br>/NOTOTAL.
Resources	Processor Time	00:00:00.53
	Elapsed Time	00:00:00.74

Case Processing Summary

	Cases					
	Valid		Missing		Total	
	N	Percent	N	Percent	N	Percent
Cost/day	517	100.0%	0	0.0%	517	100.0%

Descriptives

			Statistic	Std. Error
Cost/day	Mean		845.331333690114800	17.292478327077360
	95% Confidence Interval for Mean	Lower Bound	811.359014461406400	
		Upper Bound	879.303652918823200	
	5% Trimmed Mean		805.548449144800100	
	Median		753.600000000000000	
	Variance		154598.410	
	Std. Deviation		393.190043185215530	

		Statistic	Std. Error
	Minimum	250.3580000000 00000	
	Maximum	5974.465000000 000000	
	Range	5724.107000000 000000	
	Interquartile Range	292.8000793650 79340	
	Skewness	5.818	.107
	Kurtosis	61.817	.214

Extreme Values

			Case Number	Value
Cost/day	Highest	1	1014	5974.465000000 000000
		2	370	3663.000000000 000000
		3	45	2563.800000000 000000
		4	311	2459.000000000 000000
		5	918	2292.116000000 000000
	Lowest	1	492	250.3580000000 00000
		2	206	252.0000000000 00000
		3	257	277.4444444444 44460
		4	464	278.7233333333 33300
		5	232	291.5000000000 00000

Tests of Normality

Kolmogorov-Smirnov[a]	Shapiro-Wilk

	Statistic	df	Sig.	Statistic	df	Sig.
Cost/day	.172	517	<.001	.629	517	<.001

a. Lilliefors Significance Correction

Cost/day

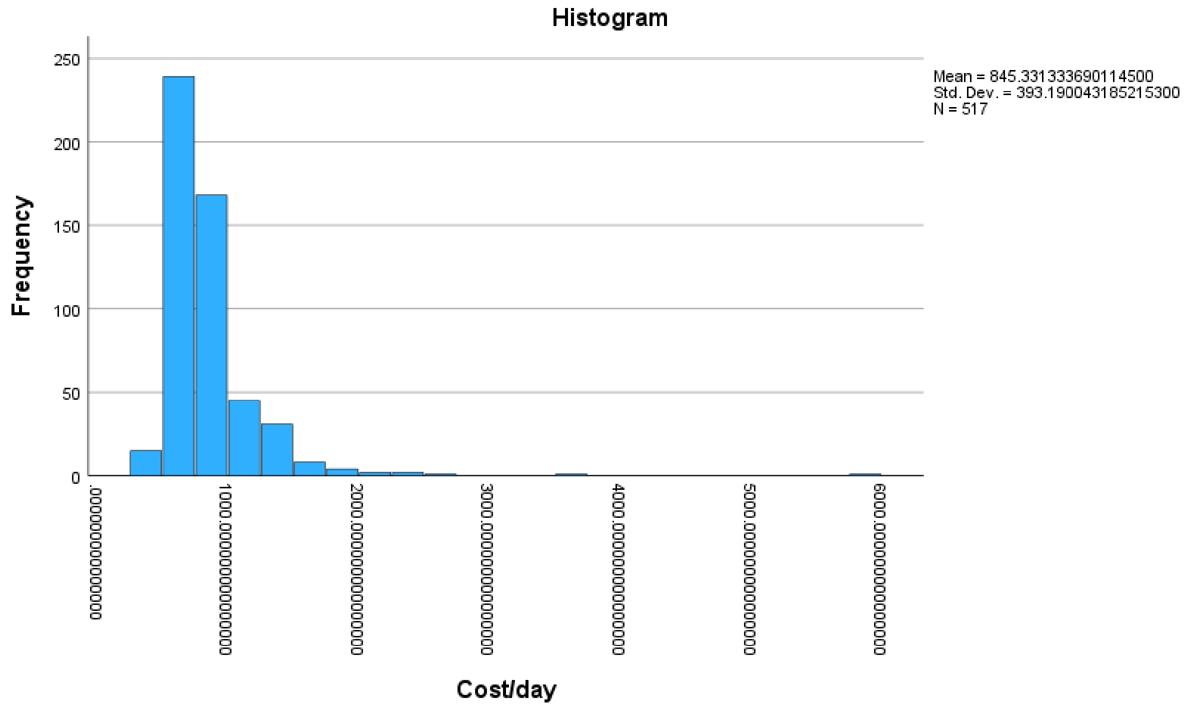


```
Cost/day Stem-and-Leaf Plot

 Frequency     Stem &  Leaf

     5.00        2 .  57&
     3.00        3 .  2&
     7.00        4 .  49&
    57.00        5 .  0223334455566667788899999&
   124.00        6 .  00000011111222222223333333344444555556666666777777888899999
   108.00        7 .  000001111111122222233334444455555556666667778888899
    69.00        8 .  00001111222333444445566778889999
    49.00        9 .  00022333455556666778889&
```

```
   15.00       10 .  02248&
   23.00       11 .  02344557&
   14.00       12 .  34579&&
   12.00       13 .  0025&
   31.00 Extremes    (>=1381)

Stem width:  100.0000
Each leaf:        2 case(s)


& denotes fractional leaves.
```

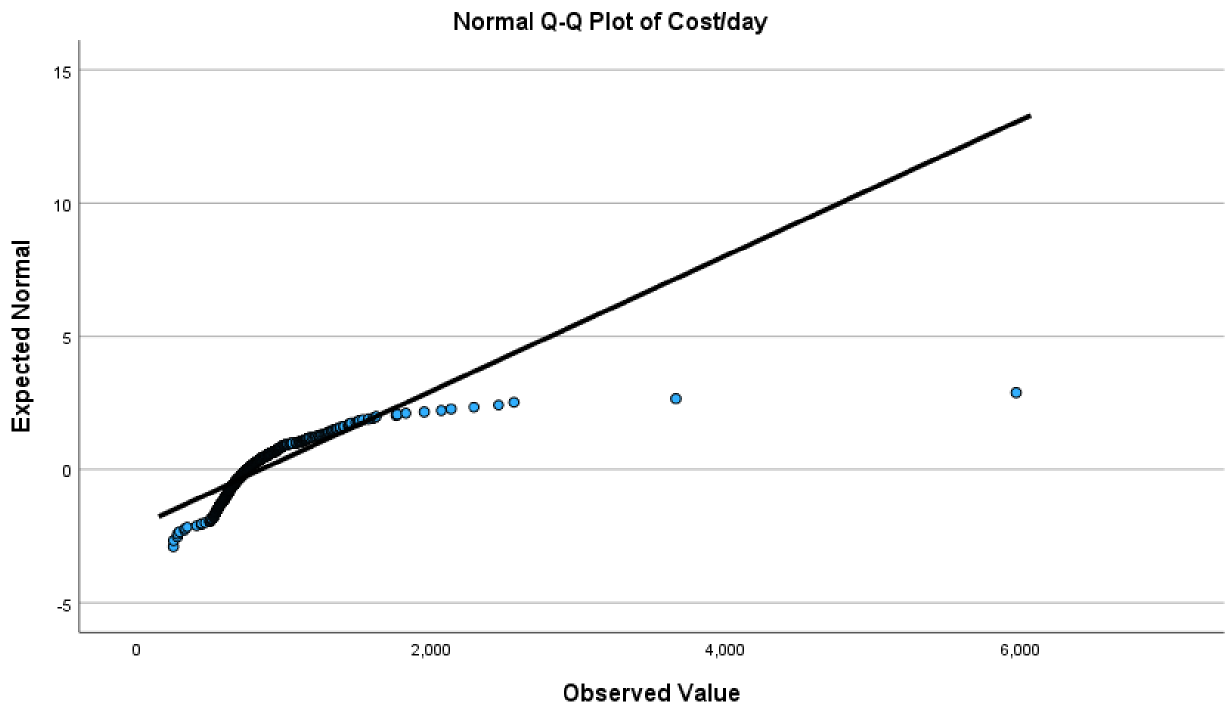

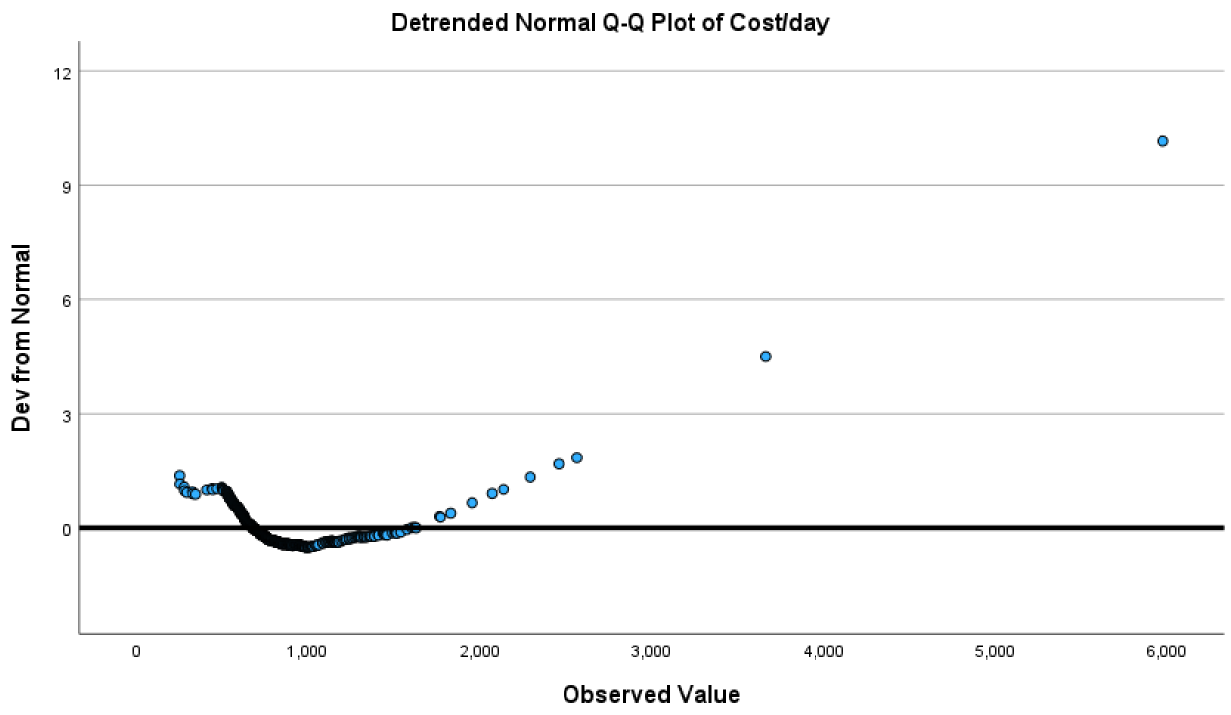

Detrended Normal Q-Q Plot of Cost/day
Dev from Normal
12
9
6
3
0
0
1,000
2,000
3,000
4,000
5,000
6,000
Observed Value

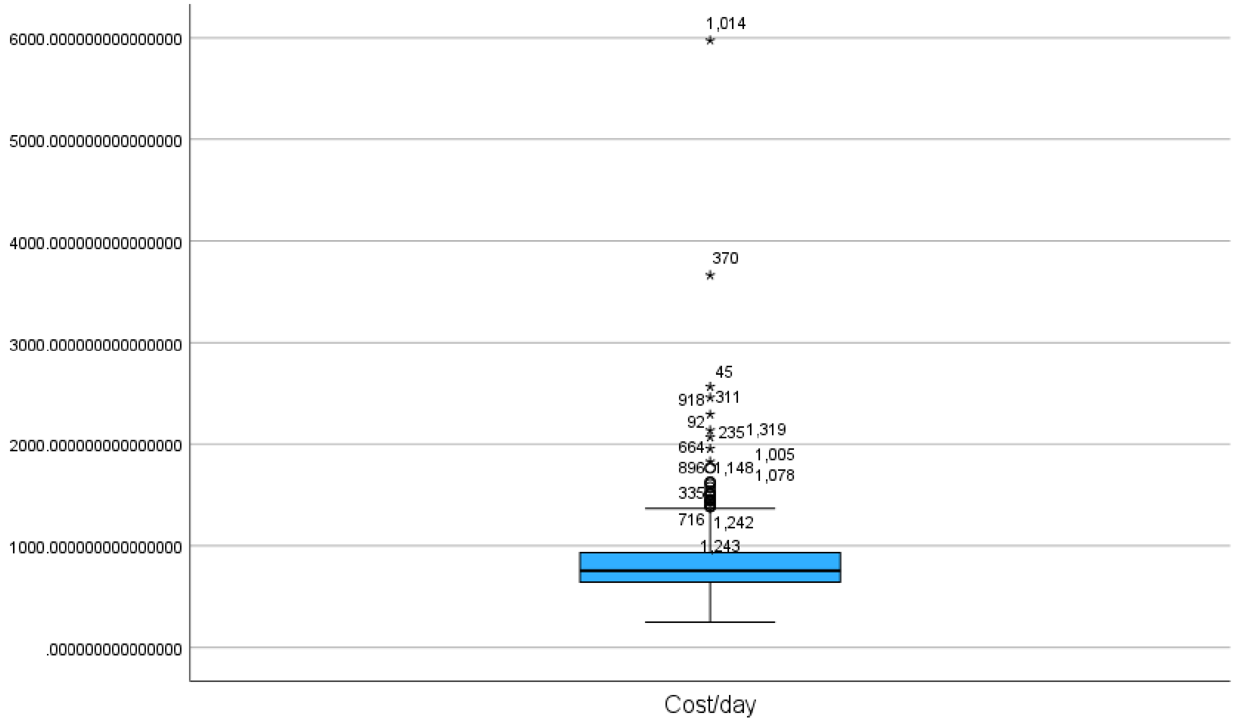

6000.000000000000000
5000.000000000000000
4000.000000000000000
3000.000000000000000
2000.000000000000000
1000.000000000000000
.000000000000000
1,014
370
45
918
311
92
235
1,319
664
1,005
896
1,148
1,078
335
716
1,242
1,243
Cost/day

Explore BILIARY

Notes

Output Created		08-MAY-2023 00:11:03
Comments		
Input	Data	C:\Users\paho9\OneDrive\Documente\Doctorat - Stratificarea severității și predicția prognosticului în faza incipientă a Pancreatitei Acute\Registru Pancreatite Acute - BUC-API\Baza date nou\Articole\Articol cost\DB_corect_COST.sav
	Active Dataset	DataSet1
	Filter	Etiology = 2 (FILTER)
	Weight	<none>
	Split File	<none>
	N of Rows in Working Data File	509
Missing Value Handling	Definition of Missing	User-defined missing values for dependent variables are treated as missing.
	Cases Used	Statistics are based on cases with no missing values for any dependent variable or factor used.

Syntax		EXAMINE VARIABLES=Costday<br>/PLOT BOXPLOT STEMLEAF HISTOGRAM NPPLOT<br>/COMPARE GROUPS<br>/STATISTICS DESCRIPTIVES EXTREME<br>/CINTERVAL 95<br>/MISSING LISTWISE<br>/NOTOTAL.
Resources	Processor Time	00:00:00.53
	Elapsed Time	00:00:00.70

Case Processing Summary

	Cases					
	Valid		Missing		Total	
	N	Percent	N	Percent	N	Percent
Cost/day	509	100.0%	0	0.0%	509	100.0%

Descriptives

			Statistic	Std. Error
Cost/day	Mean		2148.010938963875600	755.629005383380200
	95% Confidence Interval for Mean	Lower Bound	663.468368106802500	
		Upper Bound	3632.553509820948400	
	5% Trimmed Mean		1070.504171498728000	
	Median		1048.432000000000000	
	Variance		290626373.632	
	Std. Deviation		9223.372036854777000	

	Minimum	306.452666666666700	
	Maximum	9223.372036854777000	
	Range	9223.372036854777000	
	Interquartile Range	289.806547619047600	
	Skewness	16.819	.108
	Kurtosis	290.774	.216

Extreme Values

			Case Number	Value
Cost/day	Highest	1	1049	9223.372036854777000
		2	1018	9223.372036854777000
		3	1144	3649.810000000000000
		4	913	3575.529310344828000
		5	1368	3097.000000000000000
	Lowest	1	916	306.452666666666700
		2	1473	355.864285714285760
		3	802	371.770000000000000
		4	752	533.016666666666700
		5	239	555.000000000000000

Tests of Normality

Kolmogorov-Smirnov[a]	Shapiro-Wilk

	Statistic	df	Sig.	Statistic	df	Sig.
Cost/day	.483	509	<.001	.043	509	<.001

a. Lilliefors Significance Correction

Cost/day

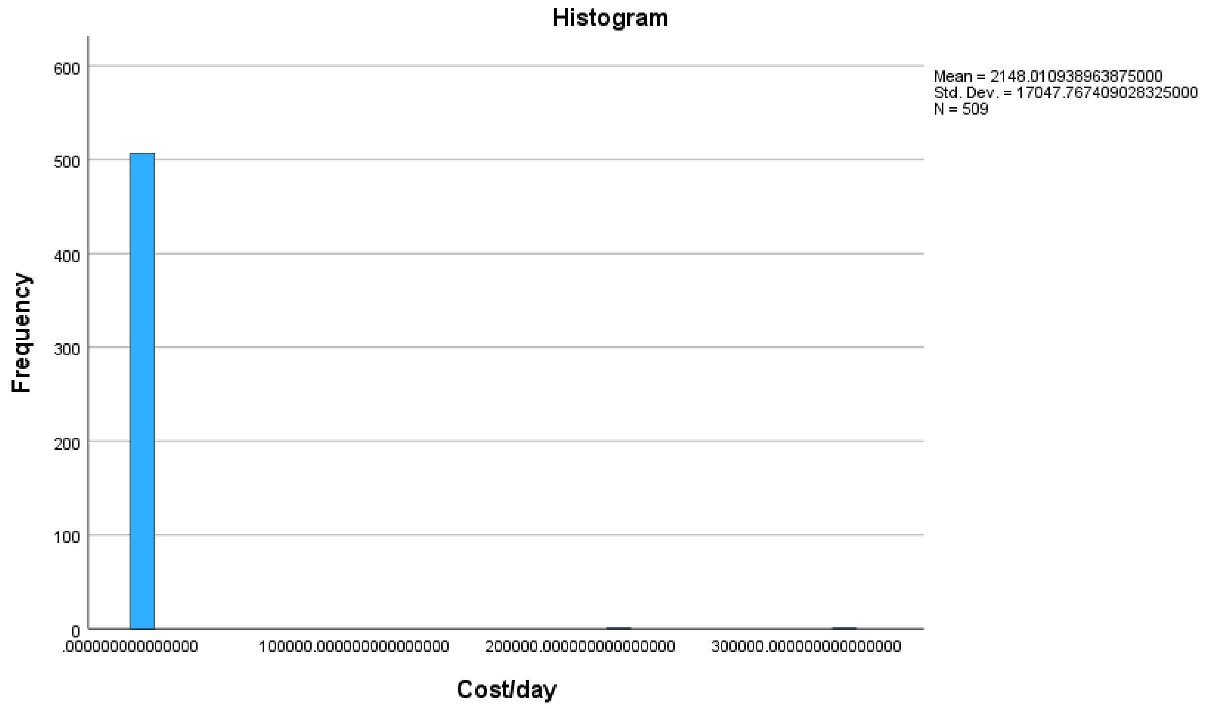


```
Cost/day Stem-and-Leaf Plot

 Frequency    Stem &  Leaf

     3.00 Extremes    (=<372)
    11.00        5 .  6779&
    23.00        6 .  2344556789&
    34.00        7 .  012334445677899
    35.00        8 .  1223344455679999&
    93.00        9 .  0001122233333344444455566666777778888999999
    98.00       10 .  000111111111222223333444445566677777888899999
    69.00       11 .  00000111222333334455556666777899
```

```
   58.00       12 .  00001112222334566677777788999
   32.00       13 .  00011222334567&
   17.00       14 .  0456679&
    6.00       15 .  1&&
    2.00       16 .  1
   28.00 Extremes    (>=1661)

Stem width:  100.0000
Each leaf:        2 case(s)


& denotes fractional leaves.
```

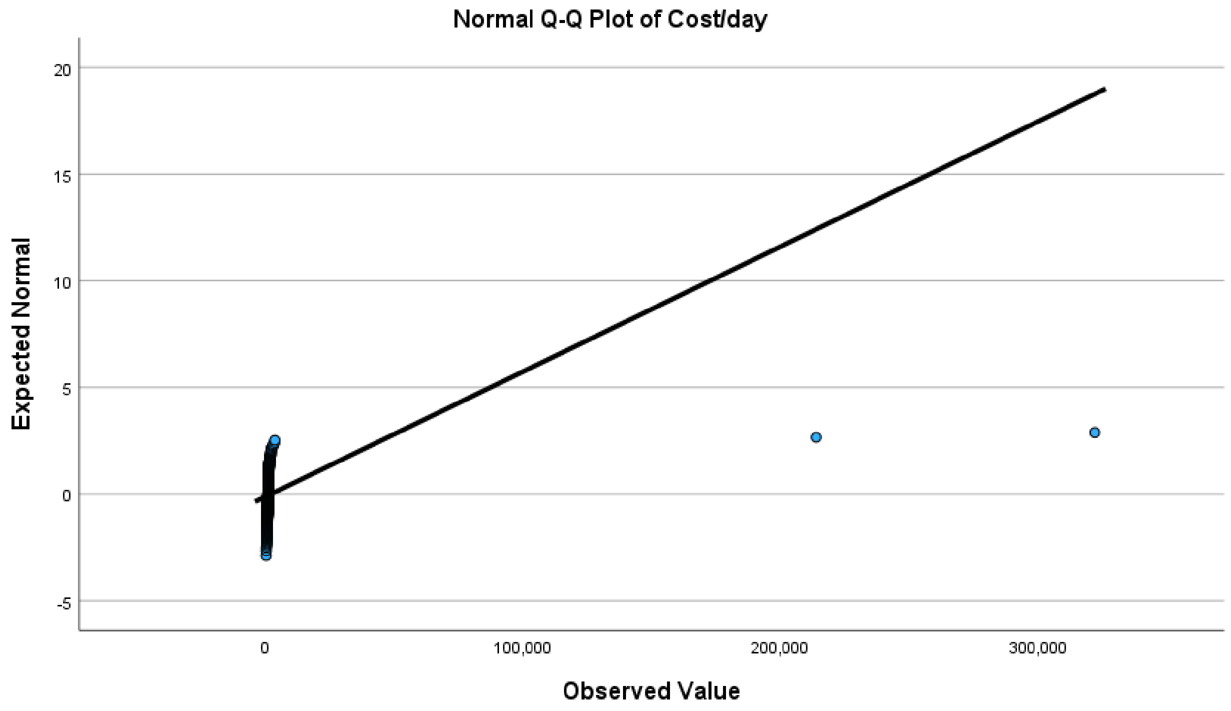

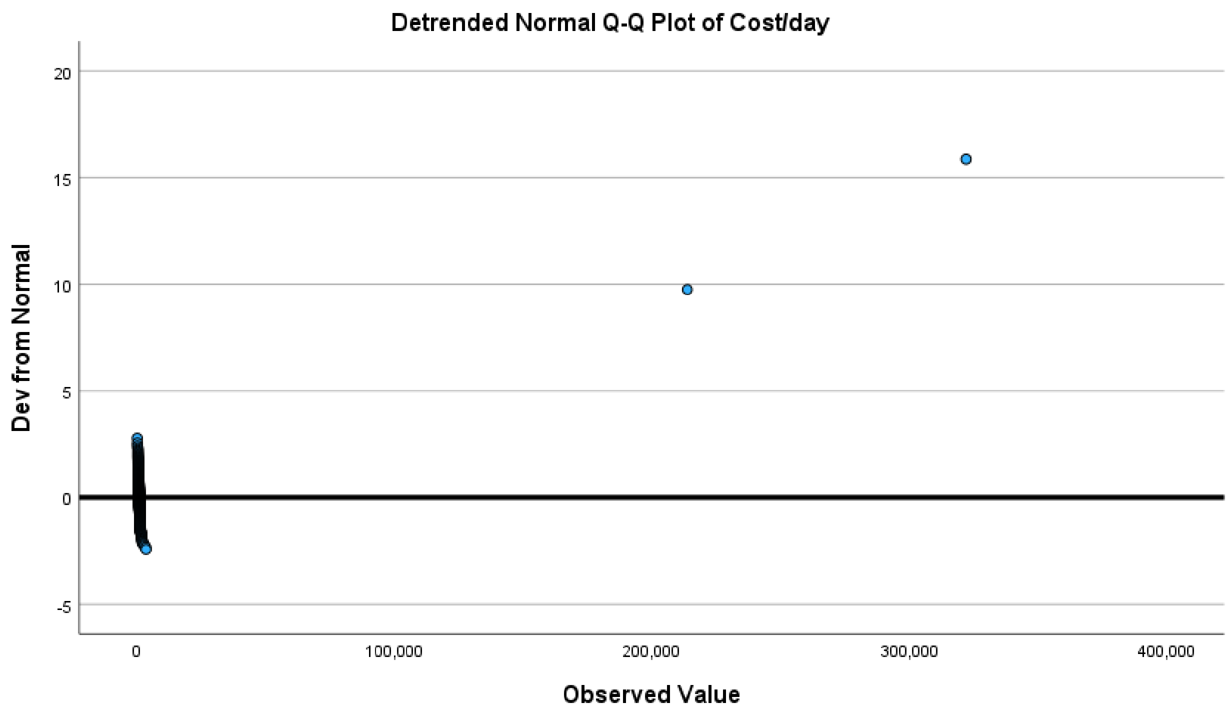

Detrended Normal Q-Q Plot of Cost/day
Dev from Normal
20
15
10
5
0
-5
0
100,000
200,000
300,000
400,000
Observed Value

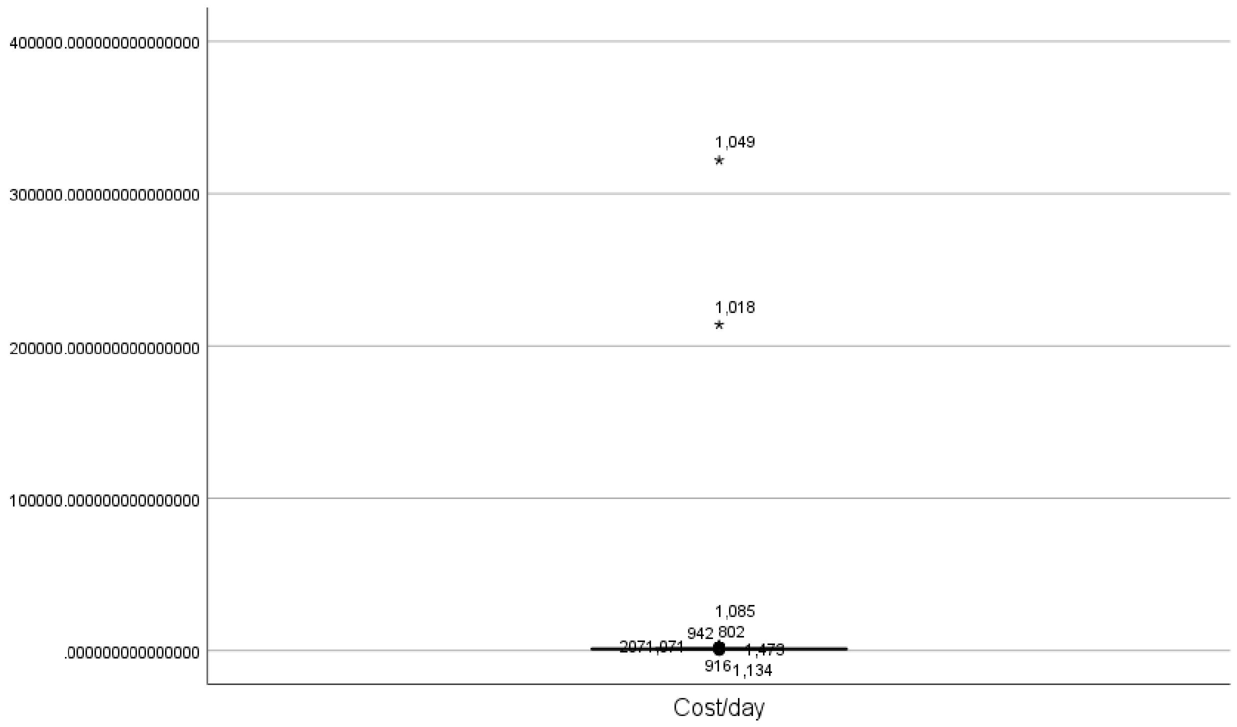

400000.000000000000000
300000.000000000000000
200000.000000000000000
100000.000000000000000
.000000000000000
1,049
1,018
1,085
942
802
2071,071
916
1,134
Cost/day

Explore HYPERTRIGLICERIDEMIA

Notes

Output Created		08-MAY-2023 00:12:52
Comments		
Input	Data	C:\Users\paho9\OneDrive\Documente\Doctorat - Stratificarea severității și predicția prognosticului în faza incipientă a Pancreatitei Acute\Registru Pancreatite Acute - BUC-API\Baza date nou\Articole\Articol cost\DB_corect_COST.sav
	Active Dataset	DataSet1
	Filter	Etiology = 3 (FILTER)
	Weight	<none>
	Split File	<none>
	N of Rows in Working Data File	80
Missing Value Handling	Definition of Missing	User-defined missing values for dependent variables are treated as missing.
	Cases Used	Statistics are based on cases with no missing values for any dependent variable or factor used.

Syntax		EXAMINE VARIABLES=Costday<br>/PLOT BOXPLOT STEMLEAF HISTOGRAM NPPLOT<br>/COMPARE GROUPS<br>/STATISTICS DESCRIPTIVES EXTREME<br>/CINTERVAL 95<br>/MISSING LISTWISE<br>/NOTOTAL.
Resources	Processor Time	00:00:00.74
	Elapsed Time	00:00:01.07

Case Processing Summary

	Cases					
	Valid		Missing		Total	
	N	Percent	N	Percent	N	Percent
Cost/day	80	100.0%	0	0.0%	80	100.0%

Descriptives

			Statistic	Std. Error
Cost/day	Mean		1051.973464044286400	87.537925892911390
	95% Confidence Interval for Mean	Lower Bound	877.733581047667400	
		Upper Bound	1226.213347040905300	
	5% Trimmed Mean		924.674661330813800	
	Median		832.990196078431400	
	Variance		613031.078	
	Std. Deviation		782.963011623555400	

	Minimum	535.571428571428600	
	Maximum	6290.557500000000000	
	Range	5754.986071428571000	
	Interquartile Range	381.037633928571270	
	Skewness	4.656	.269
	Kurtosis	26.842	.532

Extreme Values

			Case Number	Value
Cost/day	Highest	1	702	6290.557500000000000
		2	852	3786.594411764706000
		3	1279	2810.101111111111000
		4	611	2399.095238095238000
		5	1065	2091.200000000000000
	Lowest	1	3	535.571428571428600
		2	1200	575.363636363636400
		3	640	591.200000000000000
		4	22	592.818181818181900
		5	449	615.000000000000000

Tests of Normality

Kolmogorov-Smirnov[a]	Shapiro-Wilk

	Statistic	df	Sig.	Statistic	df	Sig.
Cost/day	.262	80	<.001	.512	80	<.001

a. Lilliefors Significance Correction

Cost/day

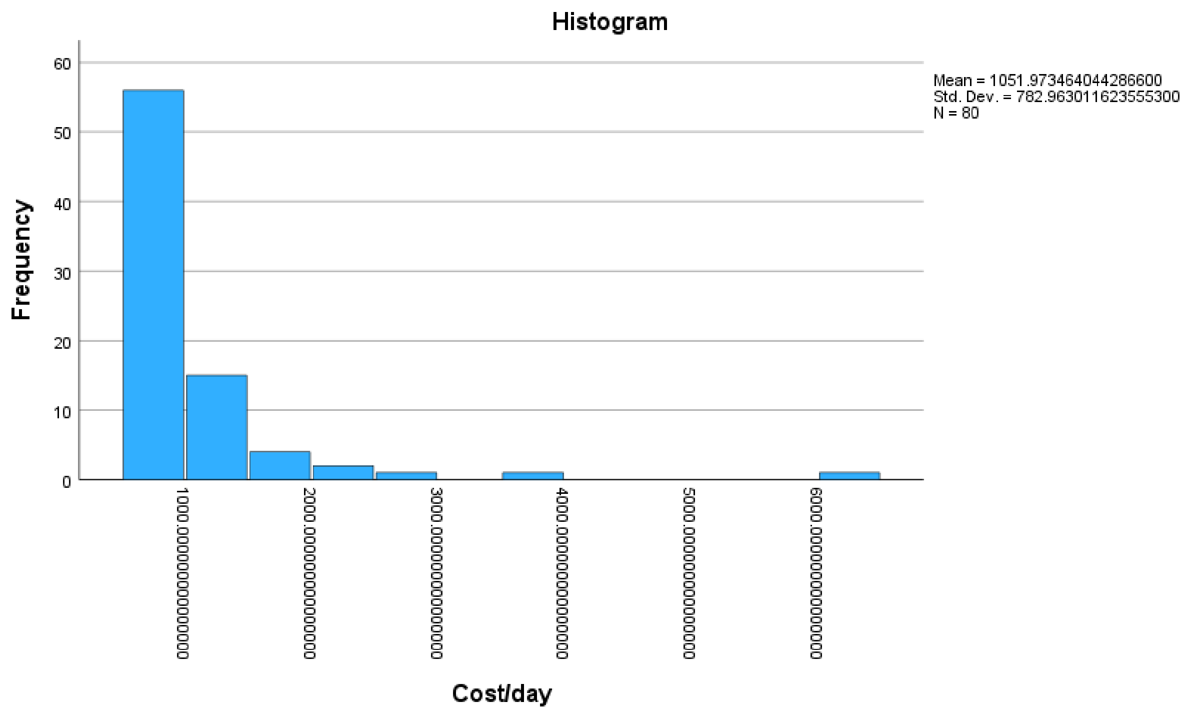


```
Cost/day Stem-and-Leaf Plot

 Frequency    Stem &  Leaf

     4.00        0 .  5555
    32.00        0 .  66666666666666666777777777777777
    20.00        0 .  88888888888999999999
     8.00        1 .  00000011
     6.00        1 .  222223
     3.00        1 .  455
     7.00 Extremes    (>=1657)
```

```
Stem width:  1000.000
Each leaf:        1 case(s)
```

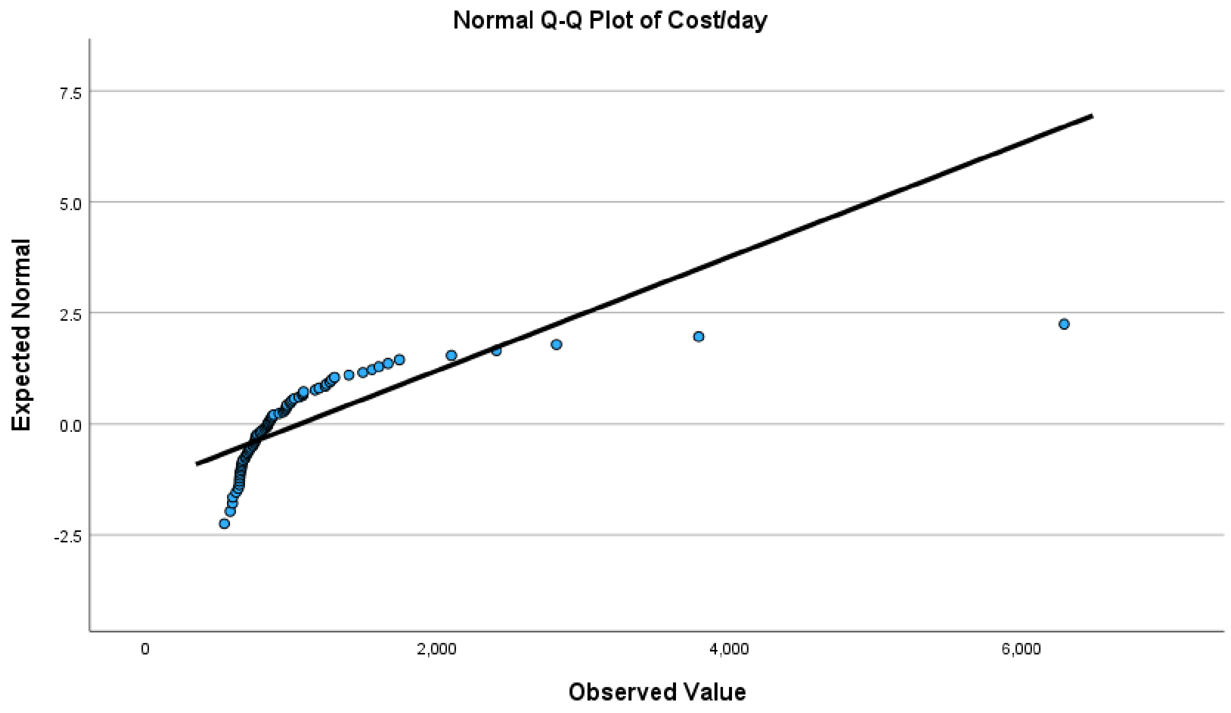

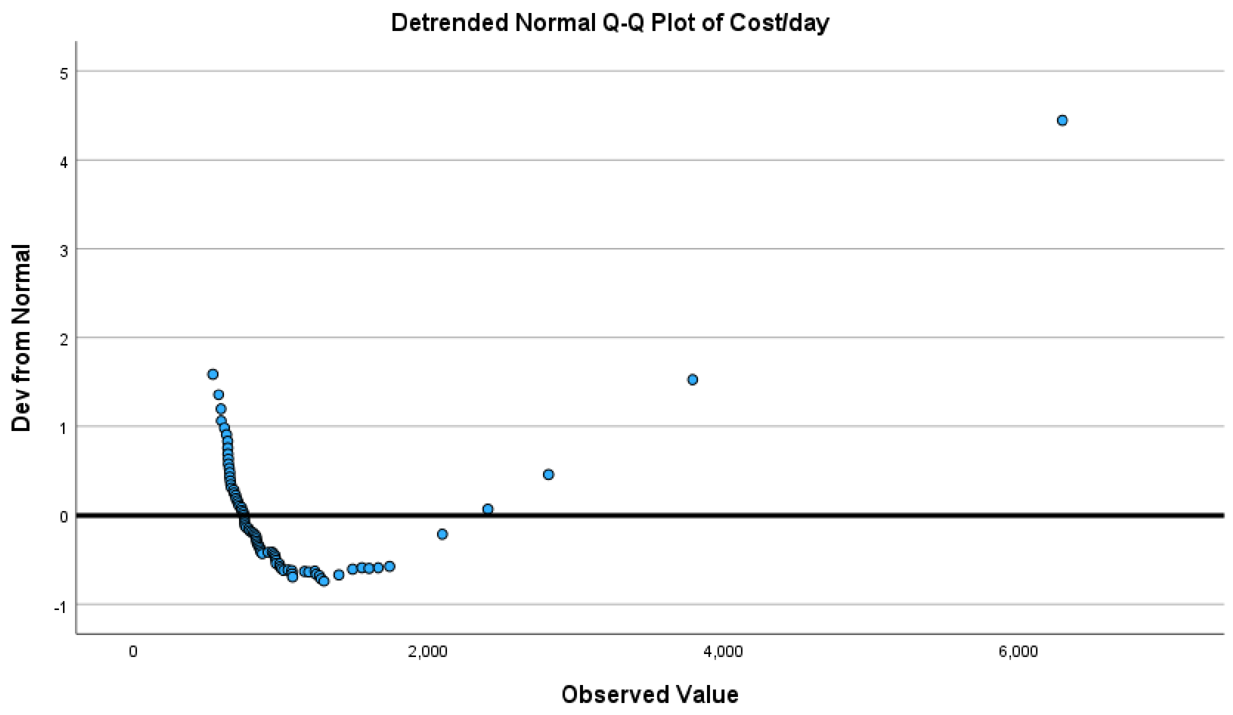

Detrended Normal Q-Q Plot of Cost/day
Dev from Normal
5
4
3
2
1
0
-1
0
2,000
4,000
6,000
Observed Value

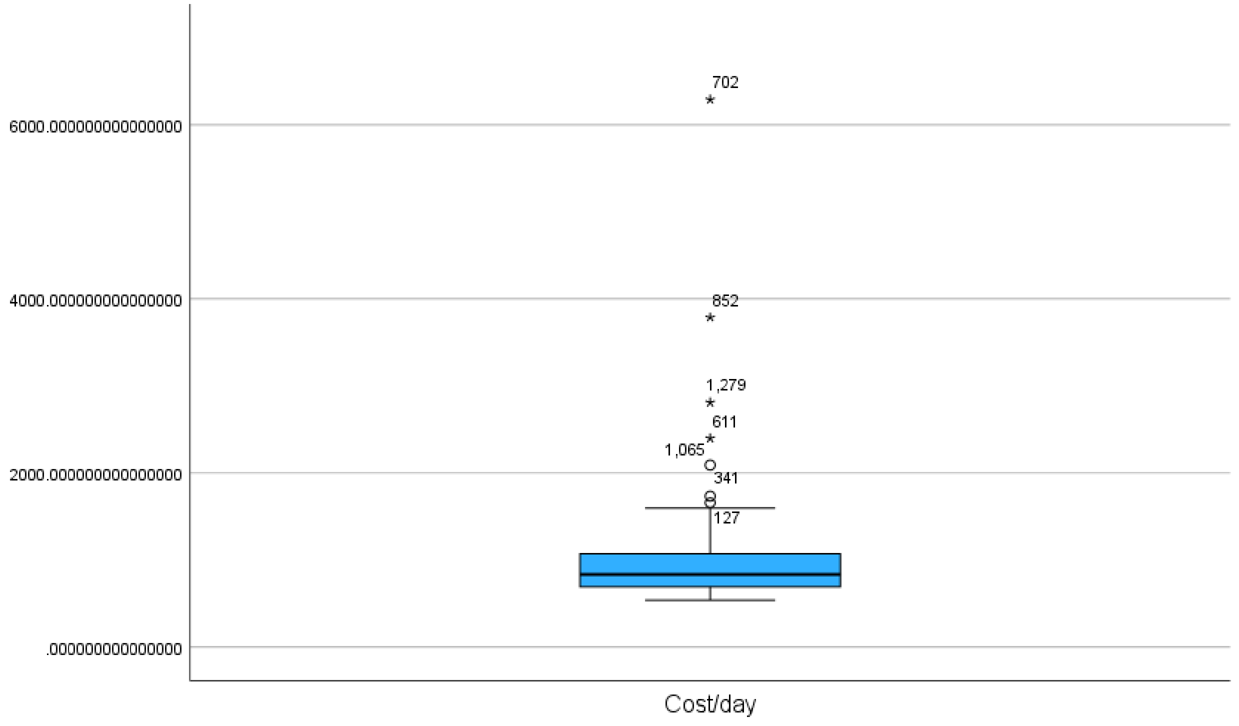

702
852
1,279
611
1,065
341
127
6000.000000000000000
4000.000000000000000
2000.000000000000000
.000000000000000
Cost/day

Explore DIABETES MELLITUS

Notes

Output Created		08-MAY-2023 00:13:21
Comments		
Input	Data	C:\Users\paho9\OneDrive\Documente\Doctorat - Stratificarea severității și predicția prognosticului în faza incipientă a Pancreatitei Acute\Registru Pancreatite Acute - BUC-API\Baza date nou\Articole\Articol cost\DB_corect_COST.sav
	Active Dataset	DataSet1
	Filter	Etiology = 7 (FILTER)
	Weight	<none>
	Split File	<none>
	N of Rows in Working Data File	62
Missing Value Handling	Definition of Missing	User-defined missing values for dependent variables are treated as missing.
	Cases Used	Statistics are based on cases with no missing values for any dependent variable or factor used.

Syntax		EXAMINE VARIABLES=Costday<br>/PLOT BOXPLOT STEMLEAF HISTOGRAM NPPLOT<br>/COMPARE GROUPS<br>/STATISTICS DESCRIPTIVES EXTREME<br>/CINTERVAL 95<br>/MISSING LISTWISE<br>/NOTOTAL.
Resources	Processor Time	00:00:00.44
	Elapsed Time	00:00:00.62

Case Processing Summary

	Cases					
	Valid		Missing		Total	
	N	Percent	N	Percent	N	Percent
Cost/day	62	100.0%	0	0.0%	62	100.0%

Descriptives

			Statistic	Std. Error
Cost/day	Mean		1083.434184583267000	140.911630791324820
	95% Confidence Interval for Mean	Lower Bound	801.663964252886200	
		Upper Bound	1365.204404913648000	
	5% Trimmed Mean		894.810930472532500	
	Median		826.556818200000000	
	Variance		1231077.437	
	Std. Deviation		1109.539290390736800	

	Minimum	474.714285700000000	
	Maximum	8662.000000000000000	
	Range	8187.285714300000000	
	Interquartile Range	370.946071428571400	
	Skewness	5.753	.304
	Kurtosis	37.225	.599

Extreme Values

			Case Number	Value
Cost/day	Highest	1	1219	8662.000000000000000
		2	542	4022.368571428571600
		3	310	2806.000000000000000
		4	1211	1581.250000000000000
		5	758	1478.292727272727500
	Lowest	1	386	474.714285700000000
		2	565	503.494999999999950
		3	570	553.666666666666600
		4	85	621.000000000000000
		5	180	623.142857142857100

Tests of Normality

Kolmogorov-Smirnov[a]	Shapiro-Wilk

	Statistic	df	Sig.	Statistic	df	Sig.
Cost/day	.324	62	<.001	.371	62	<.001

a. Lilliefors Significance Correction

Cost/day

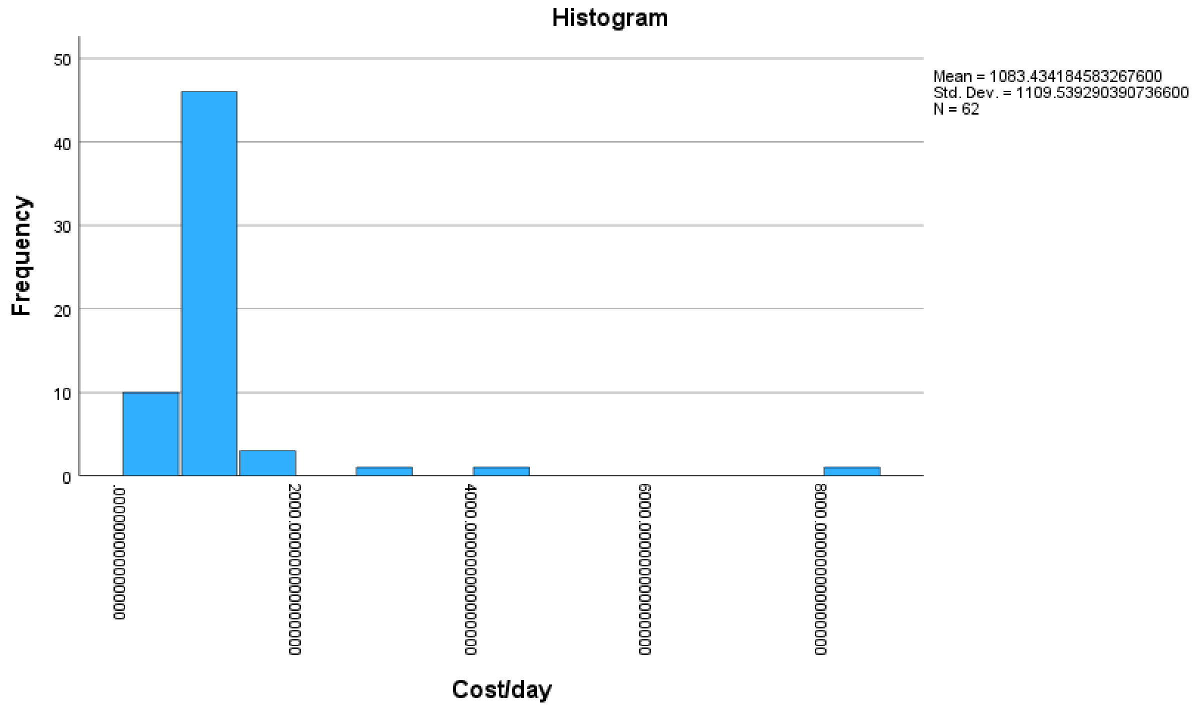


```
Cost/day Stem-and-Leaf Plot

 Frequency    Stem &  Leaf

     3.00        0 .  455
    24.00        0 .  666666666666677777777777
    17.00        0 .  88888888888889999
     7.00        1 .  0000011
     5.00        1 .  22223
     3.00        1 .  445
     3.00 Extremes    (>=2806)
```

```
Stem width:  1000.000
Each leaf:        1 case(s)
```

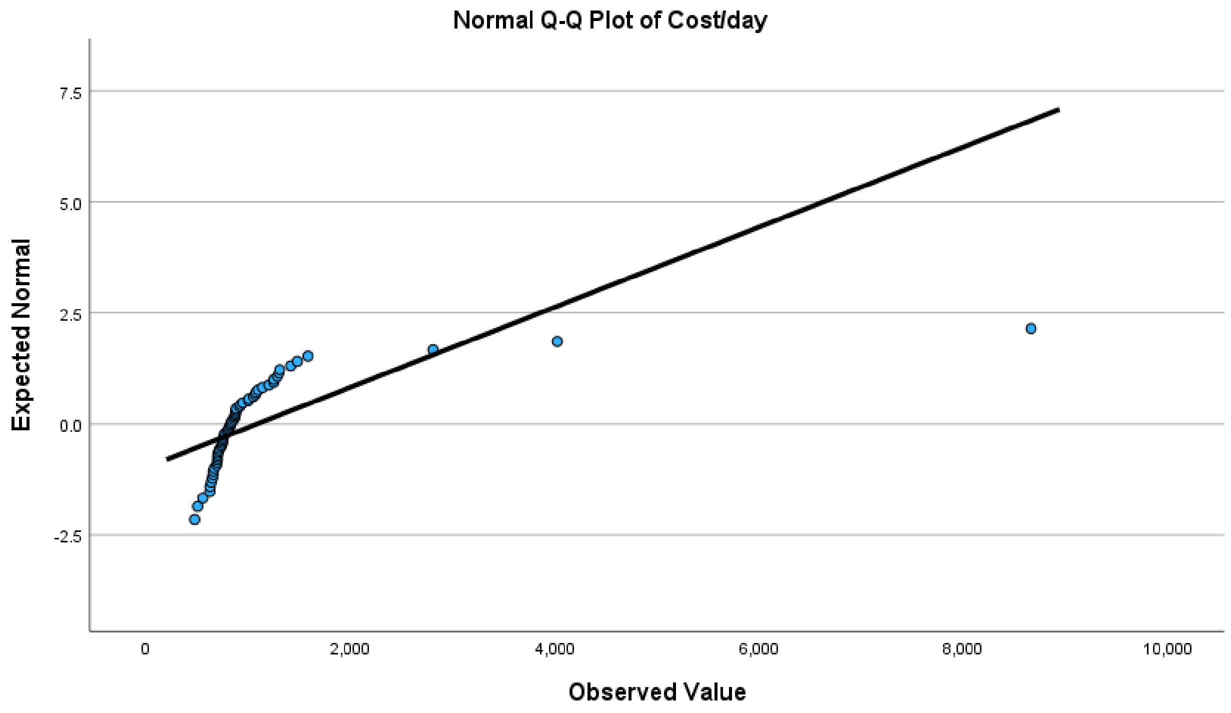

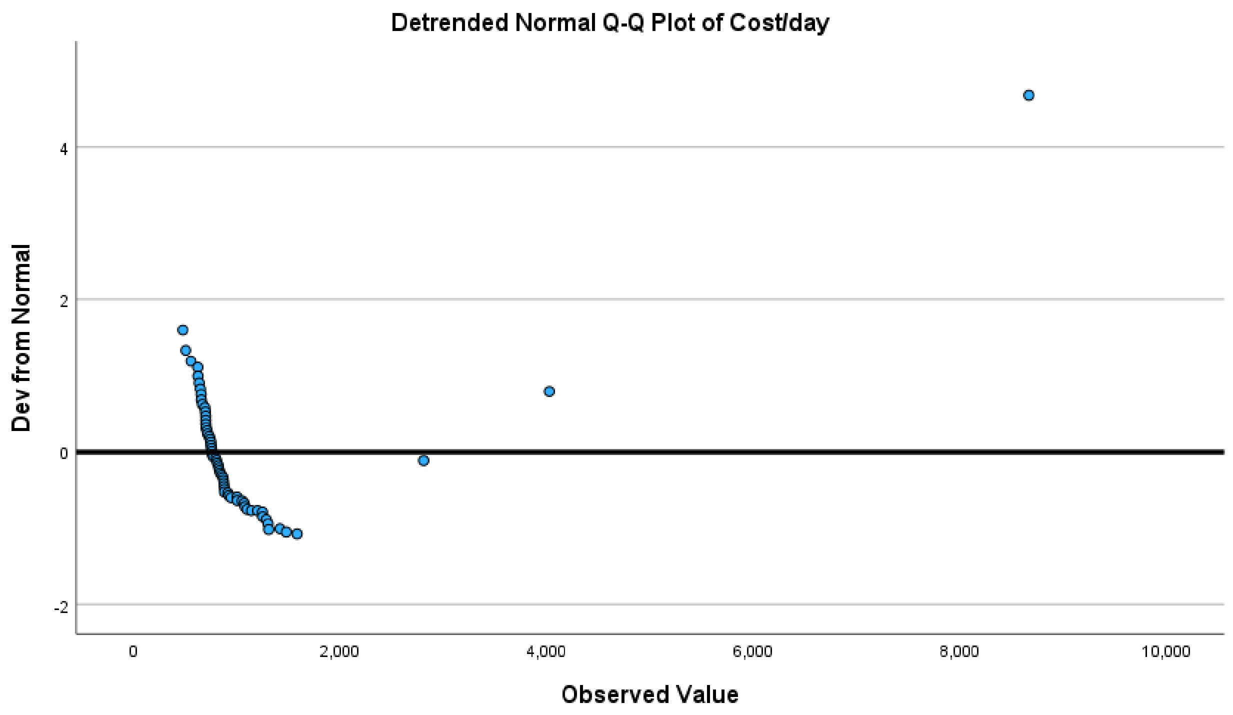
Detrended Normal Q-Q Plot of Cost/day
Dev from Normal
4
2
0
-2
0
2,000
4,000
6,000
8,000
10,000
Observed Value

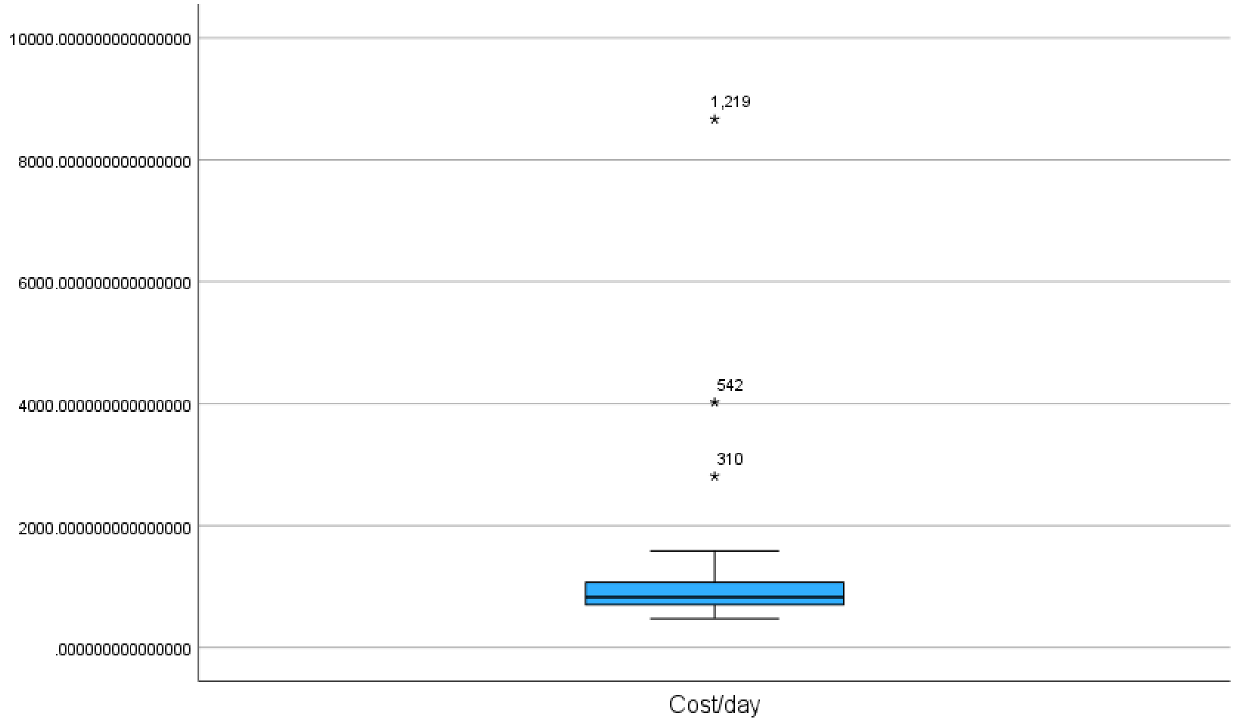
10000.000000000000000
8000.000000000000000
6000.000000000000000
4000.000000000000000
2000.000000000000000
.000000000000000
1,219
542
310
Cost/day

Nonparametric Tests ETIOLOGY

Notes

Output Created		08-MAY-2023 00:22:38
Comments		
Input	Data	C:\Users\paho9\OneDrive\Documente\Doctorat - Stratificarea severității și predicția prognosticului în faza incipientă a Pancreatitei Acute\Registru Pancreatite Acute - BUC-API\Baza date nou\Articole\Articol cost\DB_corect_COST.sav
	Active Dataset	DataSet1
	Filter	Etiology > 0 AND Etiology < 8 (FILTER)
	Weight	<none>
	Split File	<none>
	N of Rows in Working Data File	1168
Syntax		NPTESTS /INDEPENDENT TEST (Costday) GROUP (Etiology) KRUSKAL_WALLIS(COMPARE=PAIRWISE) /MISSING SCOPE=ANALYSIS USERMISSING=EXCLUDE /CRITERIA ALPHA=0.05 CILEVEL=95.
Resources	Processor Time	00:00:00.53
	Elapsed Time	00:00:00.79

Hypothesis Test Summary

Null Hypothesis	Test	Sig.[a,b]	Decision

1	The distribution of Cost/day is the same across categories of Etiology.	Independent-Samples Kruskal-Wallis Test	<.001	Reject the null hypothesis.

a. The significance level is .050.
b. Asymptotic significance is displayed.

Independent-Samples Kruskal-Wallis Test

Cost/day across Etiology

Independent-Samples Kruskal-Wallis Test Summary

Total N	1168
Test Statistic	239.914[a]
Degree Of Freedom	3
Asymptotic Sig.(2-sided test)	<.001

a. The test statistic is adjusted for ties.

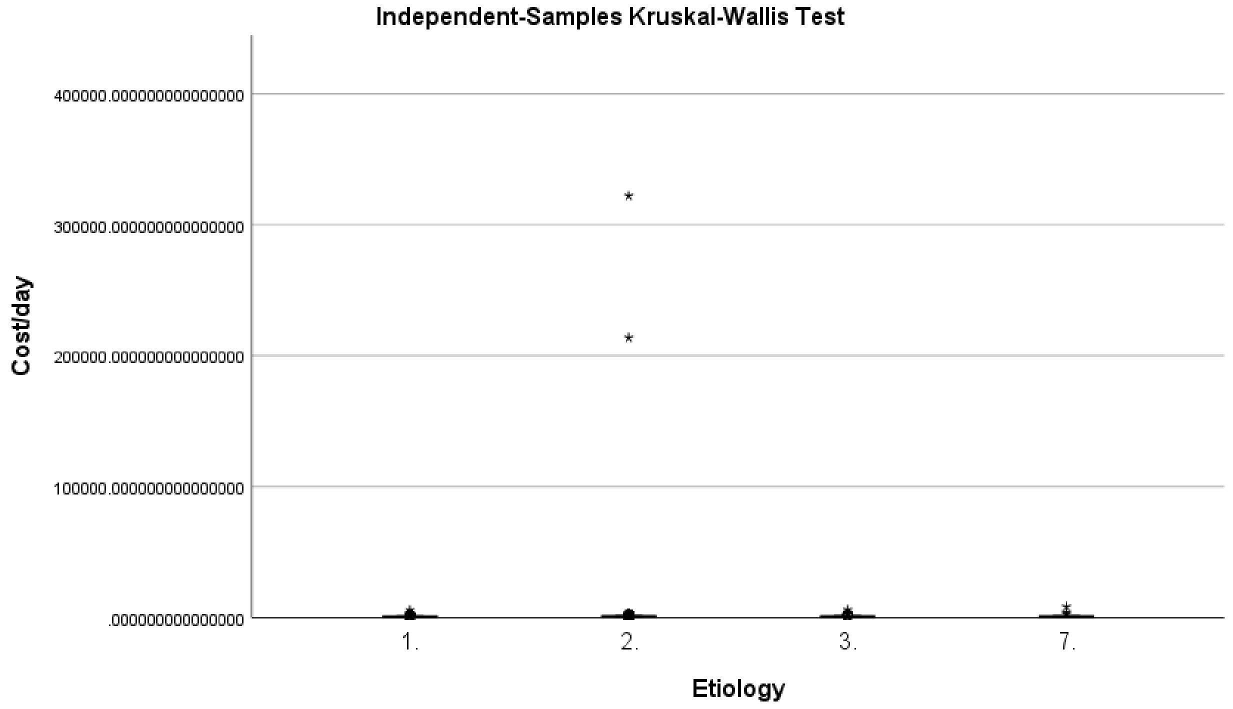

Pairwise Comparisons of Etiology

Sample 1-Sample 2	Test Statistic	Std. Error	Std. Test Statistic	Sig.	Adj. Sig.[a]
Alchool(1)-Diabetes(7)	-108.145	45.335	-2.385	.017	.102
Alchool(1)-Hypertrigliceridemia (3)	-119.377	40.526	-2.946	.003	.019
Alchool (1)-Biliary (2)	-324.592	21.062	-15.411	<.001	.000
Diabetes (7)-Hypertrigliceridemia(3)	11.232	57.074	.197	.844	1.000
Diabetes (7) -Biliary (2)	216.448	45.373	4.770	<.001	.000
Hypertrigliceridemia (3)-Biliary (2)	205.216	40.569	5.058	<.001	.000

Each row tests the null hypothesis that the Sample 1 and Sample 2 distributions are the same.

Asymptotic significances (2-sided tests) are displayed. The significance level is .050.

a. Significance values have been adjusted by the Bonferroni correction for multiple tests.

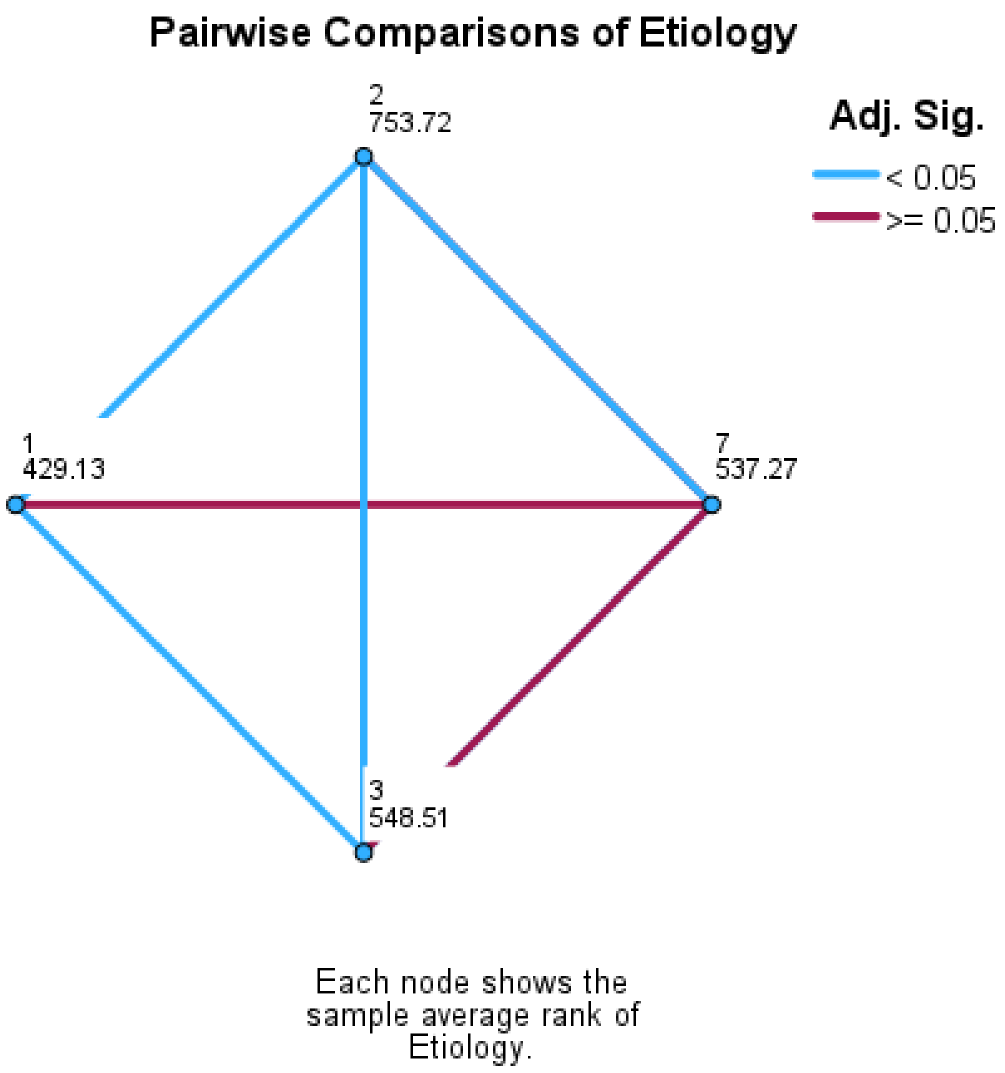

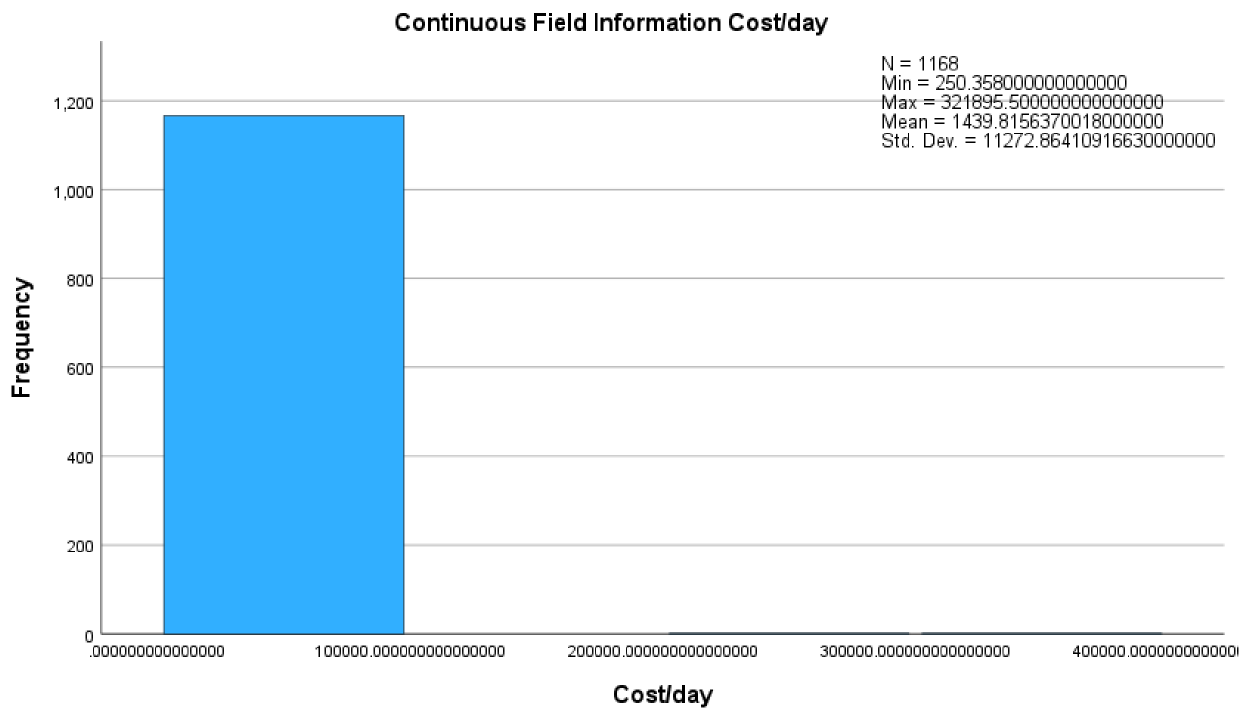

Continuous Field Information Cost/day
N = 1168
Min = 250.358000000000000
Max = 321895.500000000000000
Mean = 1439.8156370018000000
Std. Dev. = 11272.86410916630000000
Frequency
Cost/day

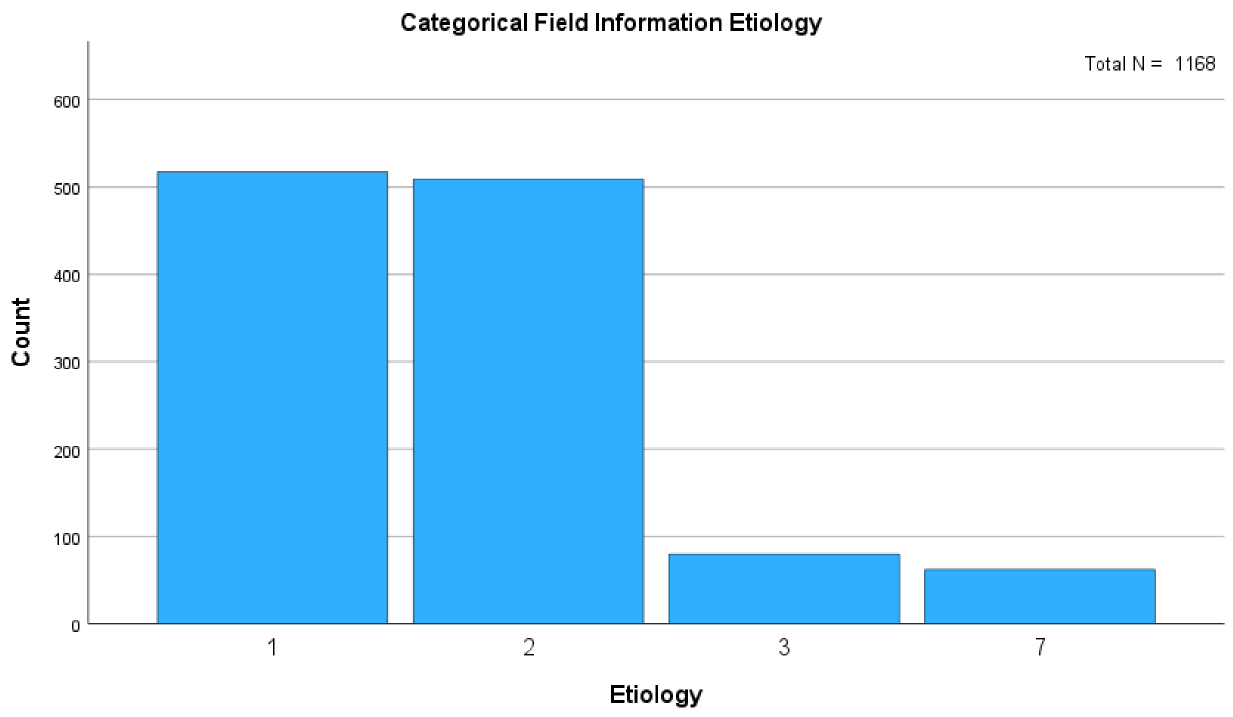

Categorical Field Information Etiology
Total N = 1168
Count
1
2
3
7
Etiology

Explore HIPERTRYGLICERIDEMIA

Notes

Output Created		08-MAY-2023 00:12:52
Comments		
Input	Data	C:\Users\paho9\OneDrive\Documente\Doctorat - Stratificarea severității și predicția prognosticului în faza incipientă a Pancreatitei Acute\Registru Pancreatite Acute - BUC-API\Baza date nou\Articole\Articol cost\DB_corect_COST.sav
	Active Dataset	DataSet1
	Filter	Etiology = 3 (FILTER)
	Weight	<none>
	Split File	<none>
	N of Rows in Working Data File	80
Missing Value Handling	Definition of Missing	User-defined missing values for dependent variables are treated as missing.
	Cases Used	Statistics are based on cases with no missing values for any dependent variable or factor used.
Syntax		EXAMINE VARIABLES=Costday /PLOT BOXPLOT STEMLEAF HISTOGRAM NPPLOT /COMPARE GROUPS /STATISTICS DESCRIPTIVES

		EXTREME<br>/CINTERVAL 95<br>/MISSING LISTWISE<br>/NOTOTAL.
Resources	Processor Time	00:00:00.74
	Elapsed Time	00:00:01.07

Case Processing Summary

	Cases					
	Valid		Missing		Total	
	N	Percent	N	Percent	N	Percent
Cost/day	80	100.0%	0	0.0%	80	100.0%

Descriptives

			Statistic	Std. Error
Cost/day	Mean		1051.9734640<br>44286400	87.537925892<br>911390
	95% Confidence Interval for Mean	Lower Bound	877.73358104<br>7667400	
		Upper Bound	1226.2133470<br>40905300	
	5% Trimmed Mean		924.67466133<br>0813800	
	Median		832.99019607<br>8431400	
	Variance		613031.078	
	Std. Deviation		782.96301162<br>3555400	
	Minimum		535.57142857<br>1428600	
	Maximum		6290.5575000<br>00000000	
	Range		5754.9860714<br>28571000	
	Interquartile Range		381.03763392<br>8571270	
	Skewness		4.656	.269
	Kurtosis		26.842	.532

Extreme Values

			Case Number	Value
Cost/day	Highest	1	702	6290.557500000000000
		2	852	3786.594411764706000
		3	1279	2810.101111111111000
		4	611	2399.095238095238000
		5	1065	2091.200000000000000
	Lowest	1	3	535.571428571428600
		2	1200	575.363636363636400
		3	640	591.200000000000000
		4	22	592.818181818181900
		5	449	615.000000000000000

Tests of Normality

	Kolmogorov-Smirnov[a]			Shapiro-Wilk		
	Statistic	df	Sig.	Statistic	df	Sig.
Cost/day	.262	80	<.001	.512	80	<.001

a. Lilliefors Significance Correction

Cost/day

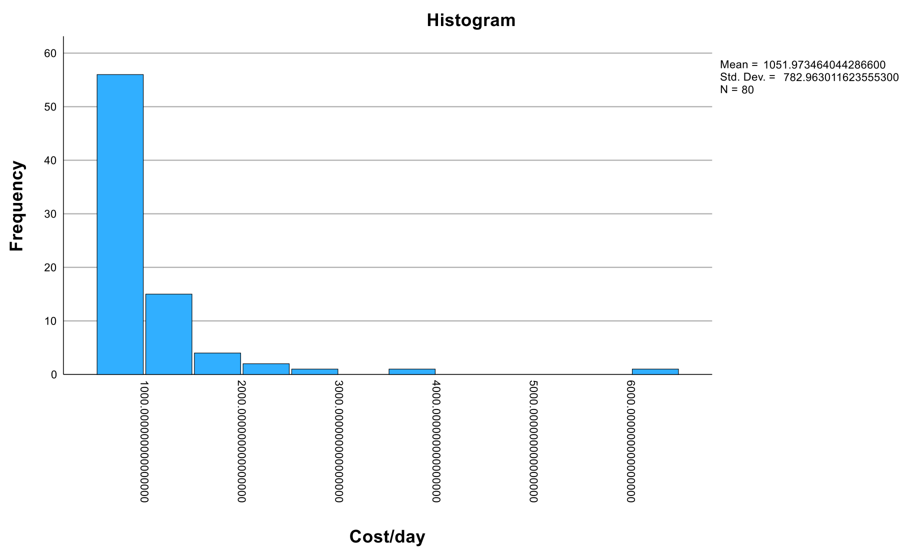


```
Cost/day Stem-and-Leaf Plot

 Frequency    Stem &  Leaf

     4.00        0 .  5555
    32.00        0 .  66666666666666666777777777777777
    20.00        0 .  88888888888999999999
     8.00        1 .  00000011
     6.00        1 .  222223
     3.00        1 .  455
     7.00 Extremes    (>=1657)

 Stem width:  1000.000
 Each leaf:        1 case(s)
```

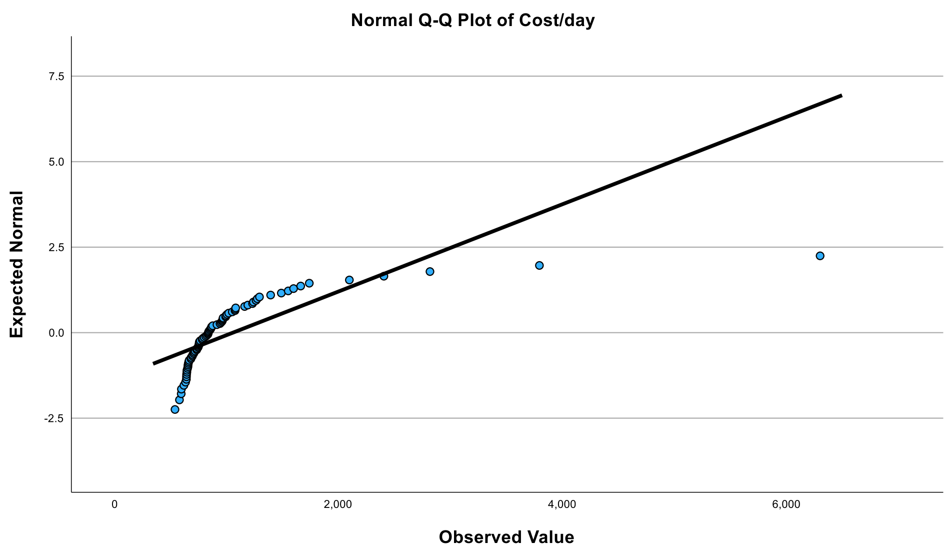
Normal Q-Q Plot of Cost/day
Expected Normal
7.5
5.0
2.5
0.0
-2.5
0
2,000
4,000
6,000
Observed Value

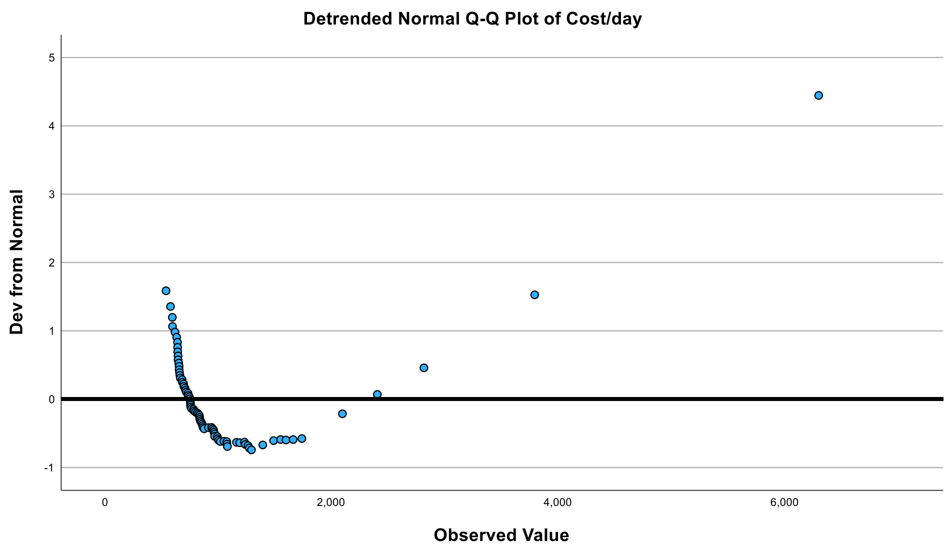
Detrended Normal Q-Q Plot of Cost/day
Dev from Normal
5
4
3
2
1
0
-1
0
2,000
4,000
6,000
Observed Value

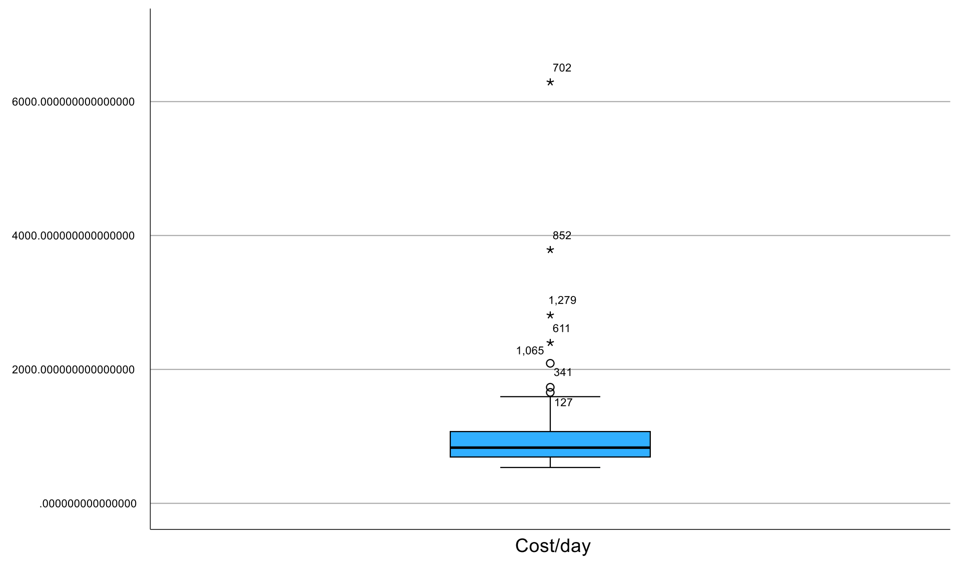

Explore DIABETES MELLITUS

Notes

Output Created		08-MAY-2023 00:13:21
Comments		
Input	Data	C:\Users\paho9\OneDrive\Documente\Doctorat - Stratificarea severității și predicția prognosticului în faza incipientă a Pancreatitei Acute\Registru Pancreatite Acute - BUC-API\Baza date nou\Articole\Articol cost\DB_corect_COST.sav
	Active Dataset	DataSet1
	Filter	Etiology = 7 (FILTER)
	Weight	<none>

	Split File	<none>
	N of Rows in Working Data File	62
Missing Value Handling	Definition of Missing	User-defined missing values for dependent variables are treated as missing.
	Cases Used	Statistics are based on cases with no missing values for any dependent variable or factor used.
Syntax		EXAMINE VARIABLES=Costday /PLOT BOXPLOT STEMLEAF HISTOGRAM NPPLOT /COMPARE GROUPS /STATISTICS DESCRIPTIVES EXTREME /CINTERVAL 95 /MISSING LISTWISE /NOTOTAL.
Resources	Processor Time	00:00:00.44
	Elapsed Time	00:00:00.62

Case Processing Summary

	Cases					
	Valid		Missing		Total	
	N	Percent	N	Percent	N	Percent
Cost/day	62	100.0%	0	0.0%	62	100.0%

Descriptives

			Statistic	Std. Error
Cost/day	Mean		1083.434184583267000	140.911630791324820
	95% Confidence Interval for Mean	Lower Bound	801.663964252886200	

			Statistic	Std. Error
		Upper Bound	1365.2044049 13648000	
	5% Trimmed Mean		894.81093047 2532500	
	Median		826.55681820 0000000	
	Variance		1231077.437	
	Std. Deviation		1109.5392903 90736800	
	Minimum		474.71428570 0000000	
	Maximum		8662.0000000 00000000	
	Range		8187.2857143 00000000	
	Interquartile Range		370.94607142 8571400	
	Skewness		5.753	.304
	Kurtosis		37.225	.599

Extreme Values

			Case Number	Value
Cost/day	Highest	1	1219	8662.0000000 00000000
		2	542	4022.3685714 28571600
		3	310	2806.0000000 00000000
		4	1211	1581.2500000 00000000
		5	758	1478.2927272 72727500
	Lowest	1	386	474.71428570 0000000
		2	565	503.49499999 9999950
		3	570	553.66666666 6666600

	4	85	621.000000000000000
	5	180	623.142857142857100

Tests of Normality

	Kolmogorov-Smirnov[a]			Shapiro-Wilk		
	Statistic	df	Sig.	Statistic	df	Sig.
Cost/day	.324	62	<.001	.371	62	<.001

a. Lilliefors Significance Correction

Cost/day

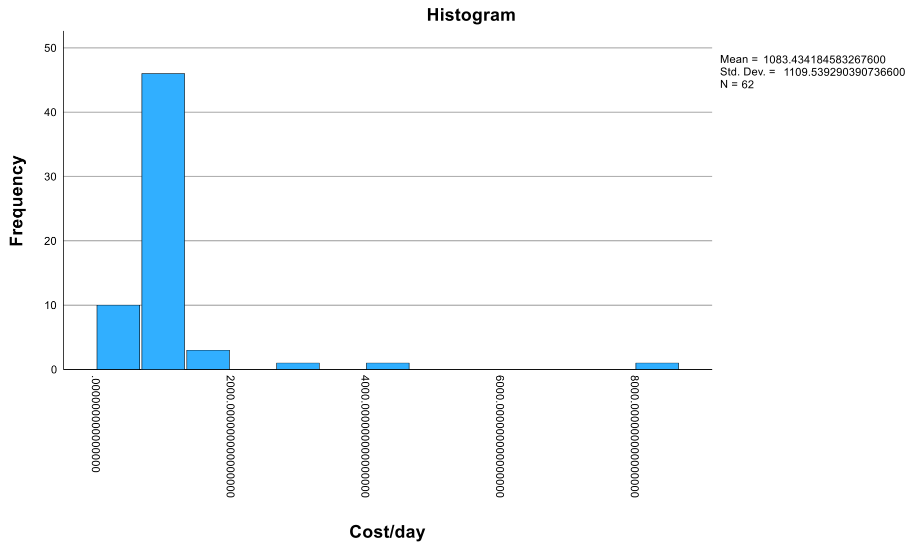


```
Cost/day Stem-and-Leaf Plot

 Frequency    Stem &  Leaf

     3.00        0 .  455
```

```
   24.00        0 .  666666666666677777777777
   17.00        0 .  88888888888889999
    7.00        1 .  0000011
    5.00        1 .  22223
    3.00        1 .  445
    3.00 Extremes    (>=2806)

Stem width:  1000.000
Each leaf:        1 case(s)
```

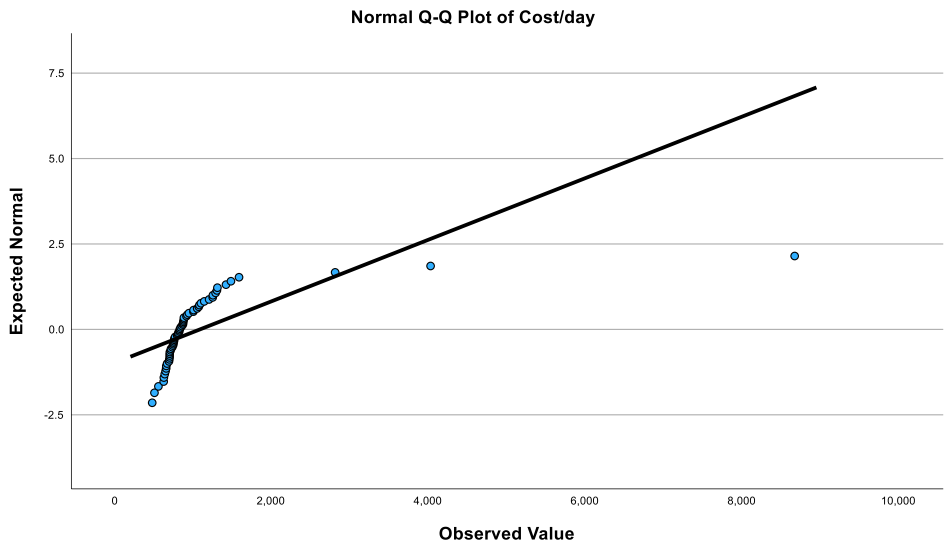

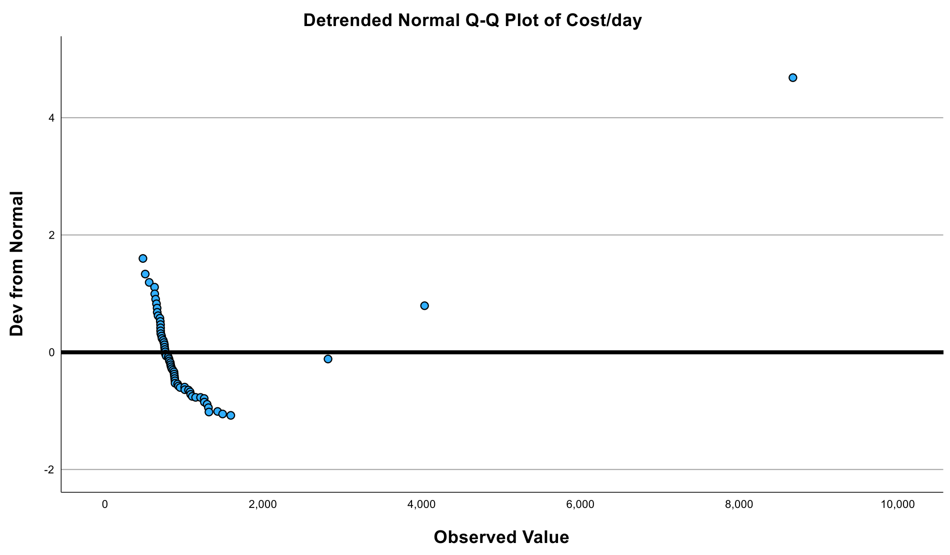

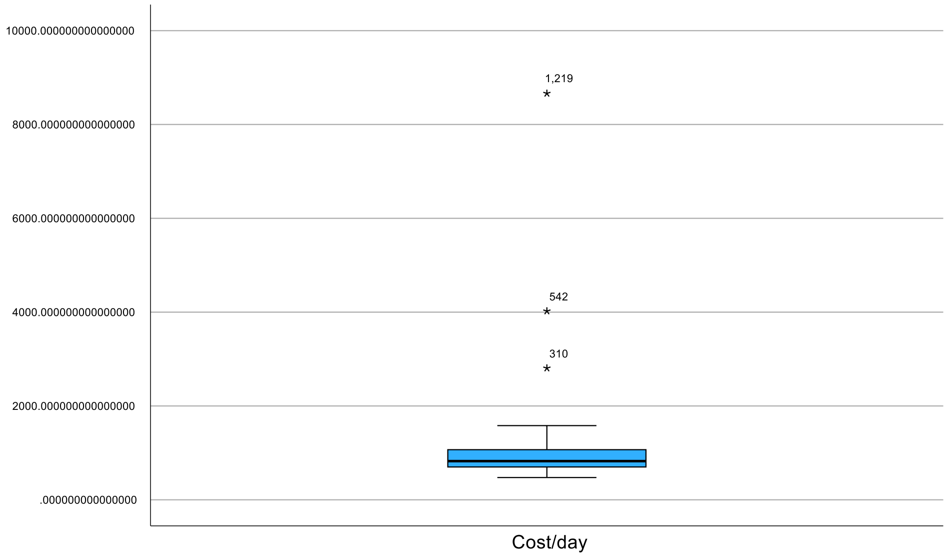

Nonparametric Tests ETIOLOGY

Notes

Output Created		08-MAY-2023 00:22:38
Comments		
Input	Data	C:\Users\paho9\OneDrive\Documente\Doctorat - Stratificarea severității și predicția prognosticului în faza incipientă a Pancreatitei Acute\Registru Pancreatite Acute - BUC-API\Baza date nou\Articole\Articol cost\DB_corect_COST.sav
	Active Dataset	DataSet1
	Filter	Etiology > 0 AND Etiology < 8 (FILTER)
	Weight	<none>
	Split File	<none>
	N of Rows in Working Data File	1168
Syntax		NPTESTS /INDEPENDENT TEST (Costday) GROUP (Etiology) KRUSKAL_WALLIS(COMPARE=PAIRWISE) /MISSING SCOPE=ANALYSIS USERMISSING=EXCLUDE /CRITERIA ALPHA=0.05 CILEVEL=95.
Resources	Processor Time	00:00:00.53
	Elapsed Time	00:00:00.79

Hypothesis Test Summary

Null Hypothesis	Test	Sig.[a,b]

1	The distribution of Cost/day is the same across categories of Etiology.	Independent-Samples Kruskal-Wallis Test	<.001

Hypothesis Test Summary

	Decision
1	Reject the null hypothesis.

a. The significance level is .050.

b. Asymptotic significance is displayed.

Independent-Samples Kruskal-Wallis Test

Cost/day across Etiology

Independent-Samples Kruskal-Wallis Test Summary

Total N	1168
Test Statistic	239.914[a]
Degree Of Freedom	3
Asymptotic Sig.(2-sided test)	<.001

a. The test statistic is adjusted for ties.

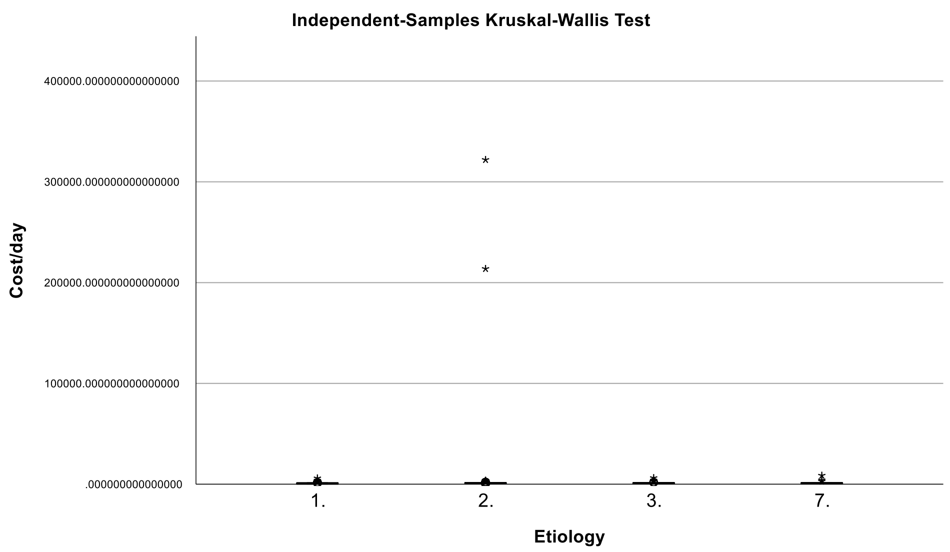

Pairwise Comparisons of Etiology

Sample 1-Sample 2	Test Statistic	Std. Error	Std. Test Statistic	Sig.	Adj. Sig.[a]
1 (Alcohol) -7 (Diabetes)	-108.145	45.335	-2.385	.017	.102
1 (Alcohol) -3 (Hypertrig.)	-119.377	40.526	-2.946	.003	.019
1 (Alcohol) -2 (Biliary)	-324.592	21.062	-15.411	<.001	.000
7 (Diabetes) -3 (Hypertrig.)	11.232	57.074	.197	.844	1.000
7 (Diabetes) -2 (Biliary)	216.448	45.373	4.770	<.001	.000
3 (Hypertrig.) -2 (Biliary)	205.216	40.569	5.058	<.001	.000

Each row tests the null hypothesis that the Sample 1 and Sample 2 distributions are the same.
Asymptotic significances (2-sided tests) are displayed. The significance level is .050.

a. Significance values have been adjusted by the Bonferroni correction for multiple tests.

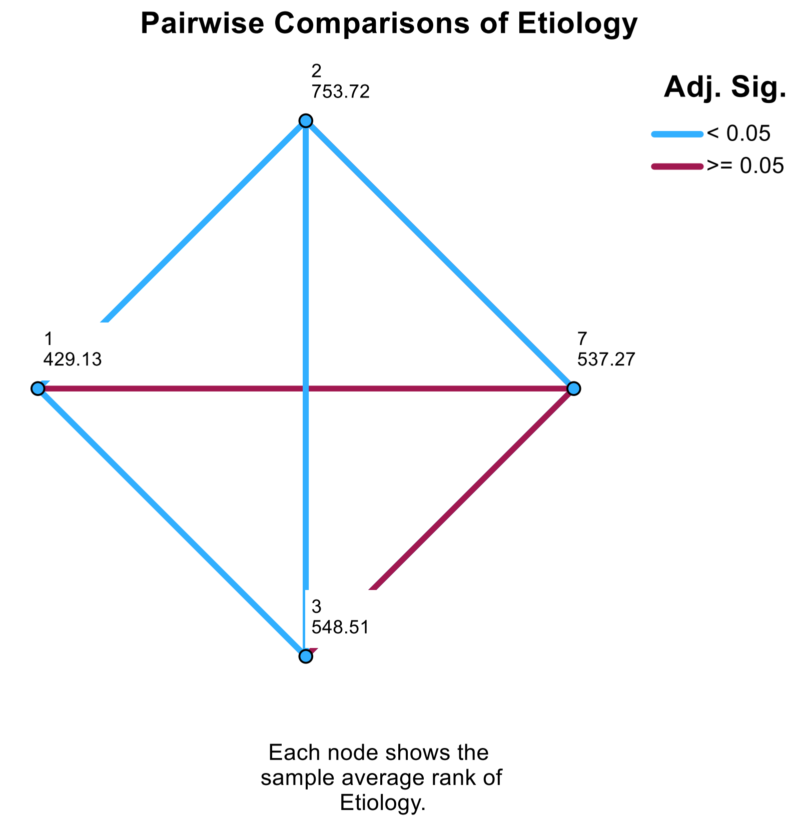

Pairwise Comparisons of Etiology
2
753.72
Adj. Sig.
< 0.05
>= 0.05
1
429.13
7
537.27
3
548.51
Each node shows the sample average rank of Etiology.

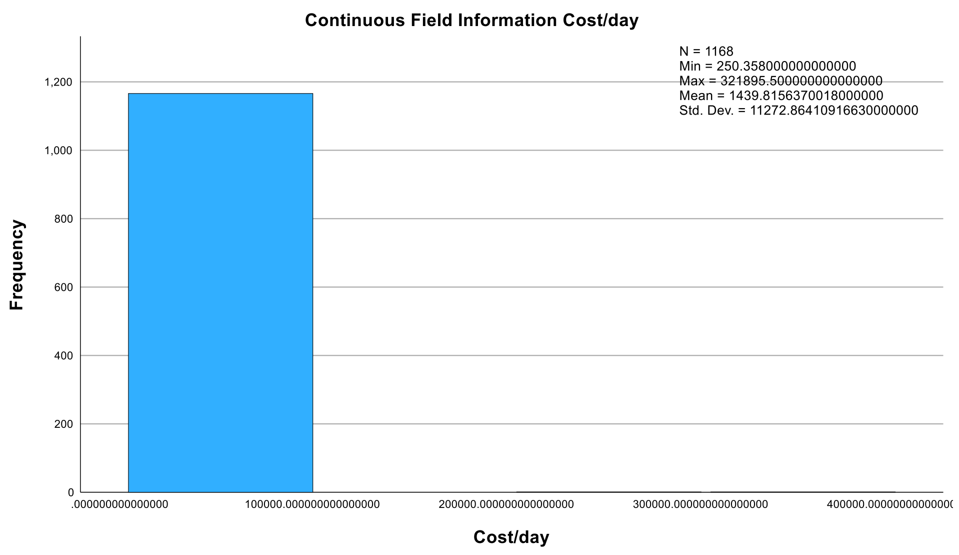

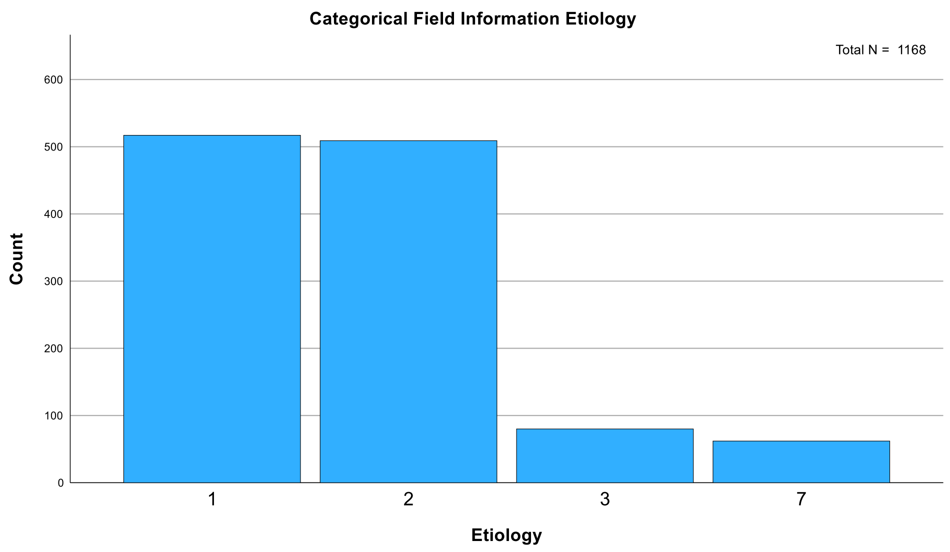

Explore INTERSTITIAL

Notes

Output Created		09-MAY-2023 10:05:41
Comments		
Input	Data	C:\Users\paho9\OneDrive\Documente\Doctorat - Stratificarea severității și predicția prognosticului în faza incipientă a Pancreatitei Acute\Registru Pancreatite Acute - BUC-API\Baza date nou\Articole\Articol cost\DB_corect_COST.sav
	Active Dataset	DataSet1
	Filter	RAC_morph = 1 (FILTER)
	Weight	<none>
	Split File	<none>
	N of Rows in Working Data File	575
Missing Value Handling	Definition of Missing	User-defined missing values for dependent variables are treated as missing.
	Cases Used	Statistics are based on cases with no missing values for any dependent variable or factor used.
Syntax		EXAMINE VARIABLES=Costday /PLOT BOXPLOT STEMLEAF HISTOGRAM NPPLOT /COMPARE GROUPS /STATISTICS DESCRIPTIVES EXTREME /CINTERVAL 95 /MISSING LISTWISE

		/NOTOTAL.
Resources	Processor Time	00:00:00.64
	Elapsed Time	00:00:01.11

Case Processing Summary

	Cases					
	Valid		Missing		Total	
	N	Percent	N	Percent	N	Percent
Cost/day	575	100.0%	0	0.0%	575	100.0%

Descriptives

			Statistic	Std. Error
Cost/day	Mean		942.981518044771200	18.959691640482696
	95% Confidence Interval for Mean	Lower Bound	905.742684526331600	
		Upper Bound	980.220351563210800	
	5% Trimmed Mean		899.840149333352000	
	Median		848.210000000000000	
	Variance		206695.197	
	Std. Deviation		454.637434208577800	
	Minimum		250.358000000000000	
	Maximum		7595.160000000000000	
	Range		7344.802000000000000	
	Interquartile Range		400.121000000000000	
	Skewness		6.789	.102
	Kurtosis		84.440	.203

Extreme Values

			Case Number	Value
Cost/day	Highest	1	1274	7595.160000000000000
		2	370	3663.000000000000000
		3	1144	3649.810000000000000
		4	131	2998.000000000000000
		5	602	2942.240000000000000
	Lowest	1	492	250.358000000000000
		2	257	277.444444444444460
		3	464	278.723333333333300
		4	476	328.333333333333300
		5	88	344.142857142857170

Tests of Normality

	Kolmogorov-Smirnov[a]			Shapiro-Wilk		
	Statistic	df	Sig.	Statistic	df	Sig.
Cost/day	.163	575	<.001	.610	575	<.001

a. Lilliefors Significance Correction

Cost/day

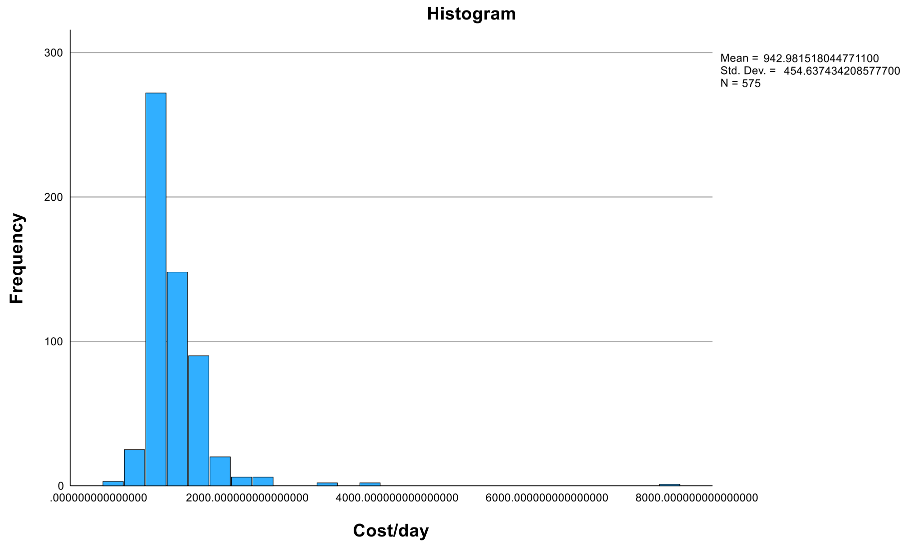


```
Cost/day Stem-and-Leaf Plot

 Frequency    Stem &  Leaf

     3.00        2 .  7&
     4.00        3 .  &&
     4.00        4 .  9&
    32.00        5 .  2455667889999&
   107.00        6 .  00011122223333334444445555555666666677777789999999
    94.00        7 .  000111122223333344444444455555666677788889999
    86.00        8 .  0000111122223333444445555556666777888999
    65.00        9 .  0000122233444556667778889999
    38.00       10 .  000111223456788999
    35.00       11 .  0001334555678899
    46.00       12 .  00112233456677888999
    23.00       13 .  0002246889&
    12.00       14 .  34459&
     7.00       15 .  37&
     2.00       16 .  &
    17.00 Extremes    (>=1727)

 Stem width:  100.0000
 Each leaf:        2 case(s)
```

& denotes fractional leaves.

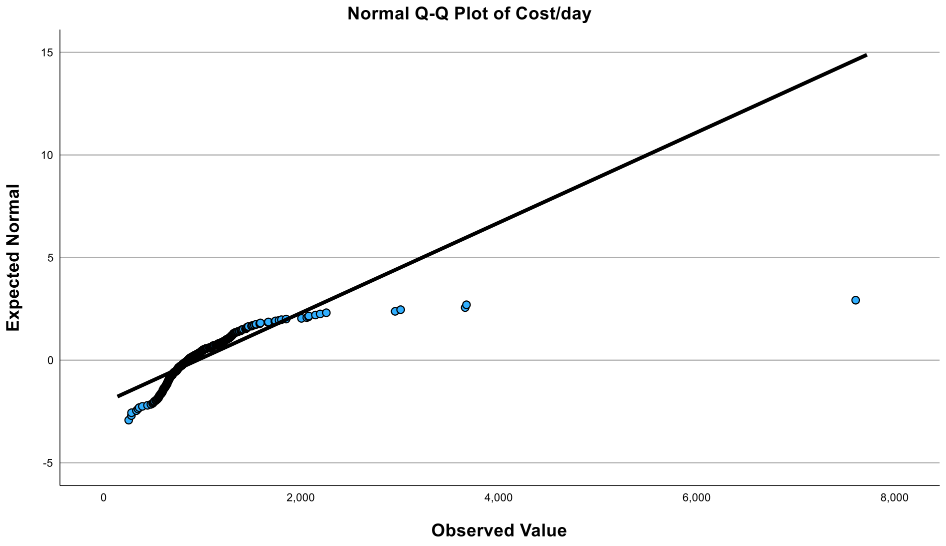

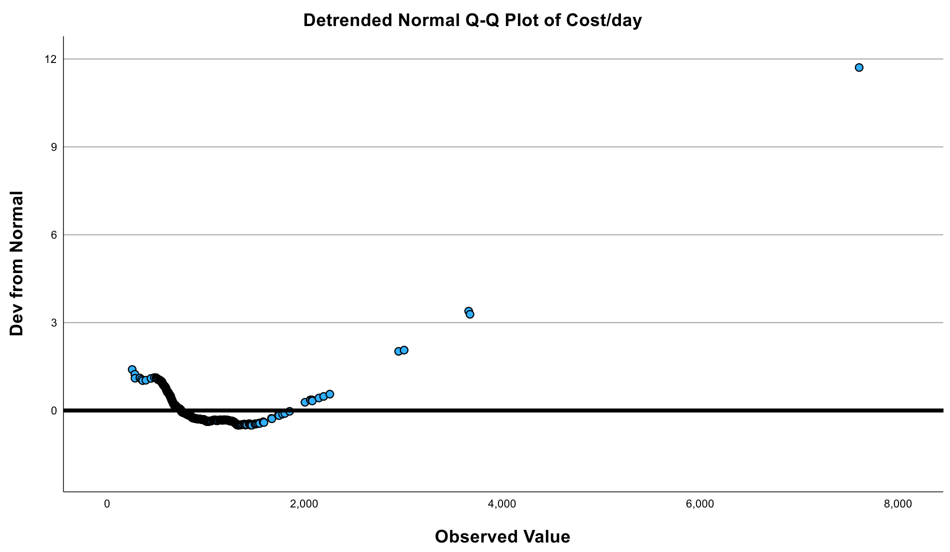

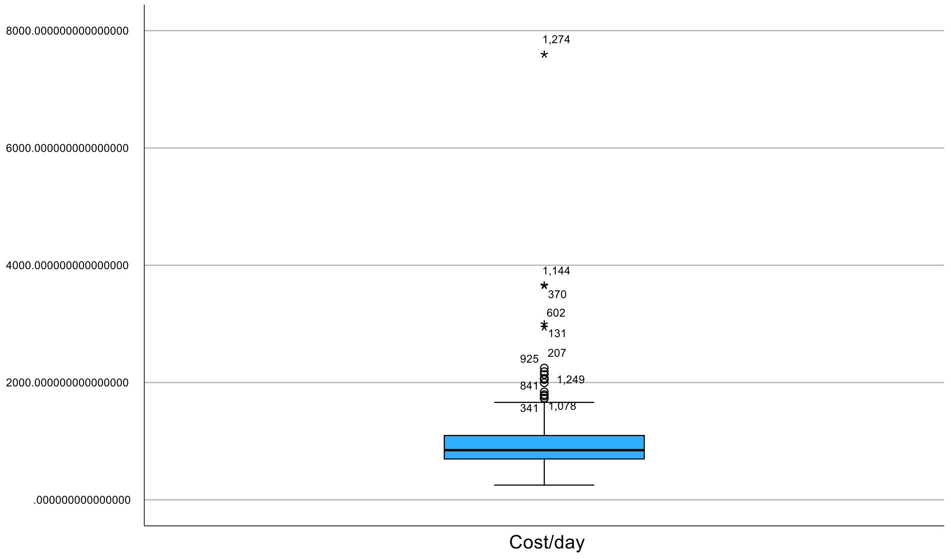

Explore APFC

Notes

Output Created		09-MAY-2023 10:06:28
Comments		
Input	Data	C:\Users\paho9\OneDrive\Documente\Doctorat - Stratificarea severității și predicția prognosticului în faza incipientă a Pancreatitei Acute\Registru Pancreatite Acute - BUC-API\Baza date nou\Articole\Articol cost\DB_corect_COST.sav
	Active Dataset	DataSet1
	Filter	RAC_morph = 3 (FILTER)
	Weight	<none>
	Split File	<none>
	N of Rows in Working Data File	114
Missing Value Handling	Definition of Missing	User-defined missing values for dependent variables are treated as missing.
	Cases Used	Statistics are based on cases with no missing values for any dependent variable or factor used.
Syntax		EXAMINE VARIABLES=Costday /PLOT BOXPLOT STEMLEAF HISTOGRAM NPPLOT /COMPARE GROUPS /STATISTICS DESCRIPTIVES EXTREME /CINTERVAL 95 /MISSING LISTWISE

		/NOTOTAL.
Resources	Processor Time	00:00:00.49
	Elapsed Time	00:00:00.69

Case Processing Summary

	Cases					
	Valid		Missing		Total	
	N	Percent	N	Percent	N	Percent
Cost/day	114	100.0%	0	0.0%	114	100.0%

Descriptives

				Statistic	Std. Error
Cost/day	Mean			1087.607295106553500	62.416817234860915
	95% Confidence Interval for Mean	Lower Bound		963.948322703699300	
		Upper Bound		1211.266267509407700	
	5% Trimmed Mean			1001.287931859783200	
	Median			968.169107142857100	
	Variance			444127.934	
	Std. Deviation			666.429241859346600	
	Minimum			325.400000000000000	
	Maximum			6255.410000000000000	
	Range			5930.010000000000000	
	Interquartile Range			523.269523809523900	
	Skewness			5.007	.226
	Kurtosis			34.096	.449

Extreme Values

			Case Number	Value
Cost/day	Highest	1	1247	6255.410000000000000
		2	867	3879.165000000000000
		3	1107	2734.160000000000000
		4	918	2292.116000000000000
		5	1092	1842.840000000000000
	Lowest	1	11	325.400000000000000
		2	556	558.086250000000000
		3	1457	565.016000000000000
		4	296	599.312500000000000
		5	228	599.833333333333400

Tests of Normality

	Kolmogorov-Smirnov[a]			Shapiro-Wilk		
	Statistic	df	Sig.	Statistic	df	Sig.
Cost/day	.206	114	<.001	.569	114	<.001

a. Lilliefors Significance Correction

Cost/day

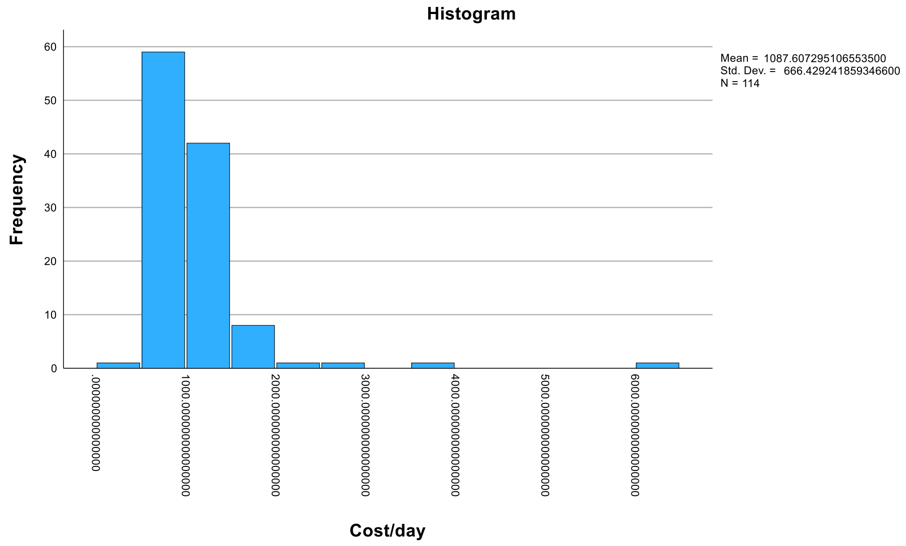


```
Cost/day Stem-and-Leaf Plot

 Frequency    Stem &  Leaf

     1.00        3 .  2
      .00        4 .
     4.00        5 .  5699
    15.00        6 .  122344457788899
    21.00        7 .  000012344455666667778
    13.00        8 .  0122334555579
     6.00        9 .  236678
    12.00       10 .  122344577789
    10.00       11 .  0123445677
     9.00       12 .  135677999
     5.00       13 .  13467
     6.00       14 .  006688
     4.00       15 .  1129
     3.00       16 .  127
      .00       17 .
     1.00       18 .  4
     4.00 Extremes    (>=2292)

 Stem width:  100.0000
```

```
Each leaf:       1 case(s)
```

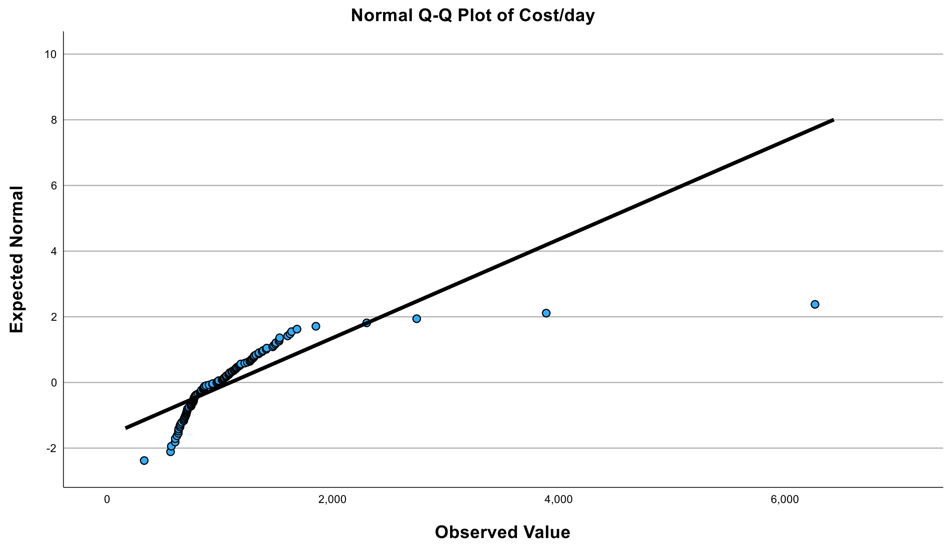

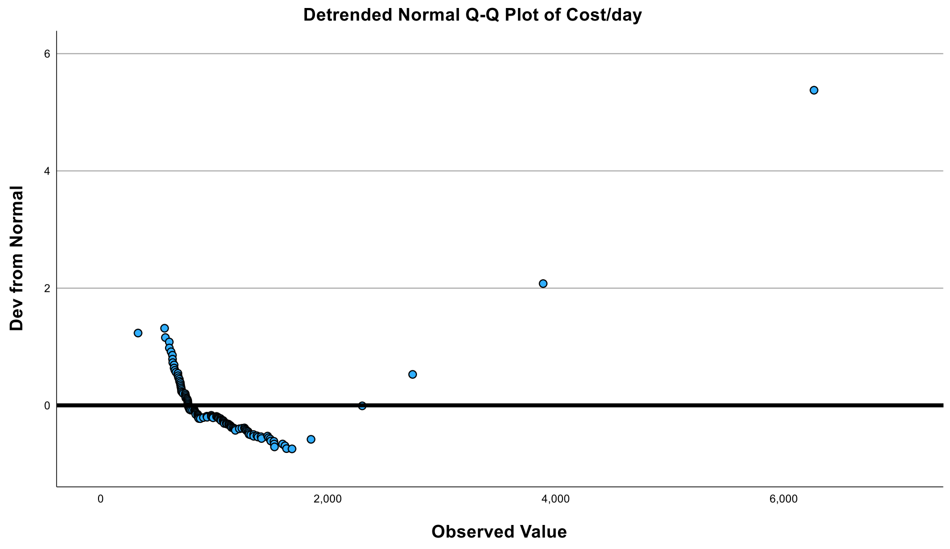

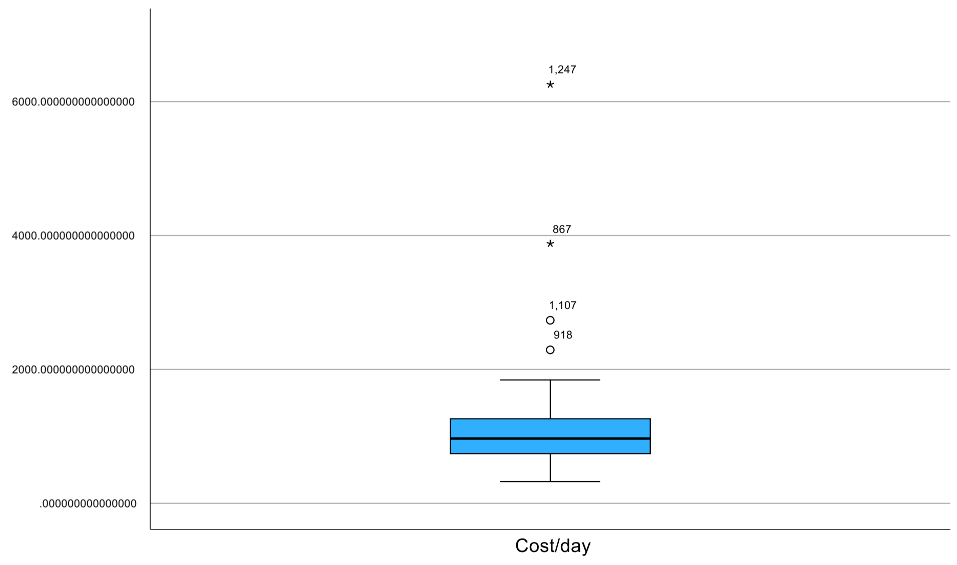

Explore PSEUDOCYST ! NORMAL DISTRIBUTION !

Notes

Output Created		09-MAY-2023 10:07:01
Comments		
Input	Data	C:\Users\paho9\OneDrive\Documente\Doctorat - Stratificarea severității și predicția prognosticului în faza incipientă a Pancreatitei Acute\Registru Pancreatite Acute - BUC-API\Baza date nou\Articole\Articol cost\DB_corect_COST.sav
	Active Dataset	DataSet1
	Filter	RAC_morph = 4 (FILTER)

	Weight	<none>
	Split File	<none>
	N of Rows in Working Data File	60
Missing Value Handling	Definition of Missing	User-defined missing values for dependent variables are treated as missing.
	Cases Used	Statistics are based on cases with no missing values for any dependent variable or factor used.
Syntax		EXAMINE VARIABLES=Costday /PLOT BOXPLOT STEMLEAF HISTOGRAM NPPLOT /COMPARE GROUPS /STATISTICS DESCRIPTIVES EXTREME /CINTERVAL 95 /MISSING LISTWISE /NOTOTAL.
Resources	Processor Time	00:00:00.53
	Elapsed Time	00:00:00.69

Case Processing Summary

	Cases					
	Valid		Missing		Total	
	N	Percent	N	Percent	N	Percent
Cost/day	60	100.0%	0	0.0%	60	100.0%

Descriptives

		Statistic	Std. Error
Cost/day	Mean	902.451832107963000	40.981696761220500

	95% Confidence Interval for Mean	Lower Bound	820.447646302560800	
		Upper Bound	984.456017913365300	
	5% Trimmed Mean		877.436918323730300	
	Median		833.111111111111100	
	Variance		100769.968	
	Std. Deviation		317.442858111058850	
	Minimum		442.714285714285700	
	Maximum		2194.900000000000000	
	Range		1752.185714285714500	
	Interquartile Range		357.528749999999830	
	Skewness		1.483	.309
	Kurtosis		3.625	.608

Extreme Values

			Case Number	Value
Cost/day	Highest	1	1071	2194.900000000000000
		2	460	1605.231666666666600
		3	1317	1545.875000000000000
		4	1186	1437.706666666666700
		5	302	1416.000000000000000
	Lowest	1	635	442.714285714285700
		2	348	467.680000000000000

	3	526	509.115384615384640
	4	293	539.285714285714300
	5	239	555.000000000000000

Tests of Normality

	Kolmogorov-Smirnov[a]			Shapiro-Wilk		
	Statistic	df	Sig.	Statistic	df	Sig.
Cost/day	.102	60	.197	.902	60	<.001

a. Lilliefors Significance Correction

Cost/day

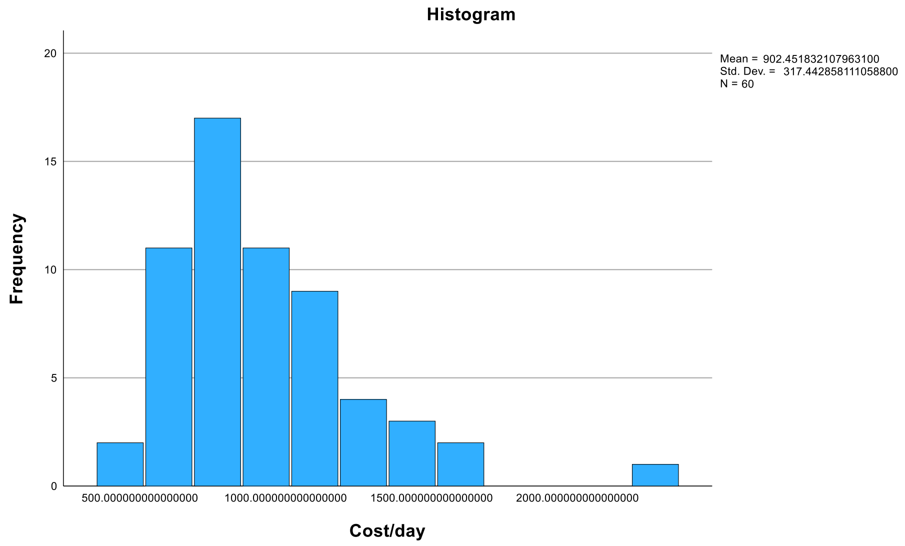


```
Cost/day Stem-and-Leaf Plot

 Frequency    Stem &  Leaf
```

```
    8.00        0 .  44555555
   19.00        0 .  6666666667777777777
   14.00        0 .  88888889999999
   12.00        1 .  000000001111
    2.00        1 .  23
    3.00        1 .  445
    2.00 Extremes    (>=1605)

Stem width:  1000.000
Each leaf:        1 case(s)
```

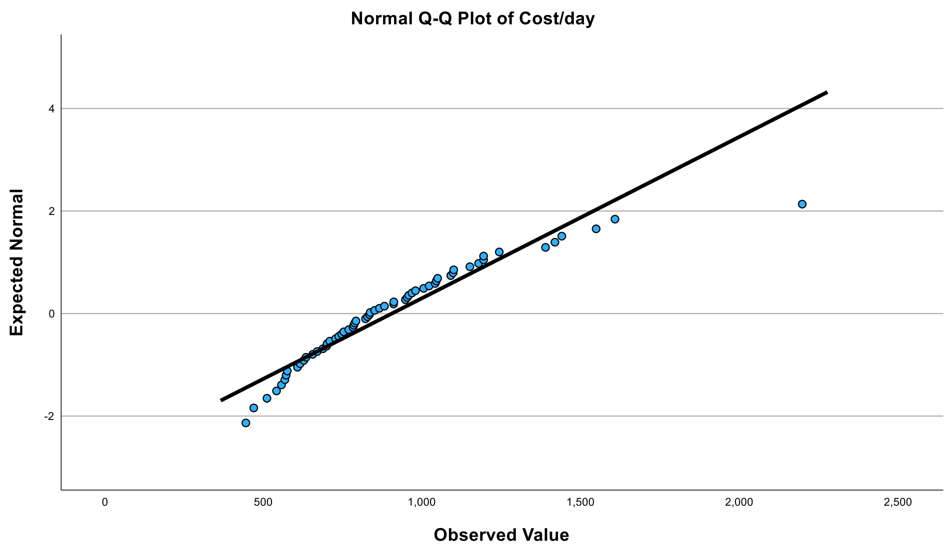

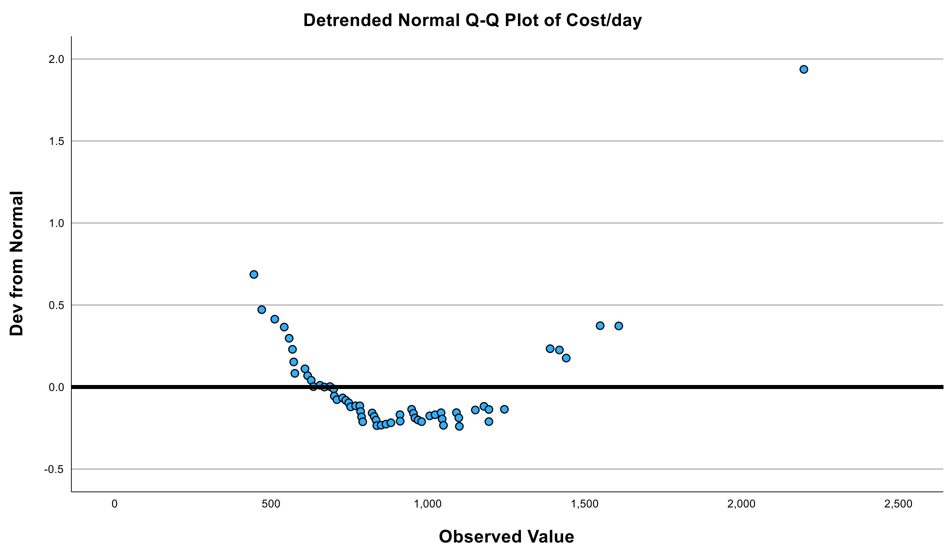

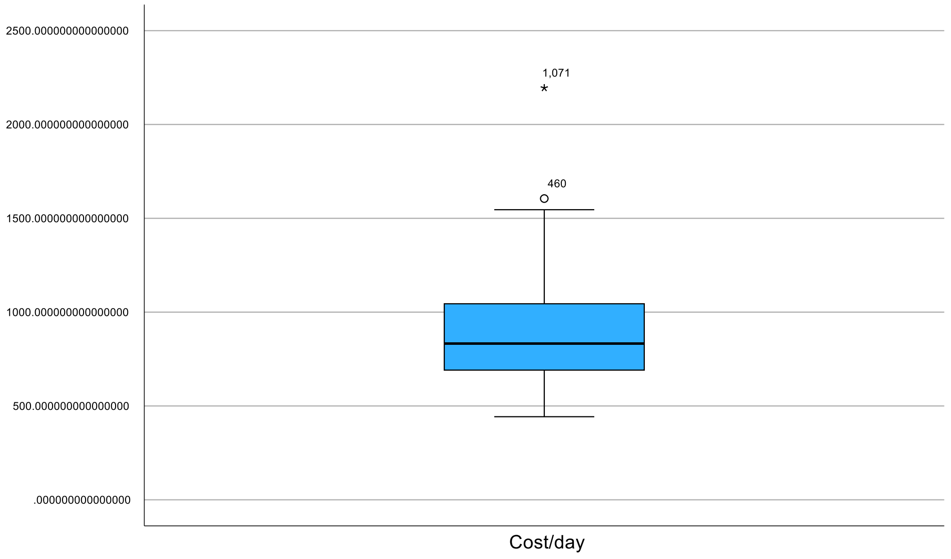

Explore ANC ! NORMAL DISTRIBUTION !

Notes

Output Created		09-MAY-2023 10:08:04
Comments		
Input	Data	C:\Users\paho9\OneDrive\Documente\Doctorat - Stratificarea severității și predicția prognosticului în faza incipientă a Pancreatitei Acute\Registru Pancreatite Acute - BUC-API\Baza date nou\Articole\Articol cost\DB_corect_COST.sav
	Active Dataset	DataSet1
	Filter	RAC_morph = 5 (FILTER)
	Weight	<none>
	Split File	<none>
	N of Rows in Working Data File	63
Missing Value Handling	Definition of Missing	User-defined missing values for dependent variables are treated as missing.
	Cases Used	Statistics are based on cases with no missing values for any dependent variable or factor used.
Syntax		EXAMINE VARIABLES=Costday /PLOT BOXPLOT STEMLEAF HISTOGRAM NPPLOT /COMPARE GROUPS /STATISTICS DESCRIPTIVES EXTREME /CINTERVAL 95 /MISSING LISTWISE

		/NOTOTAL.
Resources	Processor Time	00:00:00.34
	Elapsed Time	00:00:00.65

Case Processing Summary

	Cases					
	Valid		Missing		Total	
	N	Percent	N	Percent	N	Percent
Cost/day	63	100.0%	0	0.0%	63	100.0%

Descriptives

			Statistic	Std. Error
Cost/day	Mean		1063.092854105450700	43.340379281501235
	95% Confidence Interval for Mean	Lower Bound	976.456670384322300	
		Upper Bound	1149.729037826578900	
	5% Trimmed Mean		1030.537826607038600	
	Median		1003.250000000000000	
	Variance		118338.474	
	Std. Deviation		344.003595918205500	
	Minimum		575.363636363636400	
	Maximum		2298.664444444444600	
	Range		1723.300808080808000	
	Interquartile Range		403.820666666666600	
	Skewness		1.488	.302
	Kurtosis		3.385	.595

Extreme Values

			Case Number	Value
Cost/day	Highest	1	704	2298.6644444 44444600
		2	1134	2217.5255263 15789600
		3	7	1928.0000000 00000000
		4	758	1478.2927272 72727500
		5	1120	1475.0860000 00000000
	Lowest	1	1200	575.36363636 3636400
		2	654	591.12466666 6666700
		3	1461	616.46500000 0000000
		4	1322	629.78571428 5714300
		5	437	633.50000000 0000000

Tests of Normality

	Kolmogorov-Smirnov[a]			Shapiro-Wilk		
	Statistic	df	Sig.	Statistic	df	Sig.
Cost/day	.106	63	.079	.889	63	<.001

a. Lilliefors Significance Correction

Cost/day

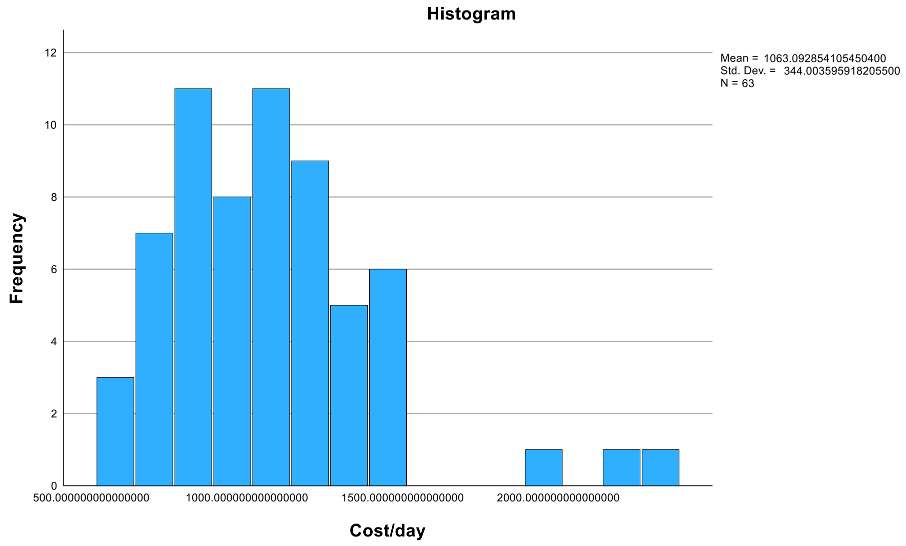


```
Cost/day Stem-and-Leaf Plot

 Frequency    Stem &  Leaf

     2.00        5 .  79
     5.00        6 .  12377
     4.00        7 .  1248
    12.00        8 .  001233666799
     6.00        9 .  233469
     9.00       10 .  000022369
     9.00       11 .  023346778
     4.00       12 .  3477
     5.00       13 .  26789
     4.00       14 .  0577
     3.00 Extremes    (>=1928)

 Stem width:  100.0000
 Each leaf:        1 case(s)
```

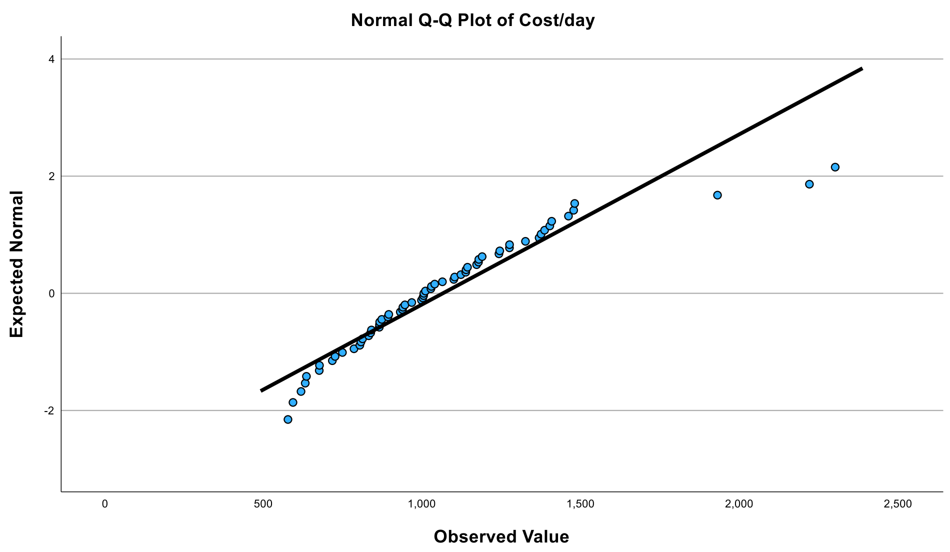

Normal Q-Q Plot of Cost/day
Expected Normal
4
2
0
-2
0
500
1,000
1,500
2,000
2,500
Observed Value

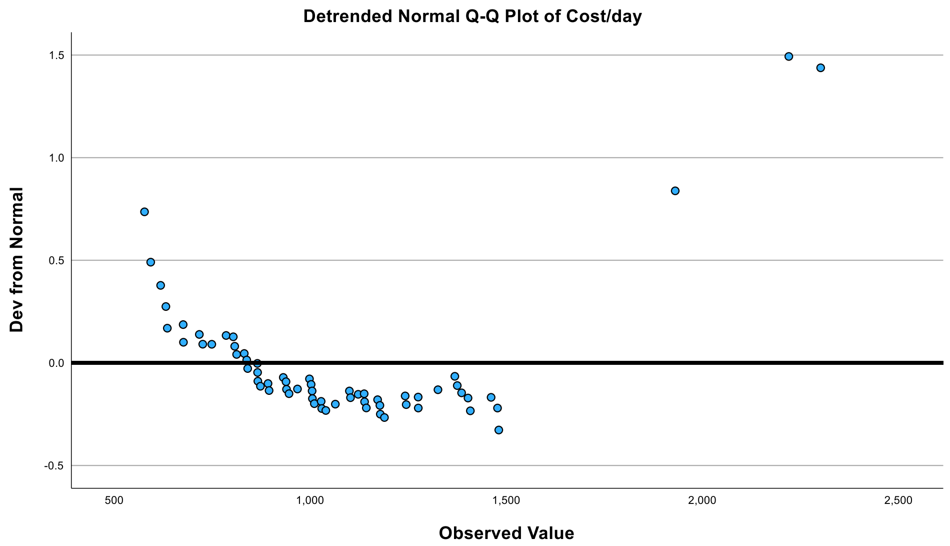

Detrended Normal Q-Q Plot of Cost/day
Dev from Normal
1.5
1.0
0.5
0.0
-0.5
500
1,000
1,500
2,000
2,500
Observed Value

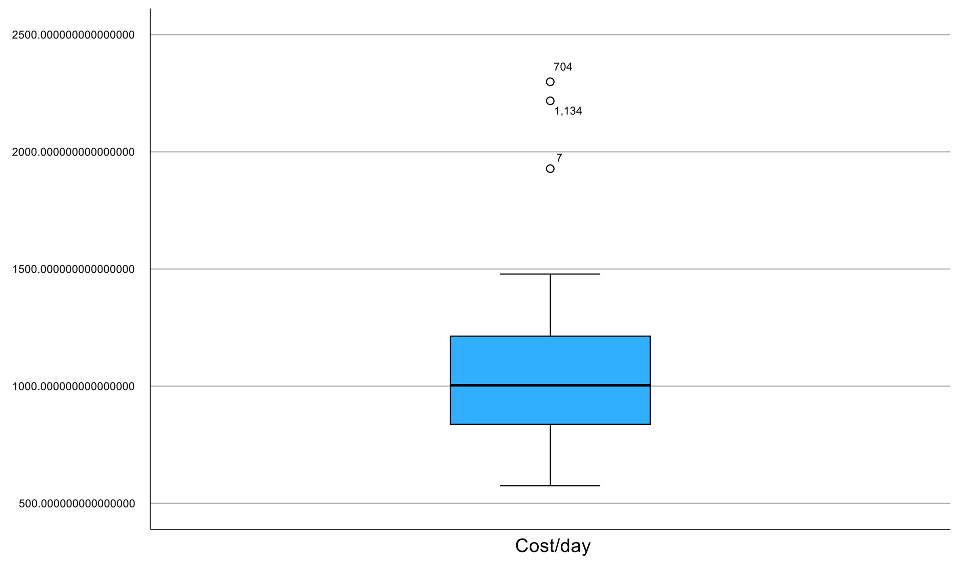

Explore NORMAL PANCREAS

Notes

Output Created		09-MAY-2023 10:12:52
Comments		
Input	Data	C:\Users\paho9\OneDrive\Documente\Doctorat - Stratificarea severității și predicția prognosticului în faza incipientă a Pancreatitei Acute\Registru Pancreatite Acute - BUC-API\Baza date nou\Articole\Articol cost\DB_corect_COST.sav
	Active Dataset	DataSet1
	Filter	RAC_morph = 7 (FILTER)

	Weight	<none>
	Split File	<none>
	N of Rows in Working Data File	210
Missing Value Handling	Definition of Missing	User-defined missing values for dependent variables are treated as missing.
	Cases Used	Statistics are based on cases with no missing values for any dependent variable or factor used.
Syntax		EXAMINE VARIABLES=Costday /PLOT BOXPLOT STEMLEAF HISTOGRAM NPPLOT /COMPARE GROUPS /STATISTICS DESCRIPTIVES EXTREME /CINTERVAL 95 /MISSING LISTWISE /NOTOTAL.
Resources	Processor Time	00:00:00.39
	Elapsed Time	00:00:00.64

Case Processing Summary

	Cases					
	Valid		Missing		Total	
	N	Percent	N	Percent	N	Percent
Cost/day	210	100.0%	0	0.0%	210	100.0%

Descriptives

		Statistic	Std. Error
Cost/day	Mean	2511.4666660 43804300	1528.3009219 17231000

	95% Confidence Interval for Mean	Lower Bound	-501.394382487234450	
		Upper Bound	5524.327714574844000	
	5% Trimmed Mean		964.222347833732200	
	Median		963.655000000000000	
	Variance		490497778.666	
	Std. Deviation		9223.372036854777000	
	Minimum		371.770000000000000	
	Maximum		9223.372036854777000	
	Range		9223.372036854777000	
	Interquartile Range		392.753076923076900	
	Skewness		14.487	.168
	Kurtosis		209.918	.334

Extreme Values

			Case Number	Value
Cost/day	Highest	1	1049	9223.372036854777000
		2	311	2459.000000000000000
		3	956	2064.480000000000000
		4	1208	1998.500000000000000
		5	1005	1763.466666666666700
	Lowest	1	802	371.770000000000000
		2	507	544.785000000000000

	3	622	548.857142857142900
	4	326	554.111111111111100
	5	424	563.750000000000000

Tests of Normality

	Kolmogorov-Smirnov[a]			Shapiro-Wilk		
	Statistic	df	Sig.	Statistic	df	Sig.
Cost/day	.499	210	<.001	.048	210	<.001

a. Lilliefors Significance Correction

Cost/day

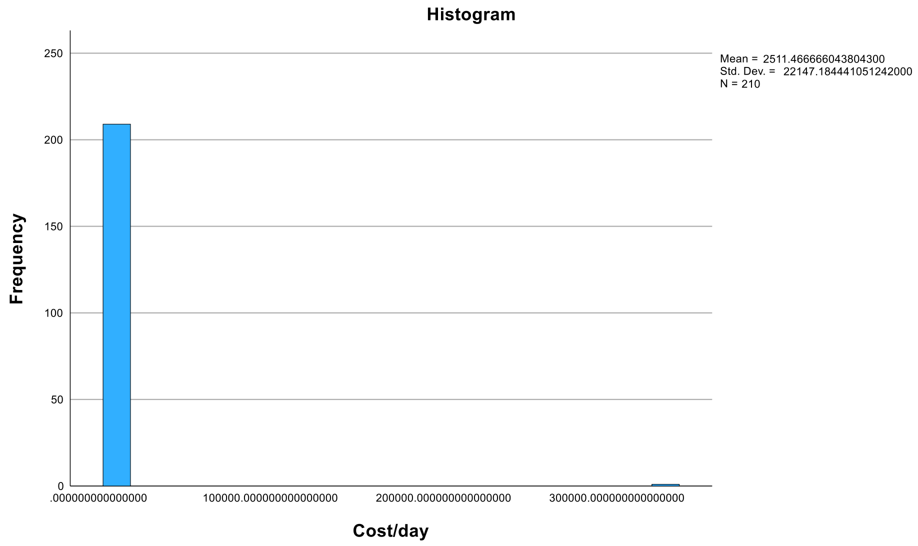


```
Cost/day Stem-and-Leaf Plot

 Frequency    Stem &  Leaf
```

```
    1.00        3 .  7
     .00        4 .
    9.00        5 .  445667789
   30.00        6 .  001111122233334445555566777889
   29.00        7 .  01112222333445555566777888 8999
   18.00        8 .  111223444567778889
   32.00        9 .  01123333333444555666677778888999
   29.00       10 .  01111122222223444567777888999
   17.00       11 .  00012222344567789
   16.00       12 .  0011244445677778
   11.00       13 .  00012233567
    6.00       14 .  113467
    1.00       15 .  9
    6.00       16 .  477778
    5.00 Extremes    (>=1763)

Stem width:  100.0000
Each leaf:        1 case(s)
```

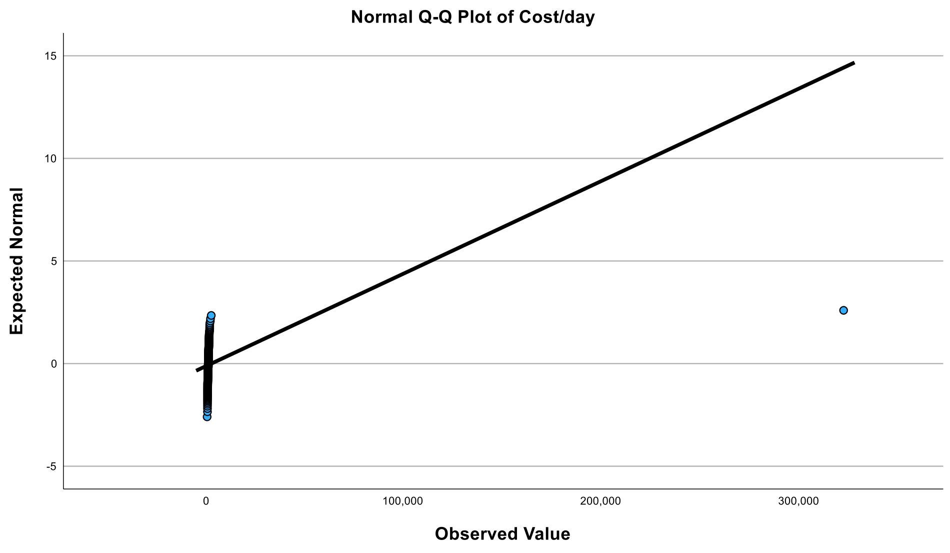

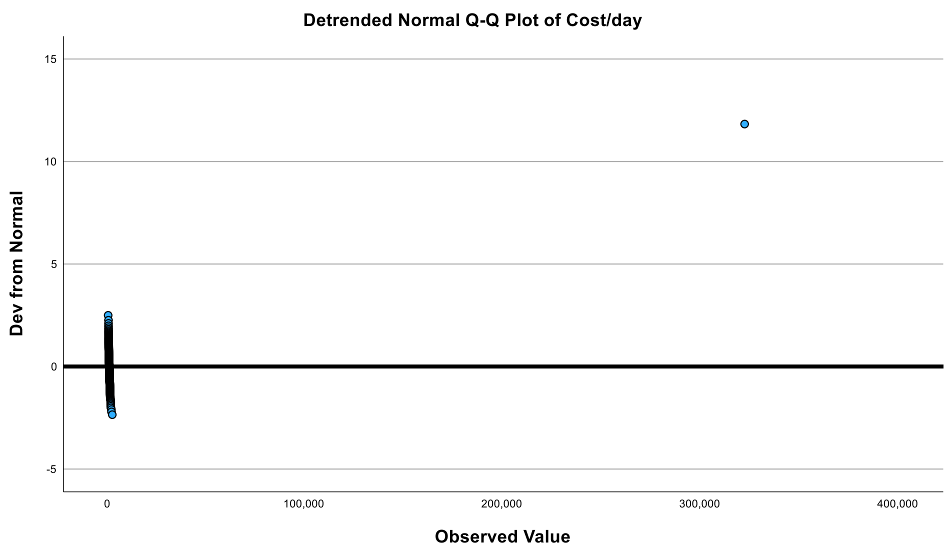

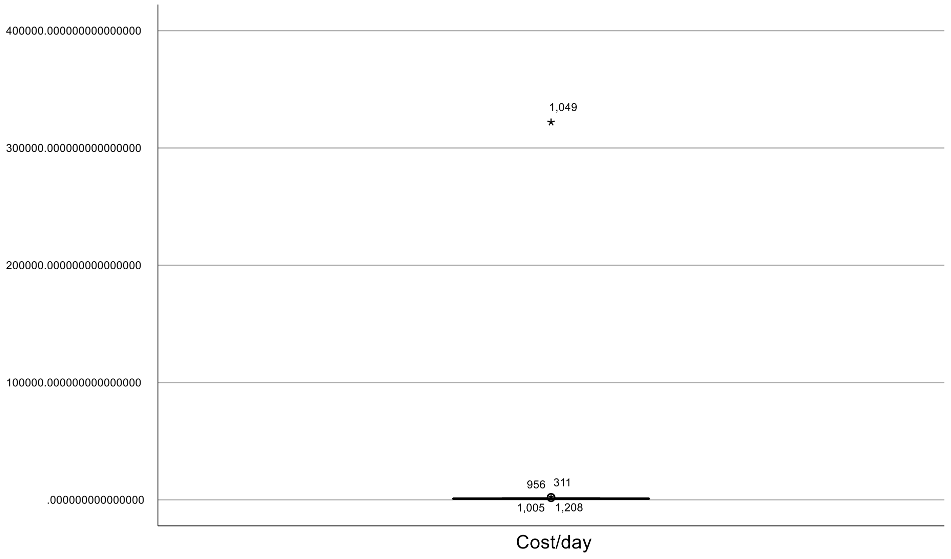

Nonparametric Tests MORPHOLOGY

Notes

Output Created		09-MAY-2023 10:43:50
Comments		
Input	Data	C:\Users\paho9\OneDrive\Documente\Doctorat - Stratificarea severității și predicția prognosticului în faza incipientă a Pancreatitei Acute\Registru Pancreatite Acute - BUC-API\Baza date nou\Articole\Articol cost\DB_corect_COST.sav
	Active Dataset	DataSet1
	Filter	RAC_morph > 0 AND RAC_morph < 10 (FILTER)
	Weight	<none>
	Split File	<none>
	N of Rows in Working Data File	1022
Syntax		NPTESTS /INDEPENDENT TEST (Costday) GROUP (RAC_morph) KRUSKAL_WALLIS(COMPARE=PAIRWISE) /MISSING SCOPE=ANALYSIS USERMISSING=EXCLUDE /CRITERIA ALPHA=0.05 CILEVEL=95.
Resources	Processor Time	00:00:00.59
	Elapsed Time	00:00:00.98

Hypothesis Test Summary

Null Hypothesis	Test	Sig.[a,b]

1	The distribution of Cost/day is the same across categories of RAC_morph.	Independent-Samples Kruskal-Wallis Test	<.001

Hypothesis Test Summary

	Decision
1	Reject the null hypothesis.

a. The significance level is .050.

b. Asymptotic significance is displayed.

Independent-Samples Kruskal-Wallis Test

Cost/day across RAC_morph

Independent-Samples Kruskal-Wallis Test Summary

Total N	1022
Test Statistic	24.346[a]
Degree Of Freedom	4
Asymptotic Sig.(2-sided test)	<.001

a. The test statistic is adjusted for ties.

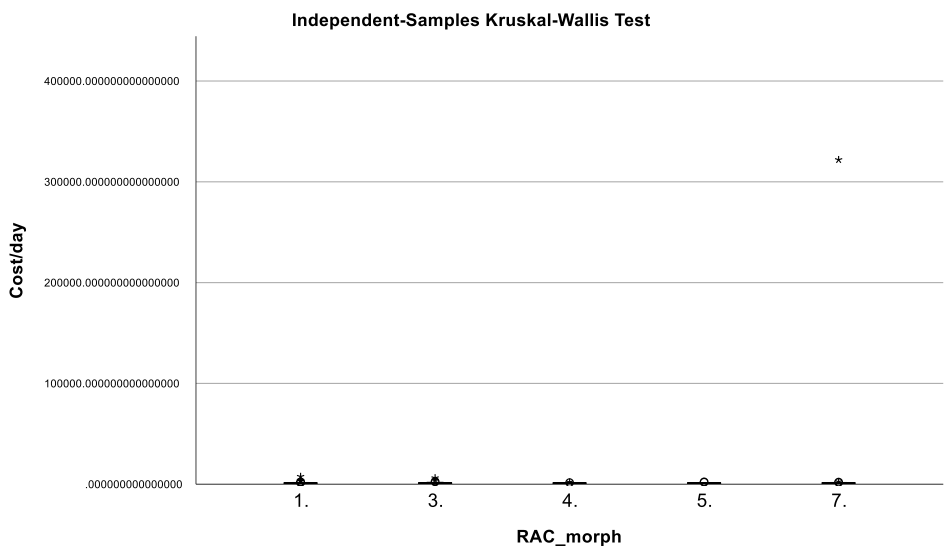

Pairwise Comparisons of RAC_morph

Sample 1-Sample 2	Test Statistic	Std. Error	Std. Test Statistic	Sig.	Adj. Sig.[a]
4 (Pseudocyst) -1 (Intestitial)	22.994	40.045	.574	.566	1.000
4 (Pseudocyst) -7 (Normal)	-92.208	43.209	-2.134	.033	.328
4 (Pseudocyst) -3 (APFC)	110.186	47.078	2.341	.019	.193
4 (Pseudocyst) – 5 (ANC)	-161.419	53.245	-3.032	.002	.024
1 (Interst.) -7 (Normal)	-69.215	23.799	-2.908	.004	.036
1 (Interst.) -3 (APFC)	-87.193	30.262	-2.881	.004	.040
1 (Interst.) -5 (ANC)	-138.425	39.172	-3.534	<.001	.004
7 (Normal) -3 (APFC)	17.978	34.339	.524	.601	1.000
7 (Normal) -5 (ANC)	69.210	42.401	1.632	.103	1.000
3 (APFC) -5 (ANC)	-51.232	46.338	-1.106	.269	1.000

Each row tests the null hypothesis that the Sample 1 and Sample 2 distributions are the same.
Asymptotic significances (2-sided tests) are displayed. The significance level is .050.

a. Significance values have been adjusted by the Bonferroni correction for multiple tests.

Pairwise Comparisons of RAC_morph

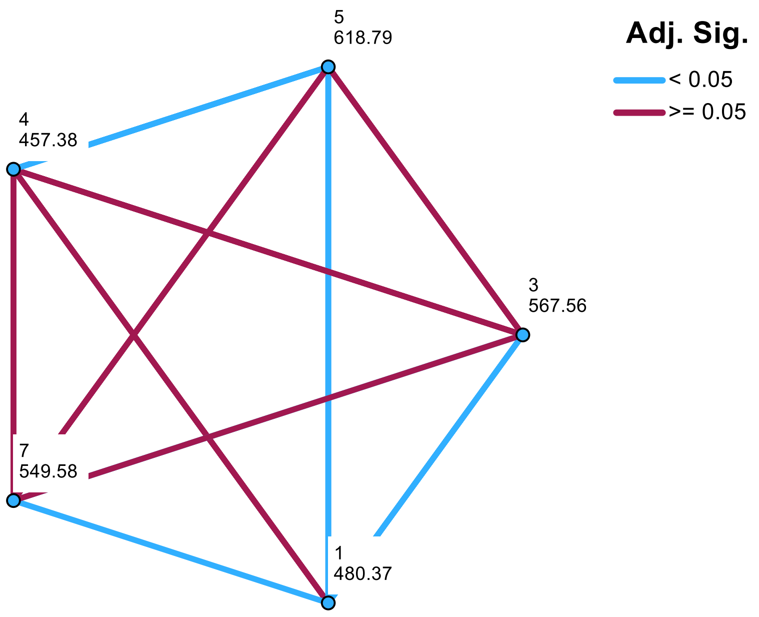

Each node shows the sample average rank of RAC_morph.

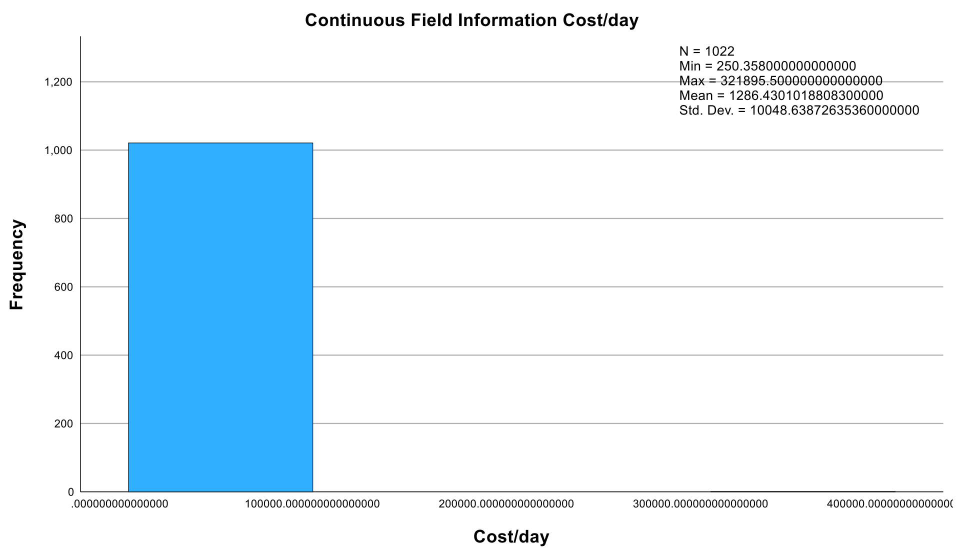

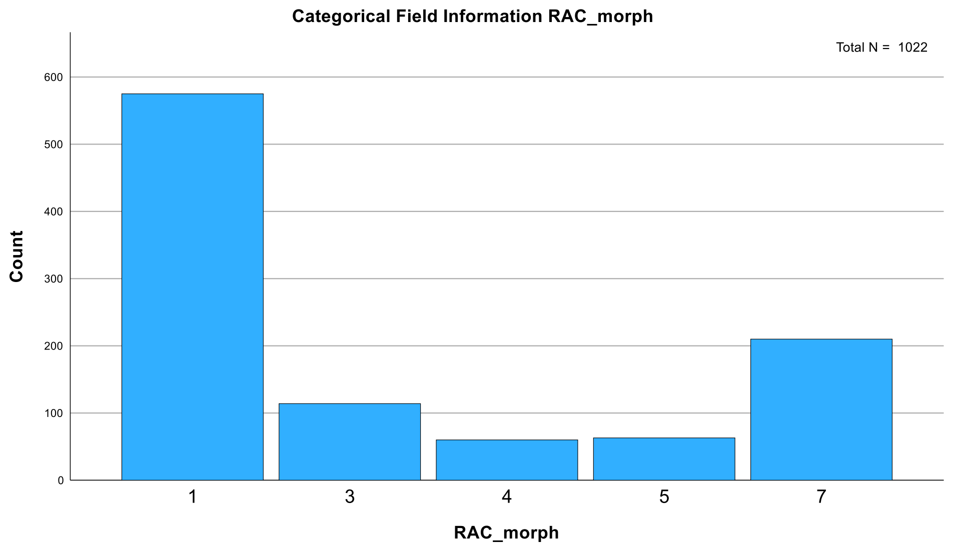

Explore SEVERITY - MILD

Notes

Output Created		09-MAY-2023 11:02:24
Comments		
Input	Data	C:\Users\paho9\OneDrive\Documente\Doctorat - Stratificarea severității și predicția prognosticului în faza incipientă a Pancreatitei Acute\Registru Pancreatite Acute - BUC-API\Baza date nou\Articole\Articol cost\DB_corect_COST.sav
	Active Dataset	DataSet1
	Filter	RAC_Severity = 1 (FILTER)
	Weight	<none>
	Split File	<none>
	N of Rows in Working Data File	758
Missing Value Handling	Definition of Missing	User-defined missing values for dependent variables are treated as missing.
	Cases Used	Statistics are based on cases with no missing values for any dependent variable or factor used.
Syntax		EXAMINE VARIABLES=Costday /PLOT BOXPLOT STEMLEAF HISTOGRAM NPPLOT /COMPARE GROUPS /STATISTICS DESCRIPTIVES EXTREME /CINTERVAL 95 /MISSING LISTWISE

		/NOTOTAL.
Resources	Processor Time	00:00:00.52
	Elapsed Time	00:00:00.82

Case Processing Summary

	Cases					
	Valid		Missing		Total	
	N	Percent	N	Percent	N	Percent
Cost/day	758	100.0%	0	0.0%	758	100.0%

Descriptives

			Statistic	Std. Error
Cost/day	Mean		1498.5806196 30416300	420.66119385 7652200
	95% Confidence Interval for Mean	Lower Bound	672.77949606 0185700	
		Upper Bound	2324.3817432 00647000	
	5% Trimmed Mean		882.03301564 3138000	
	Median		851.56750000 0000000	
	Variance		134132526.73 3	
	Std. Deviation		9223.3720368 54777000	
	Minimum		250.35800000 0000000	
	Maximum		9223.3720368 54777000	
	Range		9223.3720368 54777000	
	Interquartile Range		363.08083333 3333430	
	Skewness		19.494	.089
	Kurtosis		380.448	.177

Extreme Values

			Case Number	Value
Cost/day	Highest	1	1125	9223.372036854777000
		2	1018	9223.372036854777000
		3	1109	4713.045000000000000
		4	1107	2734.160000000000000
		5	975	2503.500000000000000
	Lowest	1	492	250.358000000000000
		2	206	252.000000000000000
		3	257	277.444444444444460
		4	464	278.723333333333300
		5	232	291.500000000000000

Tests of Normality

	Kolmogorov-Smirnov[a]			Shapiro-Wilk		
	Statistic	df	Sig.	Statistic	df	Sig.
Cost/day	.478	758	<.001	.035	758	<.001

a. Lilliefors Significance Correction

Cost/day

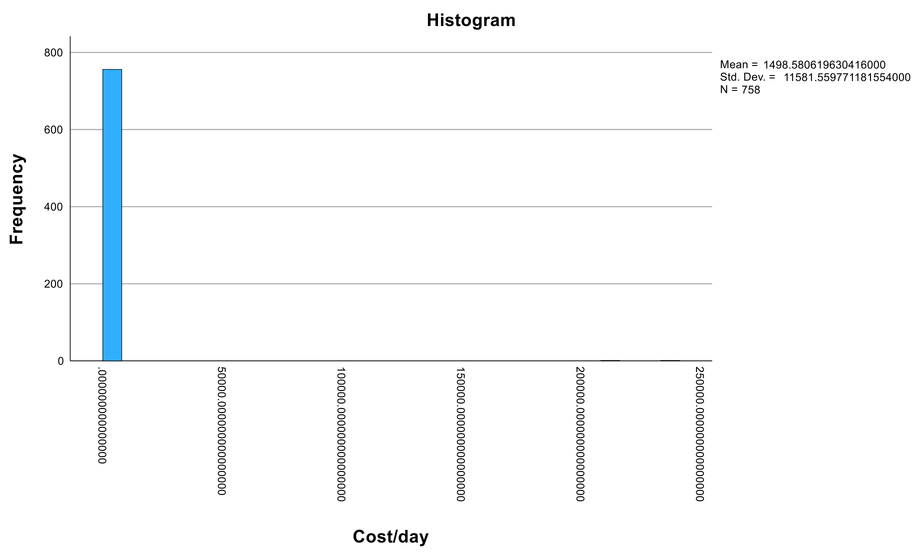


```
Cost/day Stem-and-Leaf Plot

 Frequency    Stem &  Leaf

     5.00        2 .  57&
     5.00        3 .  4&
     7.00        4 .  49&
    61.00        5 .  2333445556666777788899999999&
   127.00        6 .  0000111112222223333333333444445555555666666777777788999999999
   123.00        7 .  0000000111222222222333344444444455555555666666677778888889999
    92.00        8 .  00001111112222333344444455555666677788899999
   104.00        9 .  000112222223333334445555566666667777788888888999999
    76.00       10 .  00011111122223344444555666777788999
    53.00       11 .  0000011122233445666677789
    45.00       12 .  000122355566778899&
    19.00       13 .  00022468&
    12.00       14 .  4456&
     8.00       15 .  13&
    21.00 Extremes    (>=1597)

 Stem width:  100.0000
 Each leaf:        2 case(s)
```

& denotes fractional leaves.

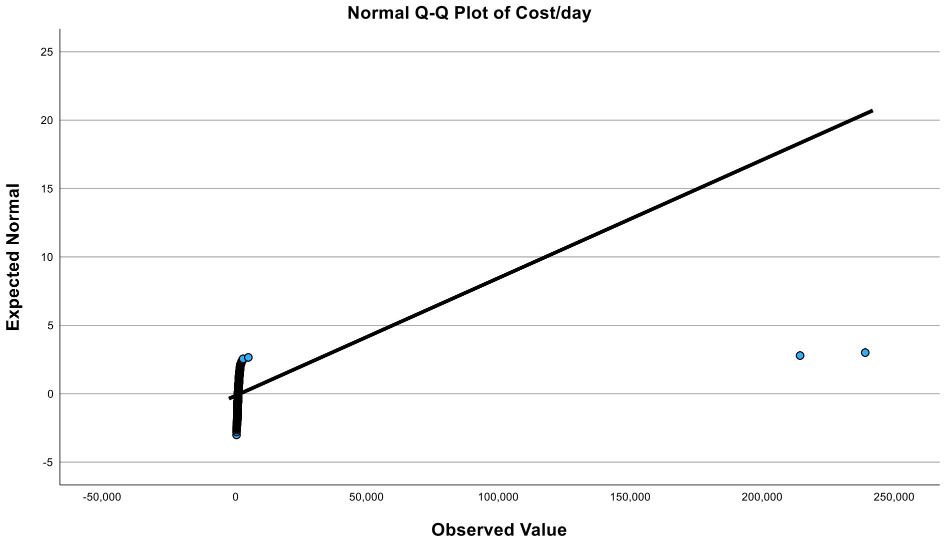

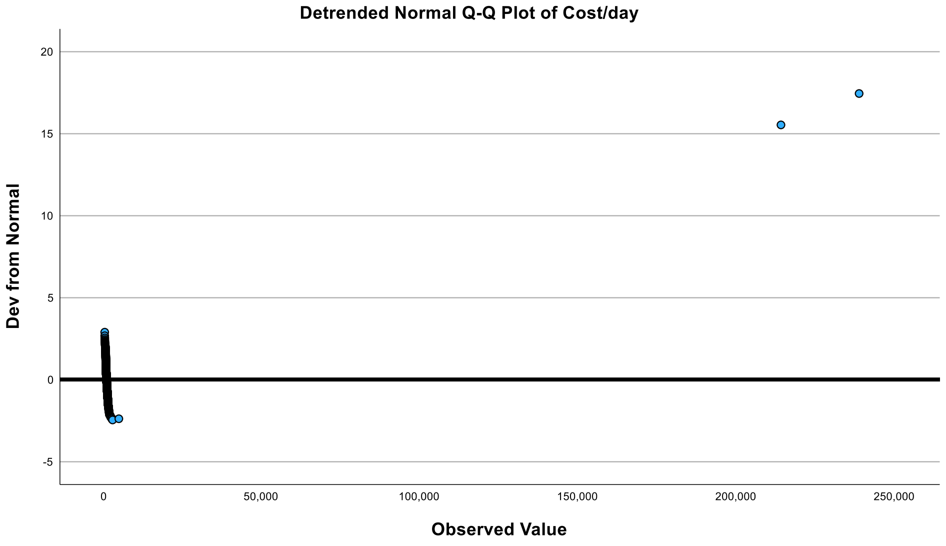

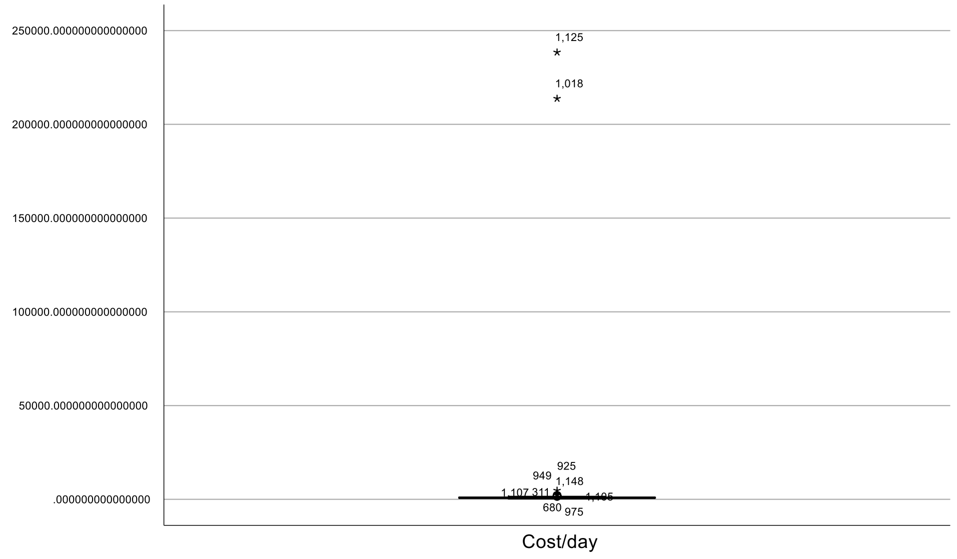

Explore SEVERITY: MODERATELY-SEVERE

Notes

Output Created		09-MAY-2023 11:02:55
Comments		
Input	Data	C:\Users\paho9\OneDrive\Documente\Doctorat - Stratificarea severității și predicția prognosticului în faza incipientă a Pancreatitei Acute\Registru Pancreatite Acute - BUC-API\Baza date nou\Articole\Articol cost\DB_corect_COST.sav
	Active Dataset	DataSet1

	Filter	RAC_Severity = 2 (FILTER)
	Weight	<none>
	Split File	<none>
	N of Rows in Working Data File	542
Missing Value Handling	Definition of Missing	User-defined missing values for dependent variables are treated as missing.
	Cases Used	Statistics are based on cases with no missing values for any dependent variable or factor used.
Syntax		EXAMINE VARIABLES=Costday /PLOT BOXPLOT STEMLEAF HISTOGRAM NPPLOT /COMPARE GROUPS /STATISTICS DESCRIPTIVES EXTREME /CINTERVAL 95 /MISSING LISTWISE /NOTOTAL.
Resources	Processor Time	00:00:00.44
	Elapsed Time	00:00:00.71

Case Processing Summary

	Cases					
	Valid		Missing		Total	
	N	Percent	N	Percent	N	Percent
Cost/day	542	100.0%	0	0.0%	542	100.0%

Descriptives

	Statistic	Std. Error

Cost/day	Mean			1625.109832172196500	592.705585305880800
	95% Confidence Interval for Mean		Lower Bound	460.823507237372950	
			Upper Bound	2789.396157107020400	
	5% Trimmed Mean			953.710138298019300	
	Median			923.758949771689500	
	Variance			190404551.682	
	Std. Deviation			9223.372036854777000	
	Minimum			328.333333333333300	
	Maximum			9223.372036854777000	
	Range			9223.372036854777000	
	Interquartile Range			448.842195512820600	
	Skewness			23.198	.105
	Kurtosis			539.399	.209

Extreme Values

			Case Number	Value
Cost/day	Highest	1	1049	9223.372036854777000
		2	1219	8662.000000000000000
		3	909	7655.230000000000000
		4	1247	6255.410000000000000
		5	1014	5974.465000000000000
	Lowest	1	476	328.333333333333300

	2	1473	355.864285714285760
	3	386	474.714285700000000
	4	646	498.666666666666700
	5	1430	499.000000000000000

Tests of Normality

	Kolmogorov-Smirnov[a]			Shapiro-Wilk		
	Statistic	df	Sig.	Statistic	df	Sig.
Cost/day	.464	542	<.001	.030	542	<.001

a. Lilliefors Significance Correction

Cost/day

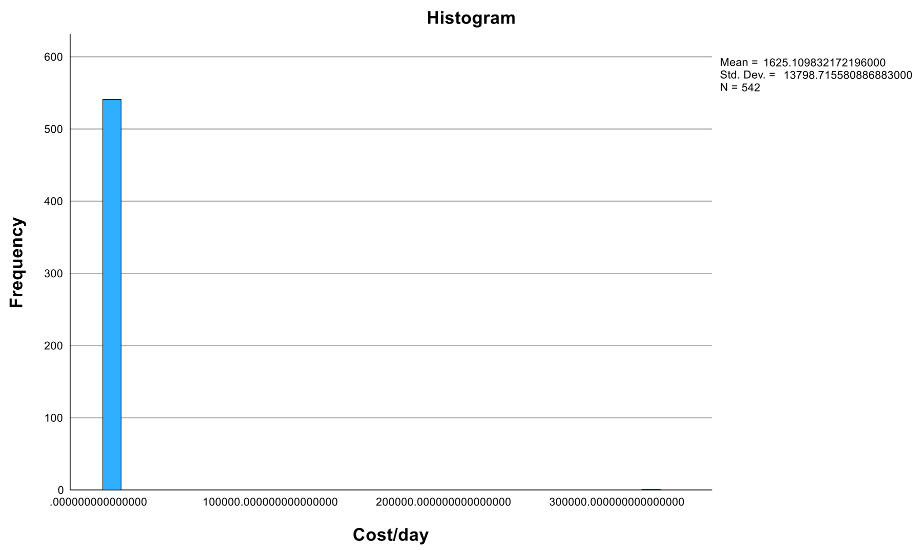

Cost/day Stem-and-Leaf Plot

```
Frequency    Stem &  Leaf

    2.00        3 .  25
    3.00        4 .  799
   23.00        5 .  02334455566667777788899
   89.00        6 .  00000011111112222222222333333334444444444444555555555555666666666777777778888888888999999999
   78.00        7 .  000000111111111111112223333333333344444444444555555555555666677777778888888888999
   67.00        8 .  0000111111222222333333333344444444455555556666666667777777778999999
   57.00        9 .  000000112223333333344444444445555555556666667777778889999999
   60.00       10 .  000000011111111222222222233334444455666667777788888889999999
   41.00       11 .  00012233333444455555555556666677778889999
   42.00       12 .  001111122233333344444446667777777888889999
   28.00       13 .  0011112222333344566777888899
   20.00       14 .  00001123334556678899
    7.00       15 .  0112578
    4.00       16 .  2567
    3.00       17 .  669
   18.00 Extremes    (>=1928)

Stem width:  100.0000
Each leaf:        1 case(s)
```

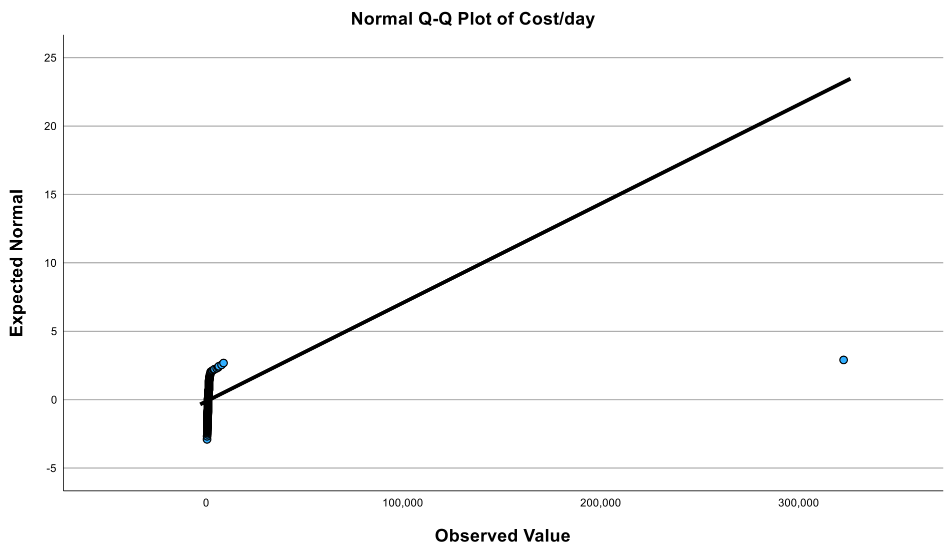

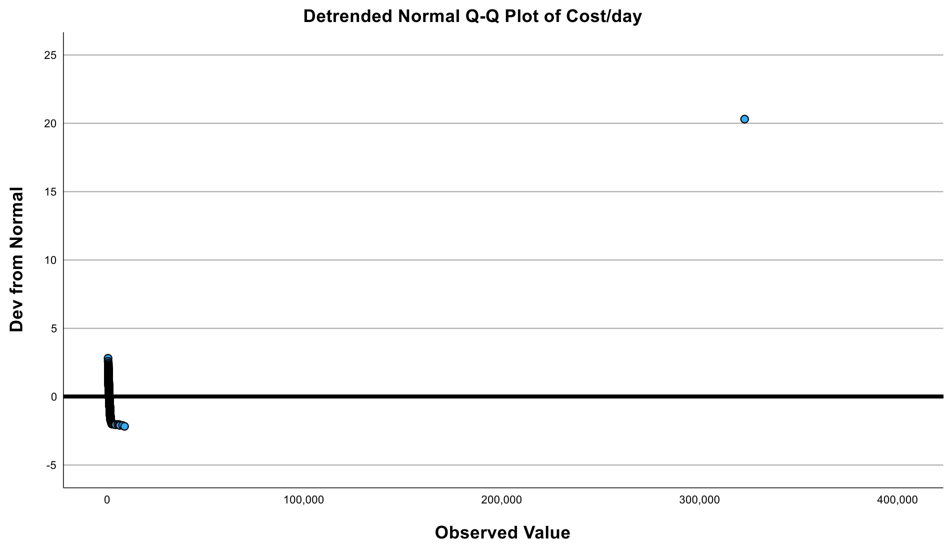

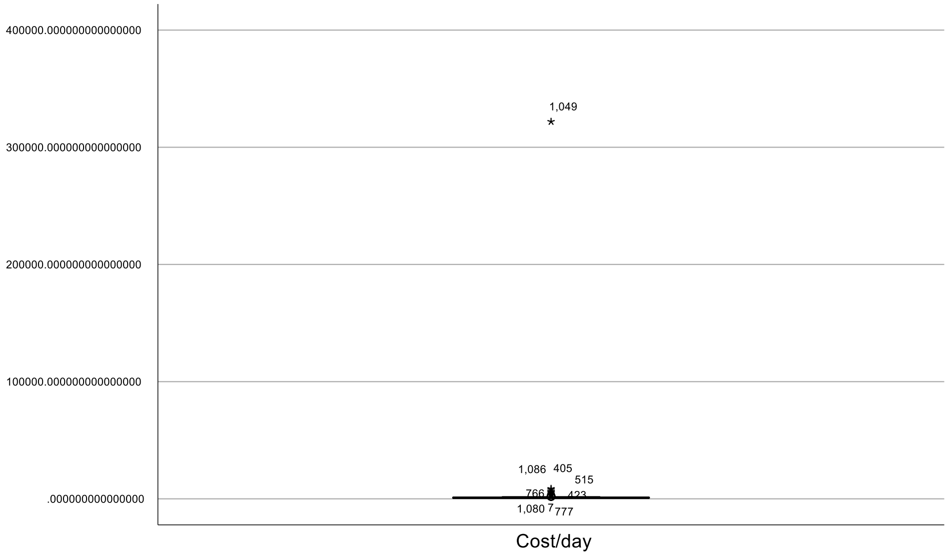

Explore SEVERITY: SEVERE

Notes

Output Created		09-MAY-2023 11:03:32
Comments		
Input	Data	C:\Users\paho9\OneDrive\Documente\Doctorat - Stratificarea severității și predicția prognosticului în faza incipientă a Pancreatitei Acute\Registru Pancreatite Acute - BUC-API\Baza date nou\Articole\Articol cost\DB_corect_COST.sav
	Active Dataset	DataSet1
	Filter	RAC_Severity = 3 (FILTER)
	Weight	<none>
	Split File	<none>
	N of Rows in Working Data File	173
Missing Value Handling	Definition of Missing	User-defined missing values for dependent variables are treated as missing.
	Cases Used	Statistics are based on cases with no missing values for any dependent variable or factor used.
Syntax		EXAMINE VARIABLES=Costday /PLOT BOXPLOT STEMLEAF HISTOGRAM NPPLOT /COMPARE GROUPS /STATISTICS DESCRIPTIVES EXTREME

		/CINTERVAL 95<br>/MISSING LISTWISE<br>/NOTOTAL.
Resources	Processor Time	00:00:00.31
	Elapsed Time	00:00:00.67

Case Processing Summary

	Cases					
	Valid		Missing		Total	
	N	Percent	N	Percent	N	Percent
Cost/day	173	100.0%	0	0.0%	173	100.0%

Descriptives

			Statistic	Std. Error
Cost/day	Mean		1463.9553363<br>27579300	76.570543600<br>961090
	95% Confidence Interval for Mean	Lower Bound	1312.8164027<br>21483400	
		Upper Bound	1615.0942699<br>33675600	
	5% Trimmed Mean		1336.6346935<br>34964500	
	Median		1143.7058823<br>52941200	
	Variance		1014307.329	
	Std. Deviation		1007.1282587<br>09374100	
	Minimum		325.40000000<br>0000000	
	Maximum		7595.1600000<br>00000000	
	Range		7269.7600000<br>00000000	
	Interquartile Range		703.47821428<br>5714300	
	Skewness		2.781	.185
	Kurtosis		10.669	.367

Extreme Values

			Case Number	Value
Cost/day	Highest	1	1274	7595.160000000000000
		2	702	6290.557500000000000
		3	678	4320.065000000000000
		4	737	4078.540000000000000
		5	928	4041.490000000000000
	Lowest	1	11	325.400000000000000
		2	526	509.115384615384640
		3	225	564.000000000000000
		4	1457	565.016000000000000
		5	138	581.000000000000000

Tests of Normality

	Kolmogorov-Smirnov[a]			Shapiro-Wilk		
	Statistic	df	Sig.	Statistic	df	Sig.
Cost/day	.226	173	<.001	.718	173	<.001

a. Lilliefors Significance Correction

Cost/day

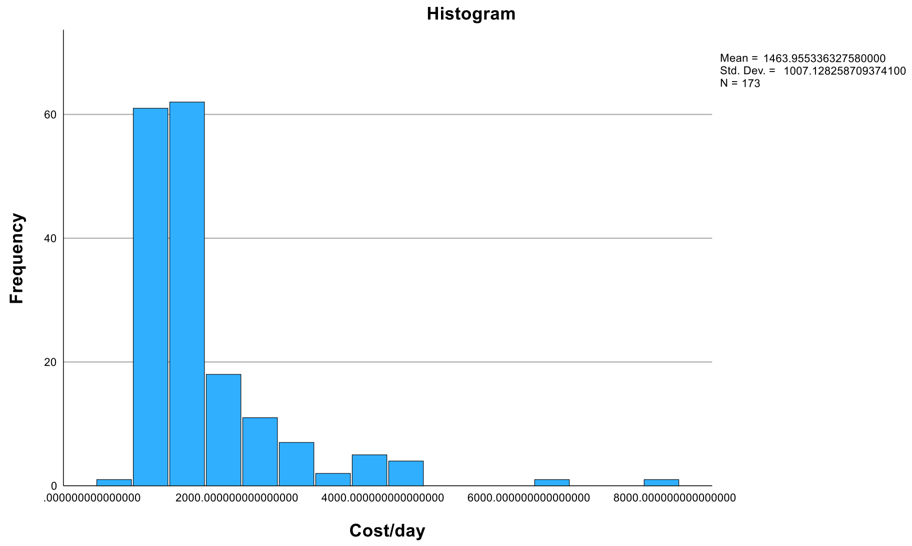


```
Cost/day Stem-and-Leaf Plot

 Frequency    Stem &  Leaf

     1.00        0 .  3
     5.00        0 .  55555
    23.00        0 .  66666666667777777777777
    33.00        0 .  888888888888888999999999999999999
    33.00        1 .  000000000000001111111111111111111
    26.00        1 .  22222222222233333333333333
    10.00        1 .  4445555555
     7.00        1 .  6666677
     4.00        1 .  8899
     3.00        2 .  011
     7.00        2 .  2222233
     3.00        2 .  455
     1.00        2 .  6
    17.00 Extremes    (>=2806)

 Stem width:  1000.000
 Each leaf:        1 case(s)
```

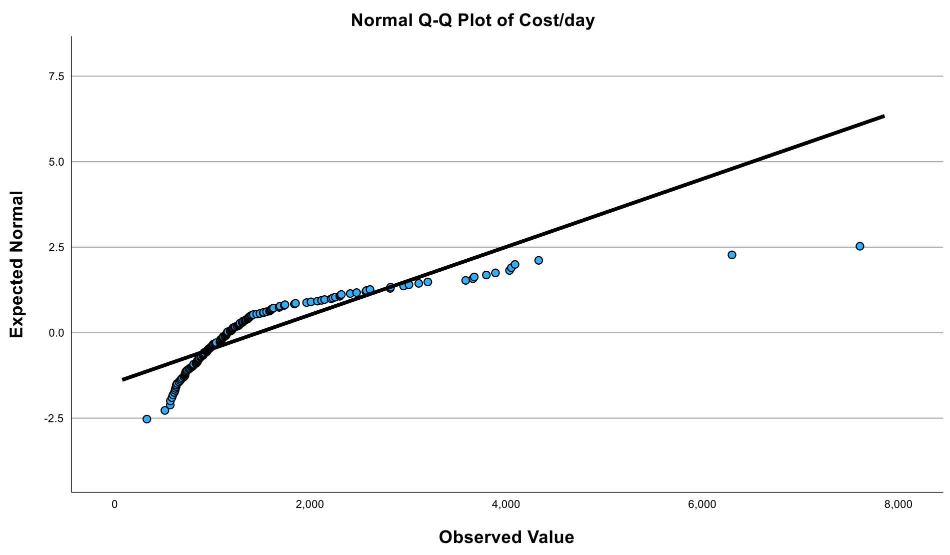
Normal Q-Q Plot of Cost/day
Expected Normal
Observed Value
7.5
5.0
2.5
0.0
-2.5
0
2,000
4,000
6,000
8,000

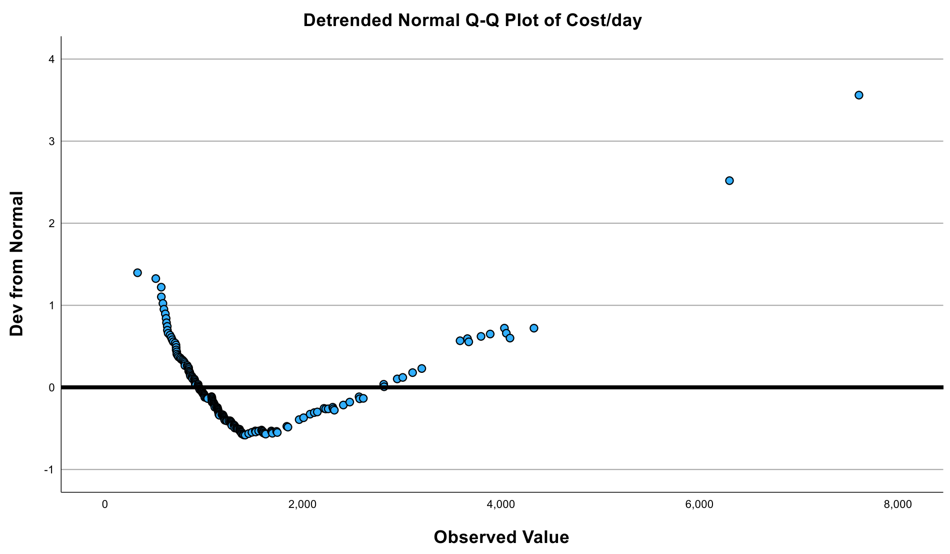
Detrended Normal Q-Q Plot of Cost/day
Dev from Normal
Observed Value
4
3
2
1
0
-1
0
2,000
4,000
6,000
8,000

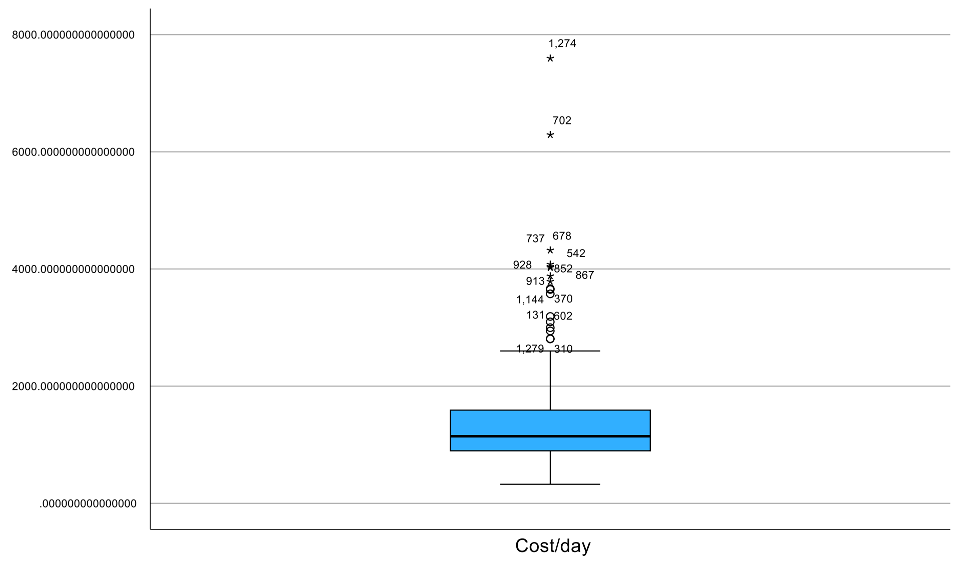

Nonparametric Tests

Notes

Output Created		09-MAY-2023 11:11:46
Comments		
Input	Data	C:\Users\paho9\OneDrive\Documente\Doctorat - Stratificarea severității și predicția prognosticului în faza incipientă a Pancreatitei Acute\Registru Pancreatite Acute - BUC-API\Baza date nou\Articole\Articol cost\DB_corect_COST.sav
	Active Dataset	DataSet1
	Filter	<none>

	Weight	<none>
	Split File	<none>
	N of Rows in Working Data File	1473
Syntax		NPTESTS /INDEPENDENT TEST (Costday) GROUP (RAC_Severity) KRUSKAL_WALLIS(COMPARE=PAIRWISE) /MISSING SCOPE=ANALYSIS USERMISSING=EXCLUDE /CRITERIA ALPHA=0.05 CILEVEL=95.
Resources	Processor Time	00:00:00.47
	Elapsed Time	00:00:00.74

Hypothesis Test Summary

	Null Hypothesis	Test	Sig.[a,b]
1	The distribution of Cost/day is the same across categories of RAC_Severity.	Independent-Samples Kruskal-Wallis Test	<.001

Hypothesis Test Summary

	Decision
1	Reject the null hypothesis.

a. The significance level is .050.

b. Asymptotic significance is displayed.

Independent-Samples Kruskal-Wallis Test

Cost/day across RAC_Severity

Independent-Samples Kruskal-Wallis Test Summary

Total N	1473
Test Statistic	93.679[a]
Degree Of Freedom	2
Asymptotic Sig.(2-sided test)	<.001

a. The test statistic is adjusted for ties.

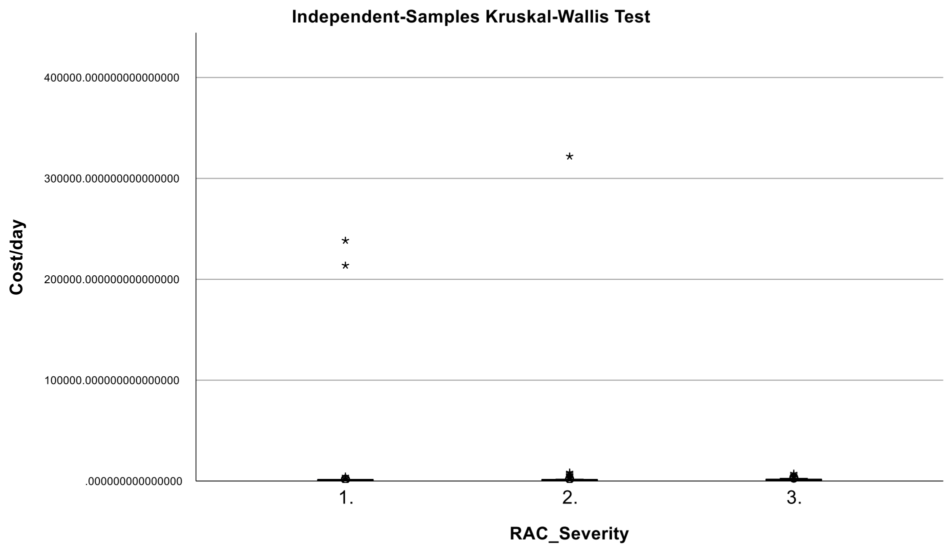

Pairwise Comparisons of RAC_Severity

Sample 1-Sample 2	Test Statistic	Std. Error	Std. Test Statistic	Sig.	Adj. Sig.[a]
1 (Mild) -2 (Moderate)	-96.696	23.927	-4.041	<.001	.000
1 (Mild) -3 (Severe)	-343.069	35.841	-9.572	<.001	.000
2 (Moderate) -3 (Severe)	-246.373	37.144	-6.633	<.001	.000

Each row tests the null hypothesis that the Sample 1 and Sample 2 distributions are the same.
Asymptotic significances (2-sided tests) are displayed. The significance level is .050.

a. Significance values have been adjusted by the Bonferroni correction for multiple tests.

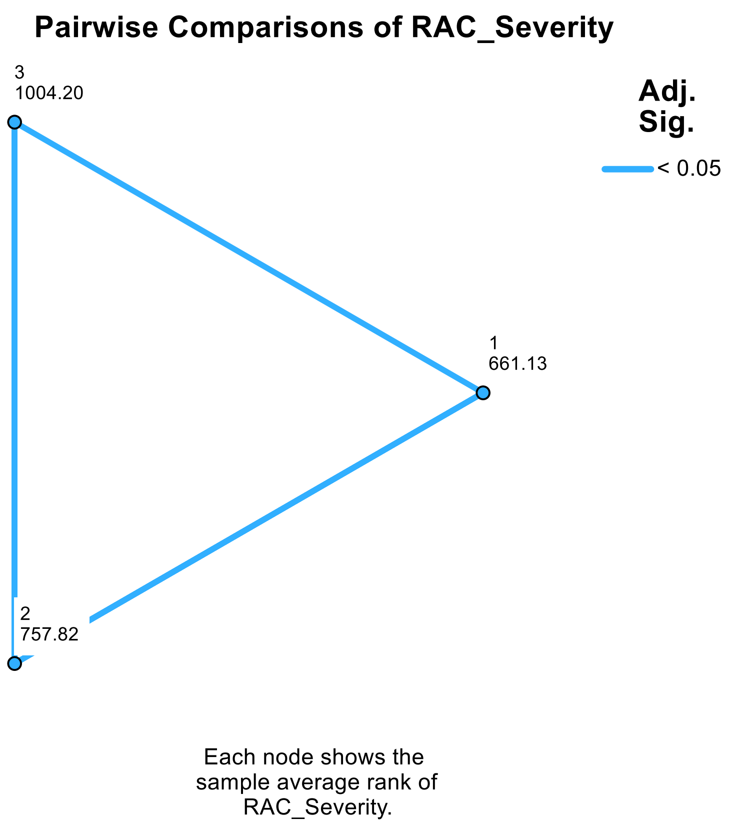
Pairwise Comparisons of RAC_Severity
3
1004.20
1
661.13
2
757.82
Adj. Sig.
< 0.05
Each node shows the sample average rank of RAC_Severity.

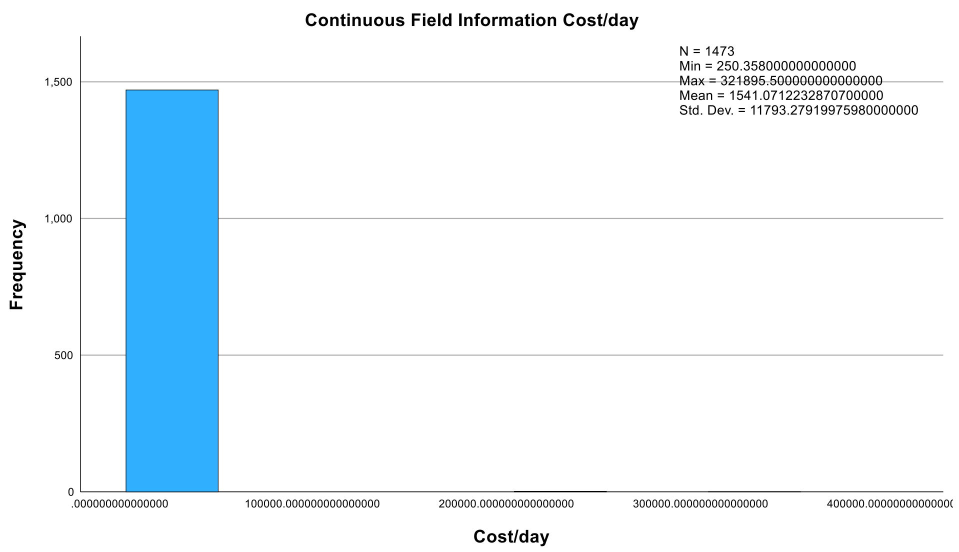

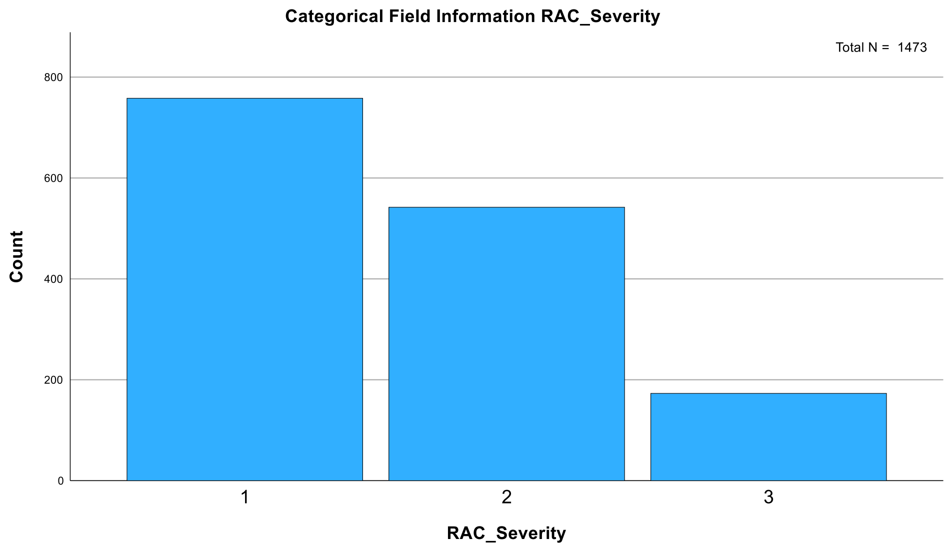

Explore HEALED/AMELIORATED

Notes

Output Created		09-MAY-2023 11:20:00
Comments		
Input	Data	C:\Users\paho9\OneDrive\Documente\Doctorat - Stratificarea severității și predicția prognosticului în faza incipientă a Pancreatitei Acute\Registru Pancreatite Acute - BUC-API\Baza date nou\Articole\Articol cost\DB_corect_COST.sav
	Active Dataset	DataSet1
	Filter	Outcome = 1 (FILTER)
	Weight	<none>
	Split File	<none>
	N of Rows in Working Data File	1234
Missing Value Handling	Definition of Missing	User-defined missing values for dependent variables are treated as missing.
	Cases Used	Statistics are based on cases with no missing values for any dependent variable or factor used.
Syntax		EXAMINE VARIABLES=Costday /PLOT BOXPLOT STEMLEAF HISTOGRAM NPPLOT /COMPARE GROUPS /STATISTICS DESCRIPTIVES EXTREME /CINTERVAL 95 /MISSING LISTWISE

		/NOTOTAL.
Resources	Processor Time	00:00:00.61
	Elapsed Time	00:00:00.85

Case Processing Summary

	Cases					
	Valid		Missing		Total	
	N	Percent	N	Percent	N	Percent
Cost/day	1234	100.0%	0	0.0%	1234	100.0%

Descriptives

			Statistic	Std. Error
Cost/day	Mean		1283.859071376312400	258.465595284443500
	95% Confidence Interval for Mean	Lower Bound	776.778049716829000	
		Upper Bound	1790.940093035795600	
	5% Trimmed Mean		904.050956342737800	
	Median		872.000000000000000	
	Variance		82436708.509	
	Std. Deviation		9079.466311906517000	
	Minimum		250.358000000000000	
	Maximum		9223.372036854777000	
	Range		9223.372036854777000	
	Interquartile Range		394.429895833333260	
	Skewness		24.886	.070
	Kurtosis		620.723	.139

Extreme Values

			Case Number	Value
Cost/day	Highest	1	1125	9223.372036854777000
		2	1018	9223.372036854777000
		3	611	2399.095238095238000
		4	918	2292.116000000000000
		5	1134	2217.525526315789600
	Lowest	1	492	250.358000000000000
		2	257	277.444444444444460
		3	11	325.400000000000000
		4	47	346.000000000000000
		5	1473	355.864285714285760

Tests of Normality

	Kolmogorov-Smirnov[a]			Shapiro-Wilk		
	Statistic	df	Sig.	Statistic	df	Sig.
Cost/day	.471	1234	<.001	.026	1234	<.001

a. Lilliefors Significance Correction

Cost/day

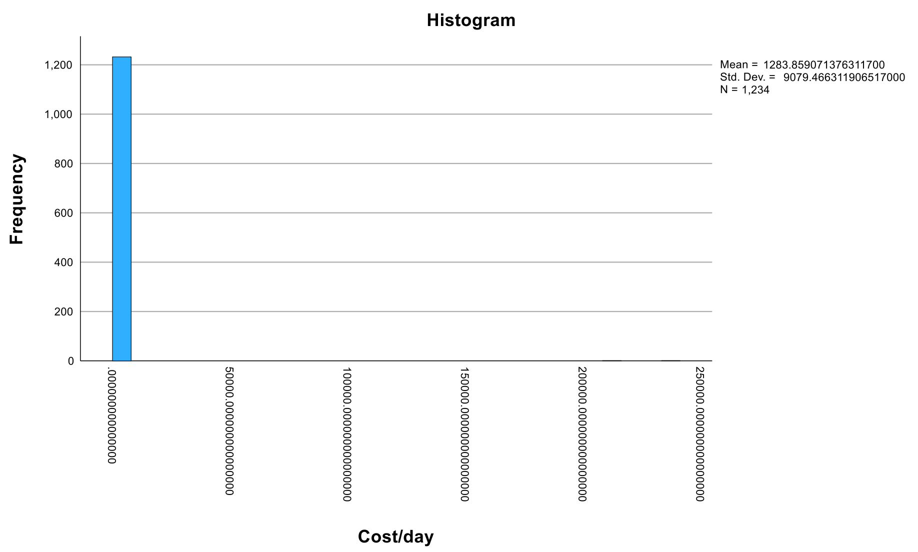

Cost/day Stem-and-Leaf Plot

```
 Frequency    Stem &  Leaf

     2.00        2 .  &
     2.00        3 .  &
     3.00        3 .  &
     3.00        4 .  4&
     6.00        4 .  99&
    21.00        5 .  0223333444&
    61.00        5 .  5555666666677777788888899999999
   102.00        6 .  000000111111112222222222222333333333333344444444444
   109.00        6 .  55555555555566666666666677777777777788888889999999999999
   105.00        7 .  0000000000111111111112222222222233333333344444444444444
    90.00        7 .  555555555555556666666666677777778888888888899999
    79.00        8 .  00000001111111222222223333333334444444444
    65.00        8 .  5555555566666667777778889999999
    68.00        9 .  000000111222222223333333334444444
    87.00        9 .  555555556666666667777777788888888899999999
    73.00       10 .  0000001111111111222222222233334444444
    62.00       10 .  55556666677777777888888899999999
    51.00       11 .  00000001111222233333334444
    45.00       11 .  5555566667777788889999
    43.00       12 .  00000111222233334444
```

```
   46.00       12 .  55556667777778888899999
   25.00       13 .  00001222334
   21.00       13 .  5566778889
   15.00       14 .  013344
   11.00       14 .  5678&
    9.00       15 .  113&
    6.00       15 .  79&
    4.00       16 .  2&
    6.00       16 .  77&
   14.00 Extremes    (>=1709)

Stem width:  100.0000
Each leaf:        2 case(s)


& denotes fractional leaves.
```

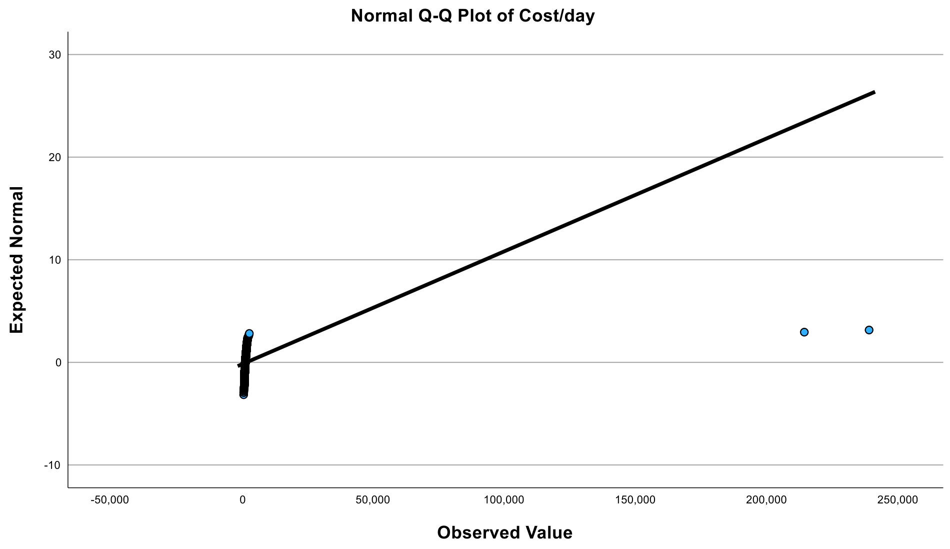

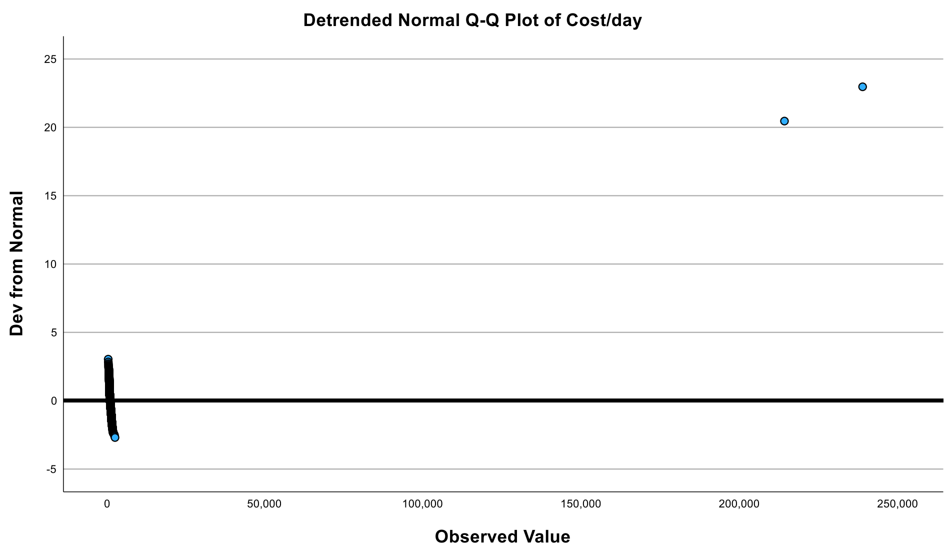

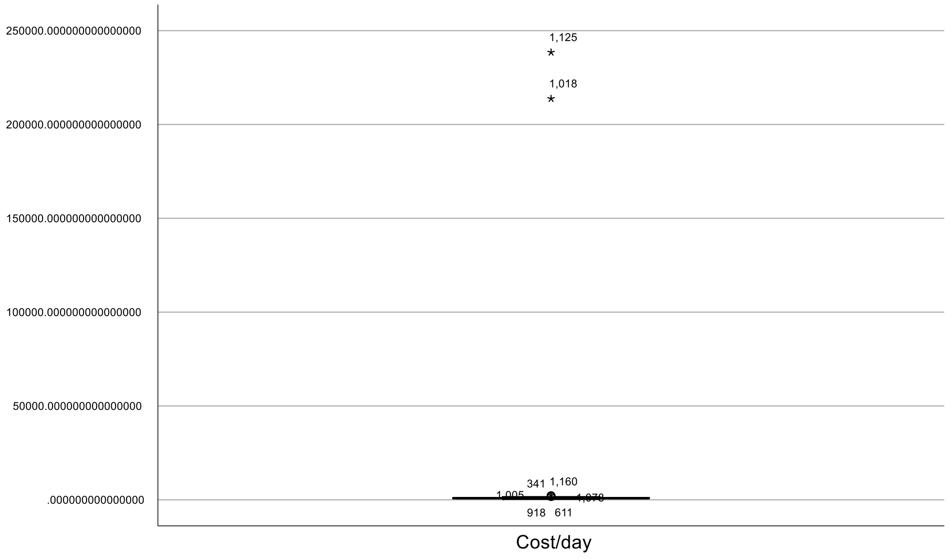

Explore TRANSFER

Notes

Output Created		09-MAY-2023 11:21:05
Comments		
Input	Data	C:\Users\paho9\OneDrive\Documente\Doctorat - Stratificarea severității și predicția prognosticului în faza incipientă a Pancreatitei Acute\Registru Pancreatite Acute - BUC-API\Baza date nou\Articole\Articol cost\DB_corect_COST.sav
	Active Dataset	DataSet1
	Filter	Outcome = 3 (FILTER)
	Weight	<none>
	Split File	<none>
	N of Rows in Working Data File	54
Missing Value Handling	Definition of Missing	User-defined missing values for dependent variables are treated as missing.
	Cases Used	Statistics are based on cases with no missing values for any dependent variable or factor used.
Syntax		EXAMINE VARIABLES=Costday /PLOT BOXPLOT STEMLEAF HISTOGRAM NPPLOT /COMPARE GROUPS /STATISTICS DESCRIPTIVES EXTREME /CINTERVAL 95 /MISSING LISTWISE

		/NOTOTAL.
Resources	Processor Time	00:00:00.42
	Elapsed Time	00:00:00.61

Case Processing Summary

	Cases					
	Valid		Missing		Total	
	N	Percent	N	Percent	N	Percent
Cost/day	54	100.0%	0	0.0%	54	100.0%

Descriptives

			Statistic	Std. Error
Cost/day	Mean		7091.248120278177000	5939.986606093107000
	95% Confidence Interval for Mean	Lower Bound	-4822.856227132785500	
		Upper Bound	9223.372036854777000	
	5% Trimmed Mean		1138.746690569716300	
	Median		1085.410000000000000	
	Variance		1905305807.551	
	Std. Deviation		9223.372036854777000	
	Minimum		306.452666666666700	
	Maximum		9223.372036854777000	
	Range		9223.372036854777000	
	Interquartile Range		445.425833333333230	
	Skewness		7.347	.325
	Kurtosis		53.989	.639

Extreme Values

			Case Number	Value
Cost/day	Highest	1	1049	9223.372036854777000
		2	602	2942.240000000000000
		3	1072	2067.000000000000000
		4	1208	1998.500000000000000
		5	1092	1842.840000000000000
	Lowest	1	916	306.452666666666700
		2	752	533.016666666666700
		3	368	657.428571428571400
		4	1332	677.000000000000000
		5	69	765.043478260869600

Tests of Normality

	Kolmogorov-Smirnov[a]			Shapiro-Wilk		
	Statistic	df	Sig.	Statistic	df	Sig.
Cost/day	.519	54	<.001	.125	54	<.001

a. Lilliefors Significance Correction

Cost/day

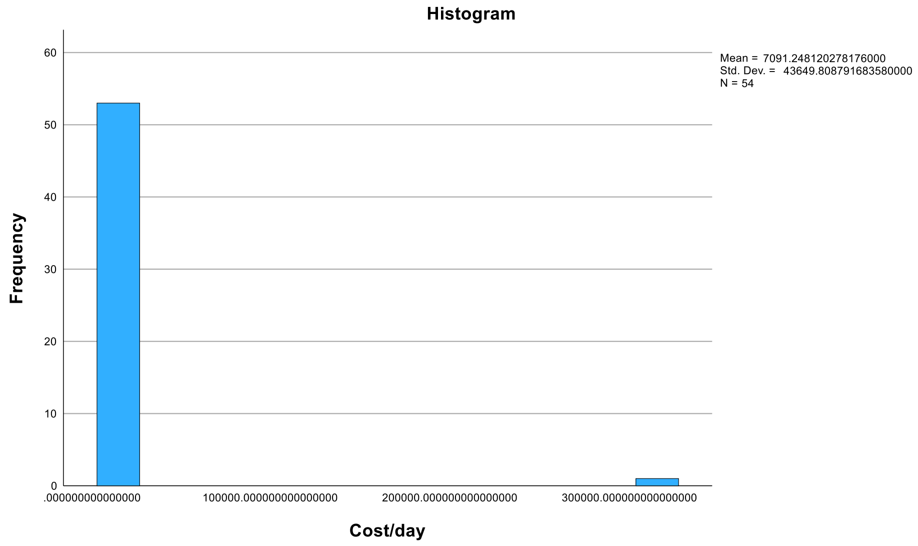


```
Cost/day Stem-and-Leaf Plot

 Frequency    Stem &  Leaf

     1.00        0 .  3
     1.00        0 .  5
     6.00        0 .  667777
    14.00        0 .  88888888899999
    12.00        1 .  000000111111
     9.00        1 .  222223333
     4.00        1 .  4445
     2.00        1 .  77
     1.00        1 .  8
     4.00 Extremes    (>=1999)

 Stem width:  1000.000
 Each leaf:        1 case(s)
```

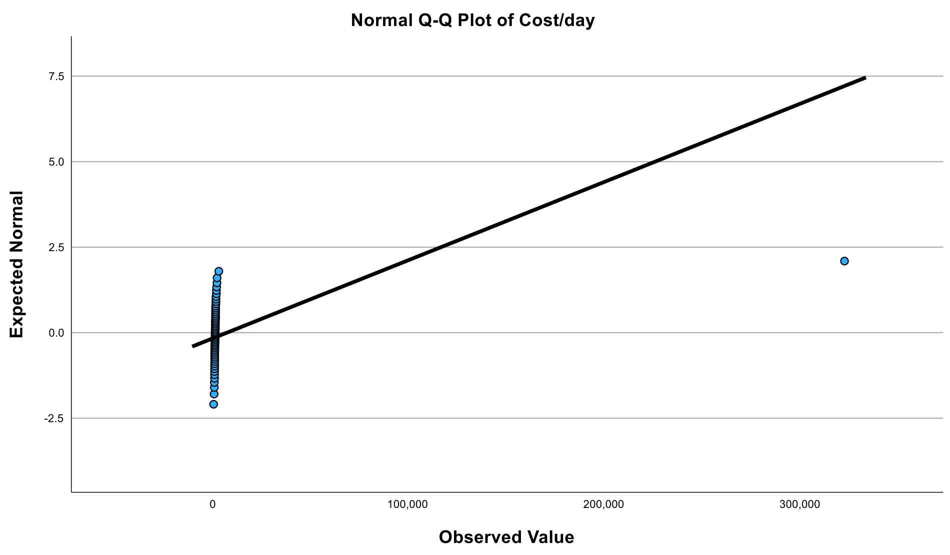
Normal Q-Q Plot of Cost/day
Expected Normal
7.5
5.0
2.5
0.0
-2.5
0
100,000
200,000
300,000
Observed Value

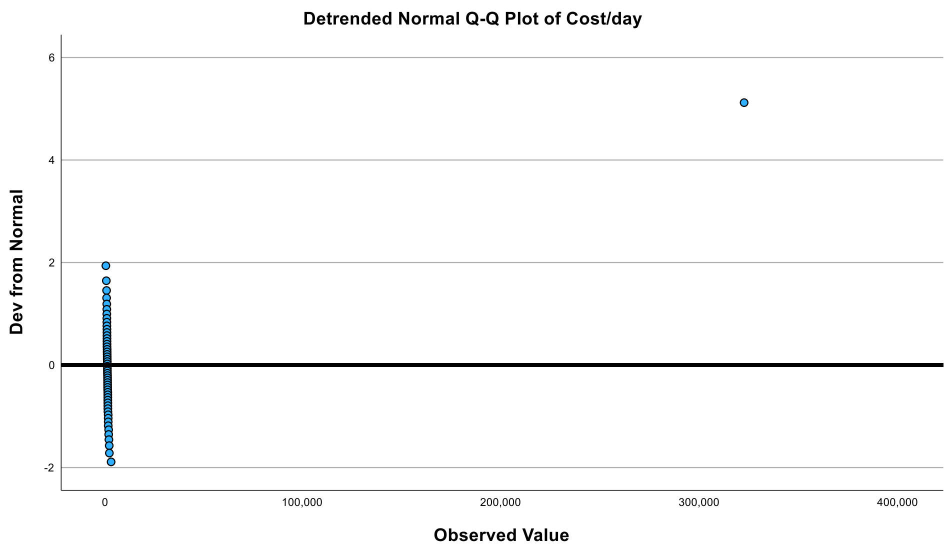
Detrended Normal Q-Q Plot of Cost/day
Dev from Normal
6
4
2
0
-2
0
100,000
200,000
300,000
400,000
Observed Value

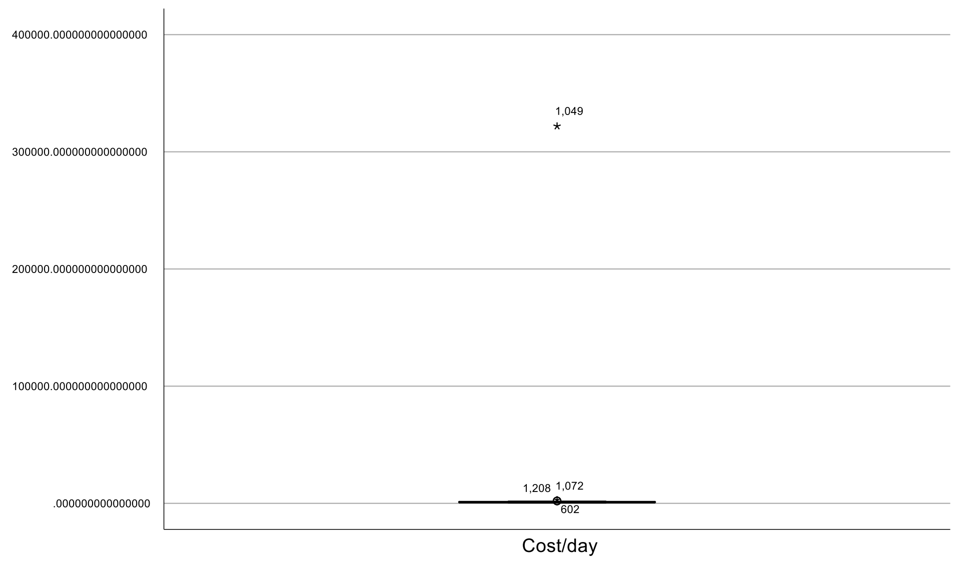

Explore DISCHARGE-AT-WILL

Notes

Output Created		09-MAY-2023 11:21:34
Comments		
Input	Data	C:\Users\paho9\OneDrive\Documente\Doctorat - Stratificarea severității și predicția prognosticului în faza incipientă a Pancreatitei Acute\Registru Pancreatite Acute - BUC-API\Baza date nou\Articole\Articol cost\DB_corect_COST.sav
	Active Dataset	DataSet1
	Filter	Outcome = 4 (FILTER)
	Weight	<none>

	Split File	<none>
	N of Rows in Working Data File	101
Missing Value Handling	Definition of Missing	User-defined missing values for dependent variables are treated as missing.
	Cases Used	Statistics are based on cases with no missing values for any dependent variable or factor used.
Syntax		EXAMINE VARIABLES=Costday /PLOT BOXPLOT STEMLEAF HISTOGRAM NPPLOT /COMPARE GROUPS /STATISTICS DESCRIPTIVES EXTREME /CINTERVAL 95 /MISSING LISTWISE /NOTOTAL.
Resources	Processor Time	00:00:00.45
	Elapsed Time	00:00:00.66

Case Processing Summary

	Cases					
	Valid		Missing		Total	
	N	Percent	N	Percent	N	Percent
Cost/day	101	100.0%	0	0.0%	101	100.0%

Descriptives

			Statistic	Std. Error
Cost/day	Mean		1013.509126163585800	39.372298089547420
	95% Confidence Interval for Mean	Lower Bound	935.395608135120900	

		Upper Bound	1091.622644192050800	
	5% Trimmed Mean		997.615670904248300	
	Median		940.380000000000000	
	Variance		156567.964	
	Std. Deviation		395.686698717647230	
	Minimum		252.000000000000000	
	Maximum		2459.000000000000000	
	Range		2207.000000000000000	
	Interquartile Range		529.082083333333200	
	Skewness		.871	.240
	Kurtosis		1.435	.476

Extreme Values

			Case Number	Value
Cost/day	Highest	1	311	2459.000000000000000
		2	1319	2137.000000000000000
		3	92	2070.000000000000000
		4	841	1726.865714285714100
		5	1174	1684.180000000000000
	Lowest	1	206	252.000000000000000
		2	464	278.723333333333300
		3	232	291.500000000000000

	4	476	328.33333333 3333300
	5	88	344.14285714 2857170

Tests of Normality

	Kolmogorov-Smirnov[a]			Shapiro-Wilk		
	Statistic	df	Sig.	Statistic	df	Sig.
Cost/day	.138	101	<.001	.952	101	.001

a. Lilliefors Significance Correction

Cost/day

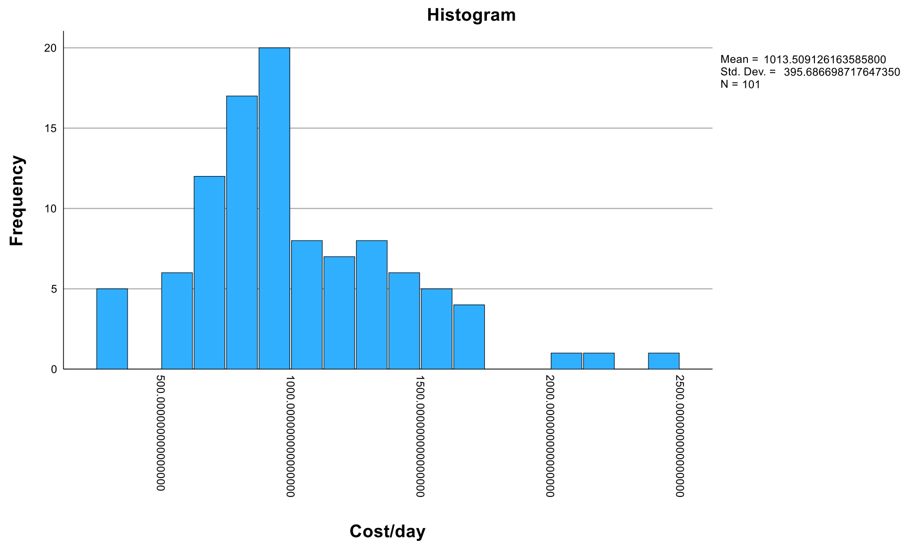


```
Cost/day Stem-and-Leaf Plot

 Frequency     Stem &  Leaf

     3.00        2 .  579
```

```
     2.00        3 .  24
      .00        4 .
     4.00        5 .  0569
    10.00        6 .  0133455679
    12.00        7 .  012355677889
    14.00        8 .  02444555678899
    15.00        9 .  000134456678899
     6.00       10 .  000049
     7.00       11 .  0223456
     3.00       12 .  046
     9.00       13 .  022334678
     4.00       14 .  0245
     5.00       15 .  11289
     3.00       16 .  578
     1.00       17 .  2
     3.00 Extremes    (>=2070)

 Stem width:  100.0000
 Each leaf:        1 case(s)
```

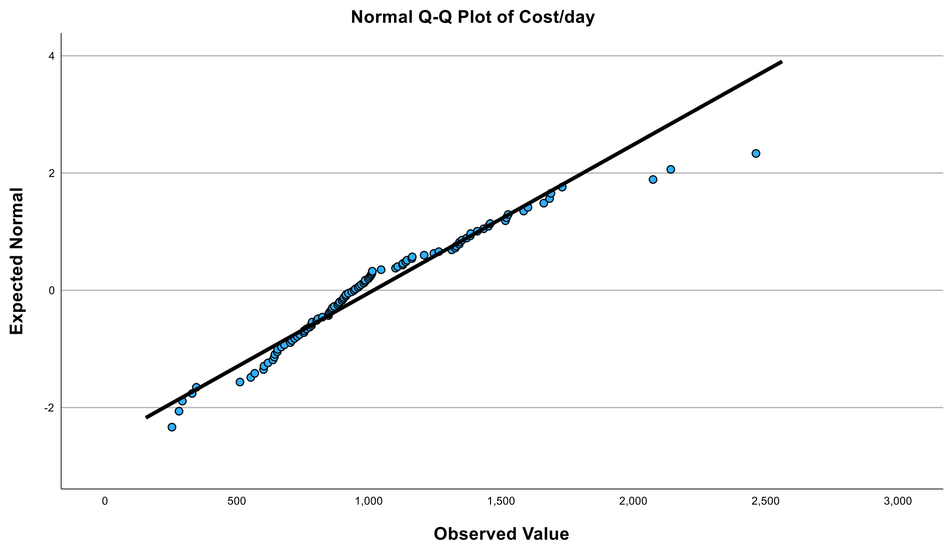

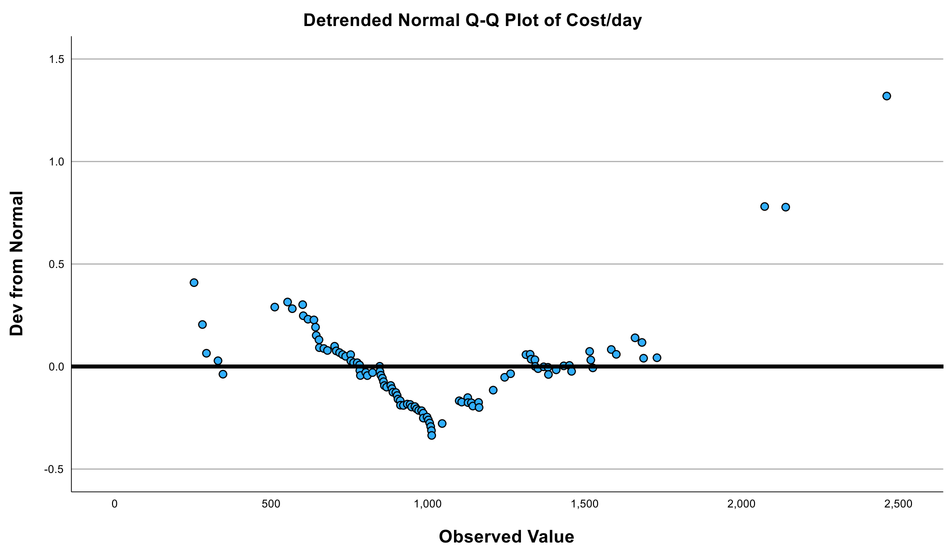

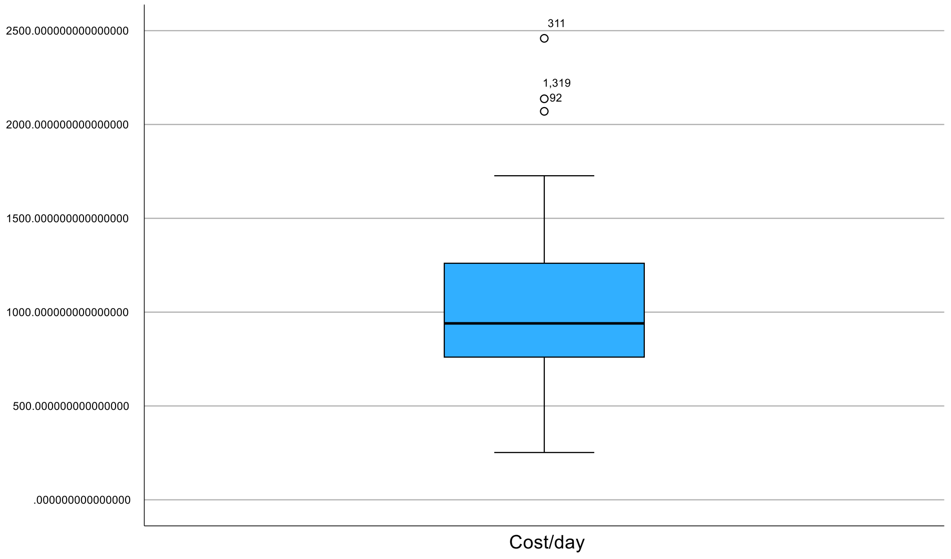

Explore DECEASED

Notes

Output Created		09-MAY-2023 11:22:31
Comments		
Input	Data	C:\Users\paho9\OneDrive\Documente\Doctorat - Stratificarea severității și predicția prognosticului în faza incipientă a Pancreatitei Acute\Registru Pancreatite Acute - BUC-API\Baza date nou\Articole\Articol cost\DB_corect_COST.sav
	Active Dataset	DataSet1
	Filter	Outcome = 6 (FILTER)
	Weight	<none>
	Split File	<none>
	N of Rows in Working Data File	77
Missing Value Handling	Definition of Missing	User-defined missing values for dependent variables are treated as missing.
	Cases Used	Statistics are based on cases with no missing values for any dependent variable or factor used.
Syntax		EXAMINE VARIABLES=Costday /PLOT BOXPLOT STEMLEAF HISTOGRAM NPPLOT /COMPARE GROUPS /STATISTICS DESCRIPTIVES EXTREME /CINTERVAL 95 /MISSING LISTWISE

		/NOTOTAL.
Resources	Processor Time	00:00:00.41
	Elapsed Time	00:00:00.67

Case Processing Summary

	Cases					
	Valid		Missing		Total	
	N	Percent	N	Percent	N	Percent
Cost/day	77	100.0%	0	0.0%	77	100.0%

Descriptives

			Statistic	Std. Error
Cost/day	Mean		2498.8134265 92197000	201.73635736 4237250
	95% Confidence Interval for Mean	Lower Bound	2097.0206492 60437500	
		Upper Bound	2900.6062039 23957000	
	5% Trimmed Mean		2321.1436063 06546600	
	Median		2091.2000000 00000000	
	Variance		3133711.957	
	Std. Deviation		1770.2293515 13392400	
	Minimum		499.00000000 0000000	
	Maximum		8662.0000000 00000000	
	Range		8163.0000000 00000000	
	Interquartile Range		2259.2800718 39080400	
	Skewness		1.479	.274
	Kurtosis		2.213	.541

Extreme Values

			Case Number	Value
Cost/day	Highest	1	1219	8662.000000000000000
		2	909	7655.230000000000000
		3	1274	7595.160000000000000
		4	702	6290.557500000000000
		5	1247	6255.410000000000000
	Lowest	1	1430	499.000000000000000
		2	225	564.000000000000000
		3	1314	648.555555555555500
		4	316	685.250000000000000
		5	285	721.210526315789500

Tests of Normality

	Kolmogorov-Smirnov[a]			Shapiro-Wilk		
	Statistic	df	Sig.	Statistic	df	Sig.
Cost/day	.140	77	<.001	.859	77	<.001

a. Lilliefors Significance Correction

Cost/day

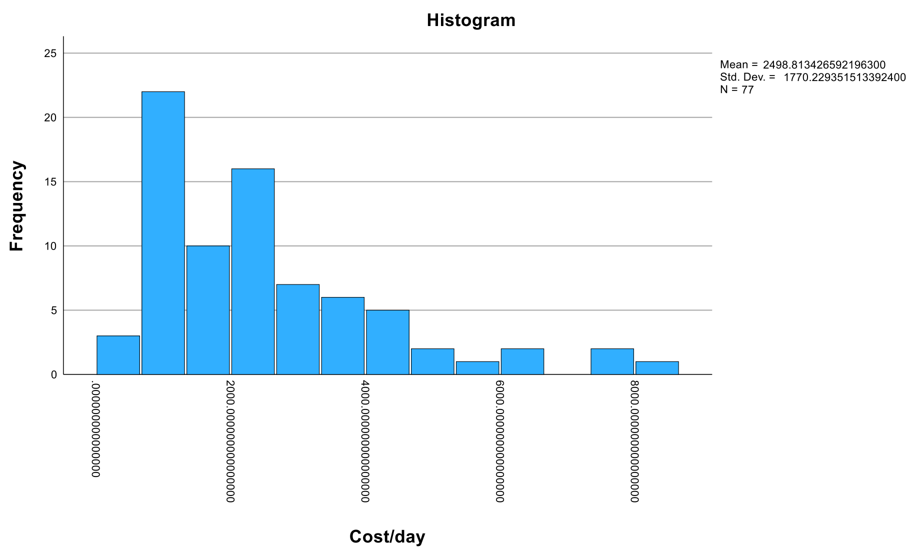


```
Cost/day Stem-and-Leaf Plot

 Frequency    Stem &  Leaf

    15.00        0 .  456677788888999
    20.00        1 .  00011112233455668999
    20.00        2 .  00001122223455566889
     9.00        3 .  001566678
     6.00        4 .  000037
     2.00        5 .  29
     1.00        6 .  2
     4.00 Extremes    (>=6291)

 Stem width:  1000.000
 Each leaf:        1 case(s)
```

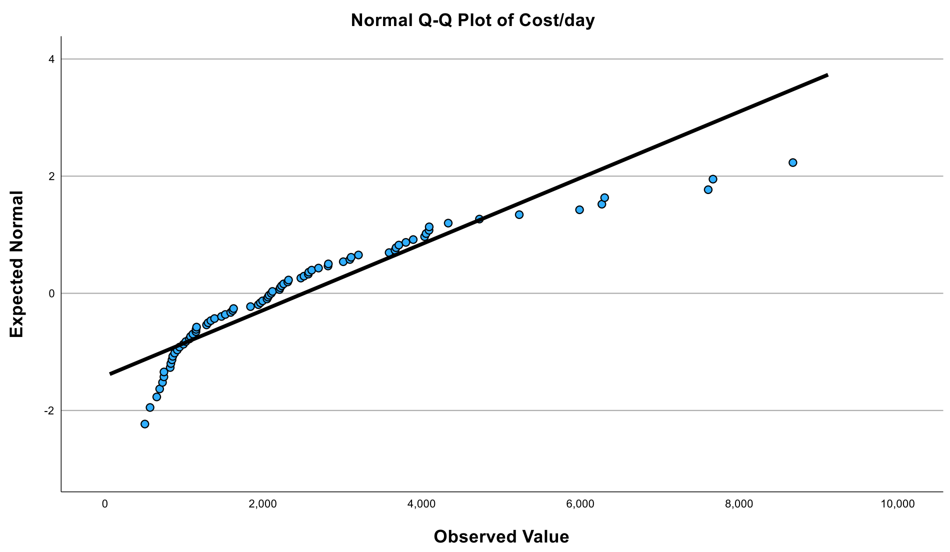
Normal Q-Q Plot of Cost/day
Expected Normal
Observed Value
4
2
0
-2
0
2,000
4,000
6,000
8,000
10,000

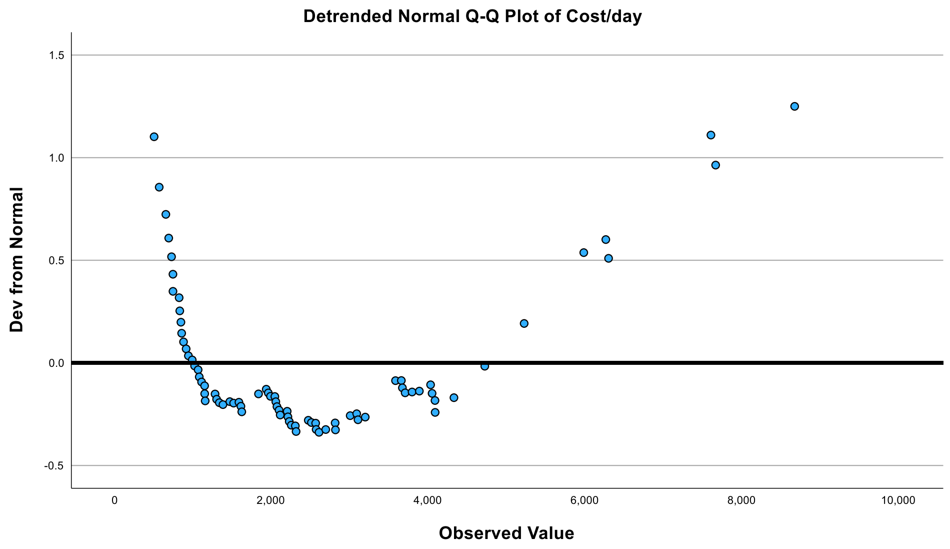
Detrended Normal Q-Q Plot of Cost/day
Dev from Normal
Observed Value
1.5
1.0
0.5
0.0
-0.5
0
2,000
4,000
6,000
8,000
10,000

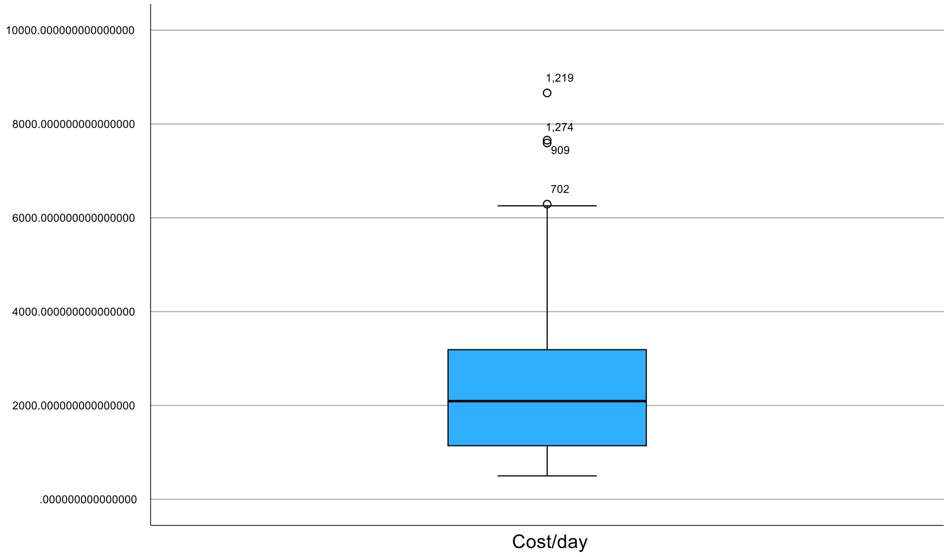

Nonparametric Tests

Notes

Output Created		09-MAY-2023 11:28:39
Comments		
Input	Data	C:\Users\paho9\OneDrive\Documente\Doctorat - Stratificarea severității și predicția prognosticului în faza incipientă a Pancreatitei Acute\Registru Pancreatite Acute - BUC-API\Baza date nou\Articole\Articol cost\DB_corect_COST.sav
	Active Dataset	DataSet1

	Filter	Outcome = 1 OR Outcome = 3 OR Outcome = 4 OR Outcome = 6 (FILTER)
	Weight	<none>
	Split File	<none>
	N of Rows in Working Data File	1466
Syntax		NPTESTS /INDEPENDENT TEST (Costday) GROUP (Outcome) KRUSKAL_WALLIS(COMPARE=PAIRWISE) /MISSING SCOPE=ANALYSIS USERMISSING=EXCLUDE /CRITERIA ALPHA=0.05 CILEVEL=95.
Resources	Processor Time	00:00:00.45
	Elapsed Time	00:00:00.78

Hypothesis Test Summary

	Null Hypothesis	Test	Sig.[a,b]
1	The distribution of Cost/day is the same across categories of Outcome.	Independent-Samples Kruskal-Wallis Test	<.001

Hypothesis Test Summary

	Decision
1	Reject the null hypothesis.

a. The significance level is .050.

b. Asymptotic significance is displayed.

Independent-Samples Kruskal-Wallis Test

Cost/day across Outcome

Independent-Samples Kruskal-Wallis Test Summary

Total N	1466
Test Statistic	126.047[a]
Degree Of Freedom	3
Asymptotic Sig.(2-sided test)	<.001

a. The test statistic is adjusted for ties.

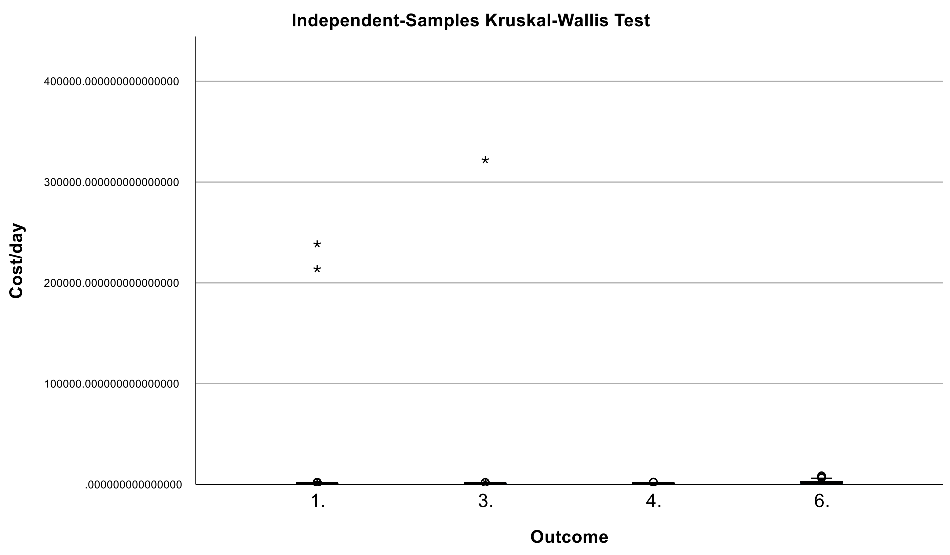

Pairwise Comparisons of Outcome

Sample 1-Sample 2	Test Statistic	Std. Error	Std. Test Statistic	Sig.	Adj. Sig.[a]
1(Healed) – 4 (At-will)	-100.883	43.814	-2.303	.021	.128
1 (healed) -3 (transfer)	-273.217	58.857	-4.642	<.001	.000
1 (healed) -6 (deceased)	-514.382	49.727	-10.344	<.001	.000
4 (at-will) -3 (transfer)	172.334	71.367	2.415	.016	.094
4 (at-will) -6 (deceased)	-413.499	64.046	-6.456	<.001	.000
3 (transfer) -6 (deceased)	-241.165	75.142	-3.209	.001	.008

Each row tests the null hypothesis that the Sample 1 and Sample 2 distributions are the same.
Asymptotic significances (2-sided tests) are displayed. The significance level is .050.

a. Significance values have been adjusted by the Bonferroni correction for multiple tests.

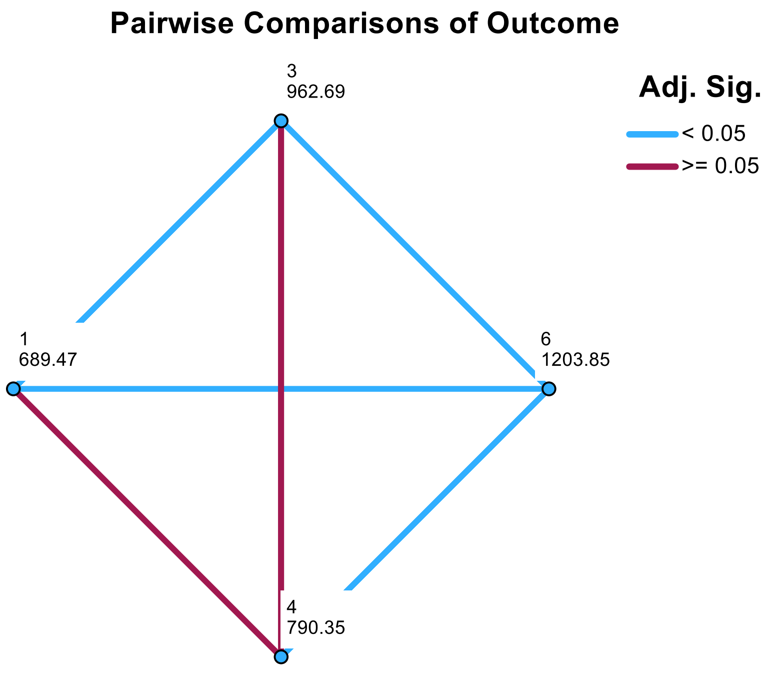

Each node shows the
sample average rank of
Outcome.

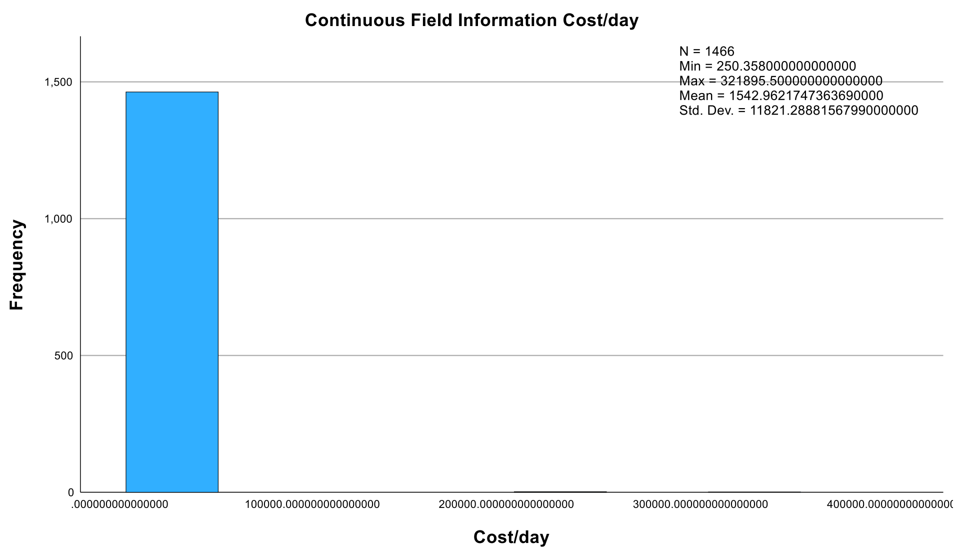

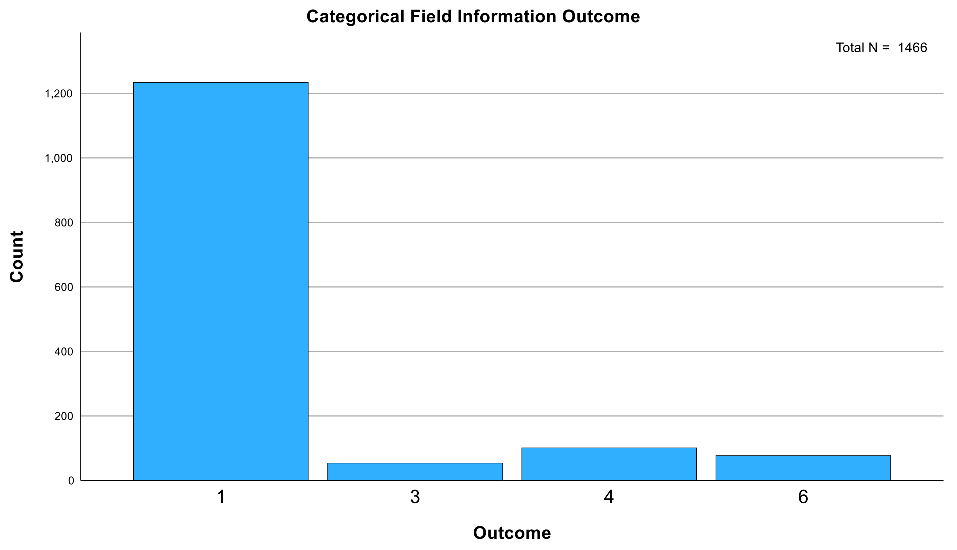

Explore ICU N

Notes

Output Created		09-MAY-2023 11:38:12
Comments		
Input	Data	C:\Users\paho9\OneDrive\Documente\Doctorat - Stratificarea severității și predicția prognosticului în faza incipientă a Pancreatitei Acute\Registru Pancreatite Acute - BUC-API\Baza date nou\Articole\Articol cost\DB_corect_COST.sav
	Active Dataset	DataSet1
	Filter	ICU_YN = 0 (FILTER)
	Weight	<none>
	Split File	<none>
	N of Rows in Working Data File	1348
Missing Value Handling	Definition of Missing	User-defined missing values for dependent variables are treated as missing.
	Cases Used	Statistics are based on cases with no missing values for any dependent variable or factor used.
Syntax		EXAMINE VARIABLES=Costday /PLOT BOXPLOT STEMLEAF HISTOGRAM NPPLOT /COMPARE GROUPS /STATISTICS DESCRIPTIVES EXTREME /CINTERVAL 95 /MISSING LISTWISE

		/NOTOTAL.
Resources	Processor Time	00:00:00.88
	Elapsed Time	00:00:01.27

Case Processing Summary

	Cases					
	Valid		Missing		Total	
	N	Percent	N	Percent	N	Percent
Cost/day	1348	100.0%	0	0.0%	1348	100.0%

Descriptives

			Statistic	Std. Error
Cost/day	Mean		1498.430289117780000	335.534106737521200
	95% Confidence Interval for Mean	Lower Bound	840.204075583752200	
		Upper Bound	2156.656502651807400	
	5% Trimmed Mean		903.462116017489400	
	Median		865.550000000000000	
	Variance		151762068.385	
	Std. Deviation		9223.372036854777000	
	Minimum		250.358000000000000	
	Maximum		9223.372036854777000	
	Range		9223.372036854777000	
	Interquartile Range		387.727499999999960	
	Skewness		22.172	.067
	Kurtosis		505.940	.133

Extreme Values

			Case Number	Value
Cost/day	Highest	1	1049	9223.372036854777000
		2	1125	9223.372036854777000
		3	1018	9223.372036854777000
		4	1144	3649.810000000000000
		5	131	2998.000000000000000
	Lowest	1	492	250.358000000000000
		2	206	252.000000000000000
		3	257	277.444444444444460
		4	464	278.723333333333300
		5	232	291.500000000000000

Tests of Normality

	Kolmogorov-Smirnov[a]			Shapiro-Wilk		
	Statistic	df	Sig.	Statistic	df	Sig.
Cost/day	.474	1348	<.001	.030	1348	<.001

a. Lilliefors Significance Correction

Cost/day

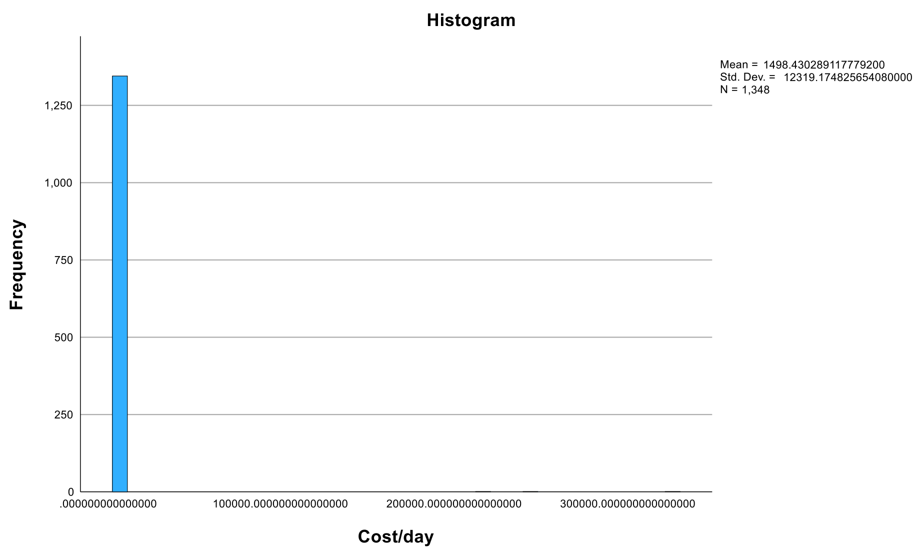

Cost/day Stem-and-Leaf Plot

```
 Frequency    Stem &  Leaf

     5.00        2 .  57&
     5.00        3 .  24&
     3.00        3 .  &
     3.00        4 .  4&
     7.00        4 .  99&
    22.00        5 .  022333344&
    66.00        5 .  5555566666667777778888899999999
   109.00        6 .  000000111111112222222222222333333333333333444444444444
   117.00        6 .  5555555555555566666666666677777777777778888888899999999999999
   112.00        7 .  00000000001111111111112222222222233333333333444444444444
   102.00        7 .  5555555555555556666666666677777777888888888888999999
    90.00        8 .  0000000111111112222222223333333334444444444444
    78.00        8 .  555555555566666666677777777888899999999
    76.00        9 .  0000000111122222222333333333334444444444
    98.00        9 .  5555555556666666666677777777888888888888999999999999
    73.00       10 .  000000011111111222222222333344444444
    64.00       10 .  5555666667777777777888889999999
    56.00       11 .  00000000111122222333333334444
    43.00       11 .  5555555666666777788999
    39.00       12 .  0000111222333444
```

```
   44.00       12 .  5556666777777888899999
   31.00       13 .  000011222223344
   19.00       13 .  556677889
   14.00       14 .  01344&
   15.00       14 .  566789
   12.00       15 .  01113&
    6.00       15 .  78&
    5.00       16 .  2&
    1.00       16 .  &
   33.00 Extremes    (>=1676)

Stem width:  100.0000
Each leaf:        2 case(s)


& denotes fractional leaves.
```

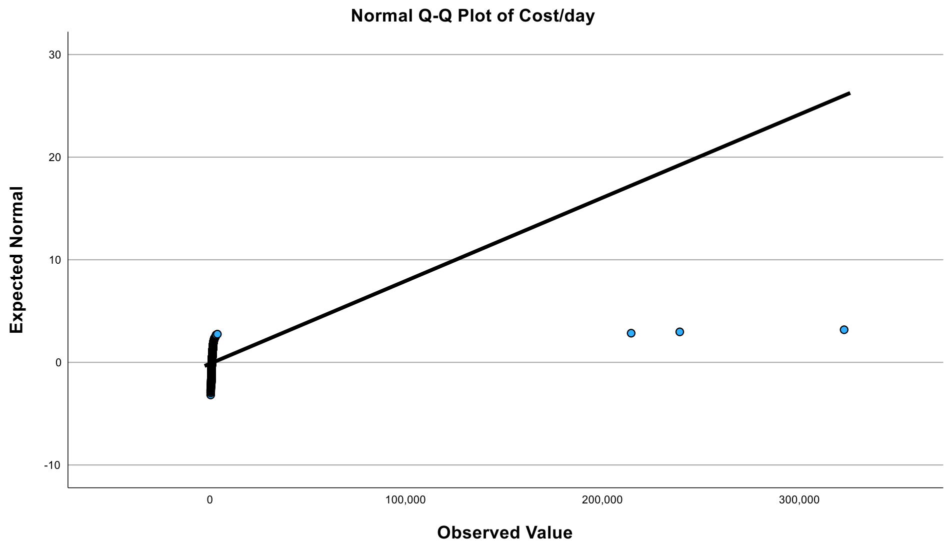

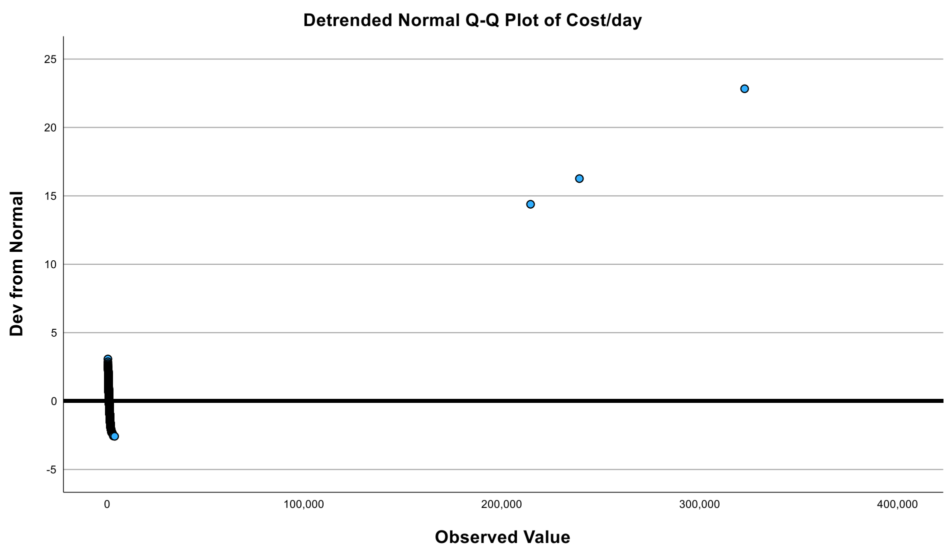

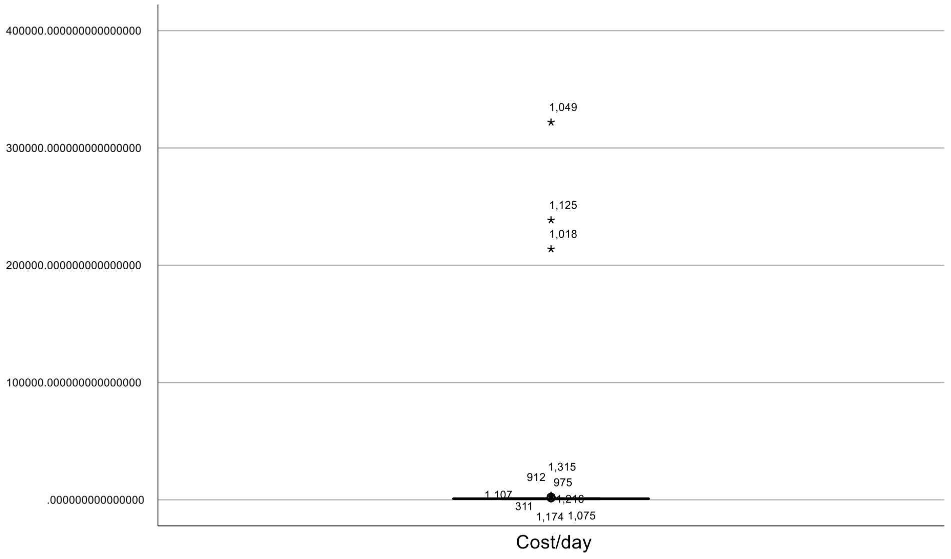

Explore ICU Y

Notes

Output Created		09-MAY-2023 11:38:44
Comments		
Input	Data	C:\Users\paho9\OneDrive\Documente\Doctorat - Stratificarea severității și predicția prognosticului în faza incipientă a Pancreatitei Acute\Registru Pancreatite Acute - BUC-API\Baza date nou\Articole\Articol cost\DB_corect_COST.sav
	Active Dataset	DataSet1
	Filter	ICU_YN = 1 (FILTER)
	Weight	<none>
	Split File	<none>
	N of Rows in Working Data File	125
Missing Value Handling	Definition of Missing	User-defined missing values for dependent variables are treated as missing.
	Cases Used	Statistics are based on cases with no missing values for any dependent variable or factor used.
Syntax		EXAMINE VARIABLES=Costday /PLOT BOXPLOT STEMLEAF HISTOGRAM NPPLOT /COMPARE GROUPS /STATISTICS DESCRIPTIVES EXTREME /CINTERVAL 95 /MISSING LISTWISE

		/NOTOTAL.
Resources	Processor Time	00:00:00.41
	Elapsed Time	00:00:00.74

Case Processing Summary

	Cases					
	Valid		Missing		Total	
	N	Percent	N	Percent	N	Percent
Cost/day	125	100.0%	0	0.0%	125	100.0%

Descriptives

			Statistic	Std. Error
Cost/day	Mean		2000.911057368718500	133.315673526523200
	95% Confidence Interval for Mean	Lower Bound	1737.041995525944000	
		Upper Bound	2264.780119211493000	
	5% Trimmed Mean		1788.935997220236500	
	Median		1376.292941176470500	
	Variance		2221633.601	
	Std. Deviation		1490.514542357375000	
	Minimum		541.335789473684100	
	Maximum		8662.000000000000000	
	Range		8120.664210526316000	
	Interquartile Range		1055.183179824561600	
	Skewness		2.359	.217
	Kurtosis		5.919	.430

Extreme Values

			Case Number	Value
Cost/day	Highest	1	1219	8662.000000000000000
		2	909	7655.230000000000000
		3	1274	7595.160000000000000
		4	702	6290.557500000000000
		5	1247	6255.410000000000000
	Lowest	1	1097	541.335789473684100
		2	350	831.953571400000000
		3	854	850.106153846153800
		4	222	889.285714285714300
		5	1013	892.834062500000000

Tests of Normality

	Kolmogorov-Smirnov[a]			Shapiro-Wilk		
	Statistic	df	Sig.	Statistic	df	Sig.
Cost/day	.242	125	<.001	.699	125	<.001

a. Lilliefors Significance Correction

Cost/day

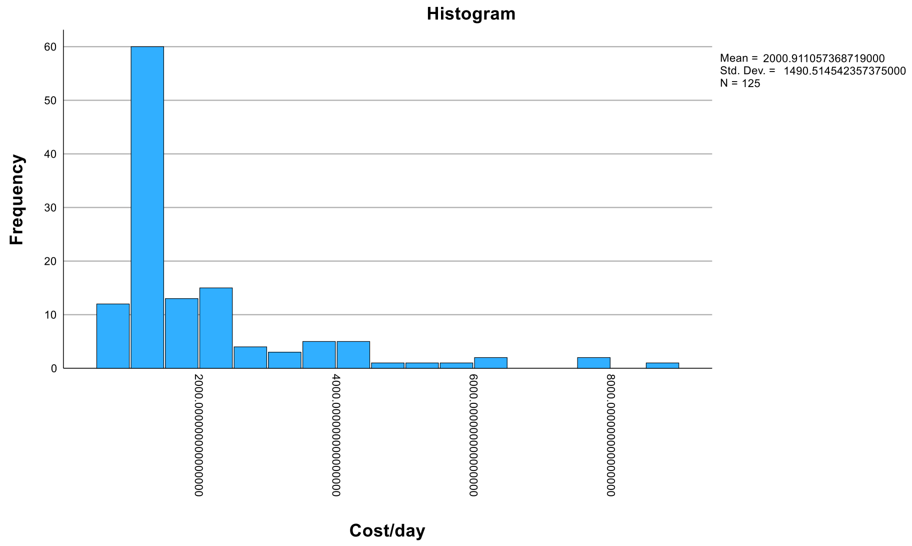


```
Cost/day Stem-and-Leaf Plot

 Frequency    Stem &  Leaf

     1.00        0 .  5
      .00        0 .
    11.00        0 .  88888899999
    27.00        1 .  000000000000011111111111111
    27.00        1 .  222222222222222223333333333
    11.00        1 .  44444455555
     8.00        1 .  66666777
      .00        1 .
     7.00        2 .  0001111
     7.00        2 .  2222233
     2.00        2 .  45
     2.00        2 .  66
     1.00        2 .  8
     3.00        3 .  001
      .00        3 .
     1.00        3 .  5
     3.00        3 .  667
    14.00 Extremes    (>=3879)

 Stem width:  1000.000
```

```
Each leaf:        1 case(s)
```

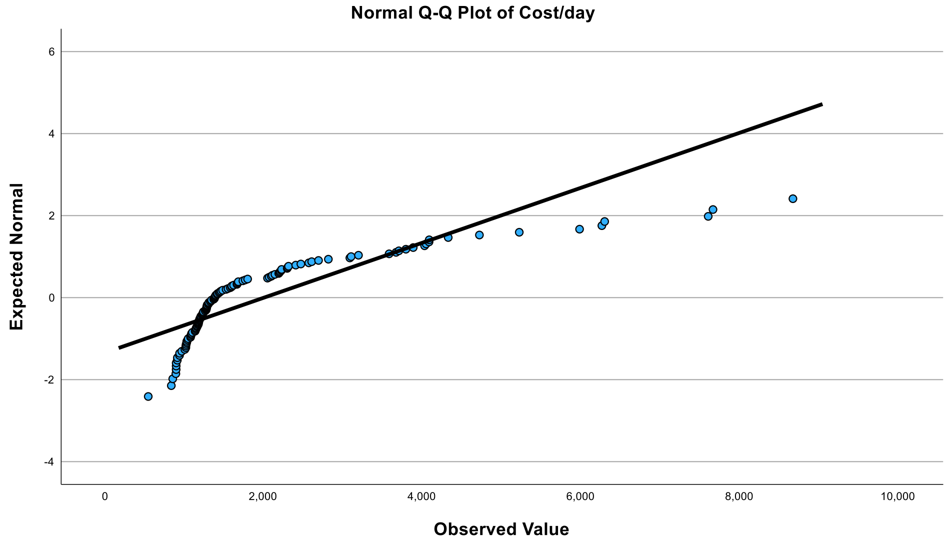

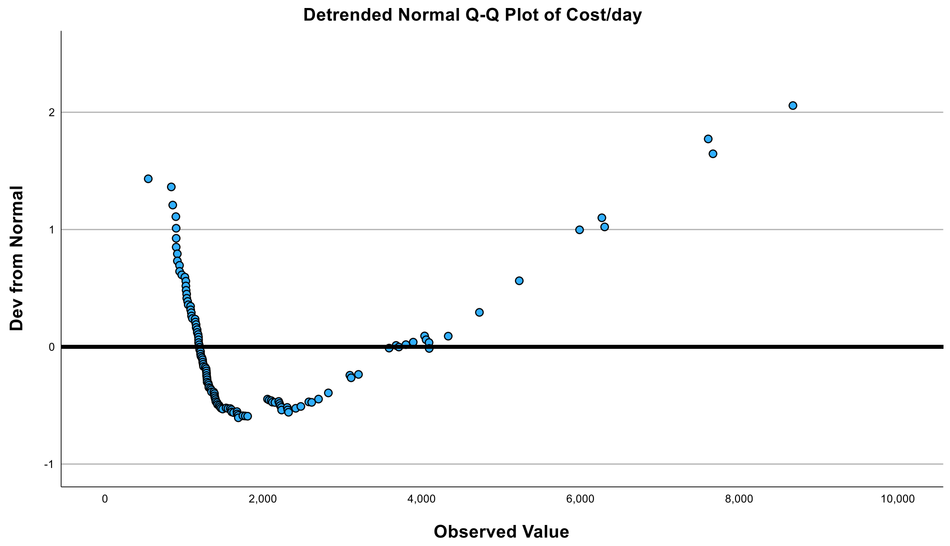

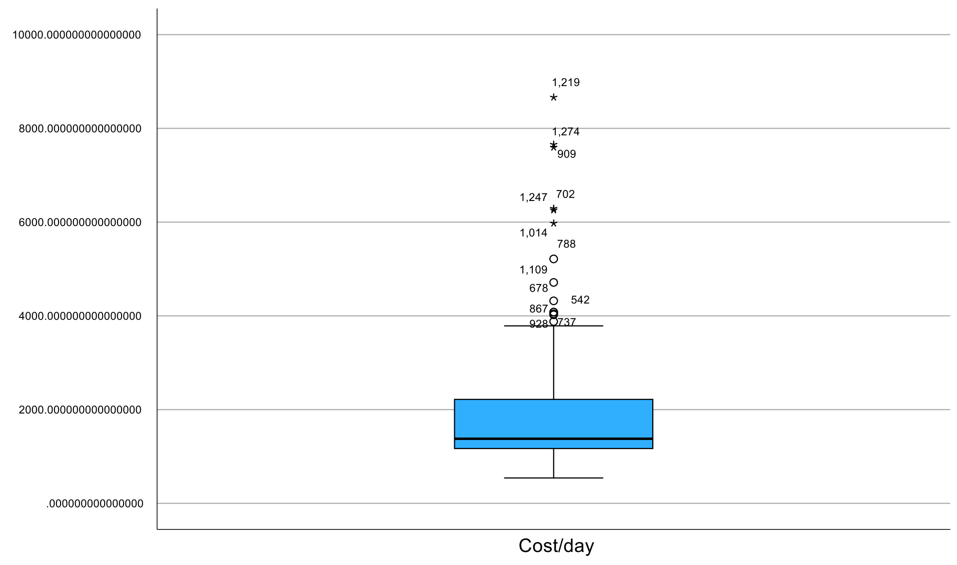

Nonparametric Tests

Notes

Output Created		09-MAY-2023 11:41:49
Comments		
Input	Data	C:\Users\paho9\OneDrive\Documente\Doctorat - Stratificarea severității și predicția prognosticului în faza incipientă a Pancreatitei Acute\Registru Pancreatite Acute - BUC-API\Baza date nou\Articole\Articol cost\DB_corect_COST.sav
	Active Dataset	DataSet1
	Filter	<none>

	Weight	<none>
	Split File	<none>
	N of Rows in Working Data File	1473
Syntax		NPTESTS /INDEPENDENT TEST (Costday) GROUP (ICU_YN) KRUSKAL_WALLIS(COMPARE=PAIRWISE) /MISSING SCOPE=ANALYSIS USERMISSING=EXCLUDE /CRITERIA ALPHA=0.05 CILEVEL=95.
Resources	Processor Time	00:00:00.37
	Elapsed Time	00:00:00.63

Hypothesis Test Summary

	Null Hypothesis	Test	Sig.[a,b]
1	The distribution of Cost/day is the same across categories of ICU_Y/N.	Independent-Samples Kruskal-Wallis Test	<.001

Hypothesis Test Summary

	Decision
1	Reject the null hypothesis.

a. The significance level is .050.

b. Asymptotic significance is displayed.

Independent-Samples Kruskal-Wallis Test

Cost/day across ICU_Y/N

Independent-Samples Kruskal-Wallis Test Summary

Total N	1473
Test Statistic	187.586[a,b]
Degree Of Freedom	1
Asymptotic Sig.(2-sided test)	<.001

a. The test statistic is adjusted for ties.

b. Multiple comparisons are not performed because there are less than three test fields.

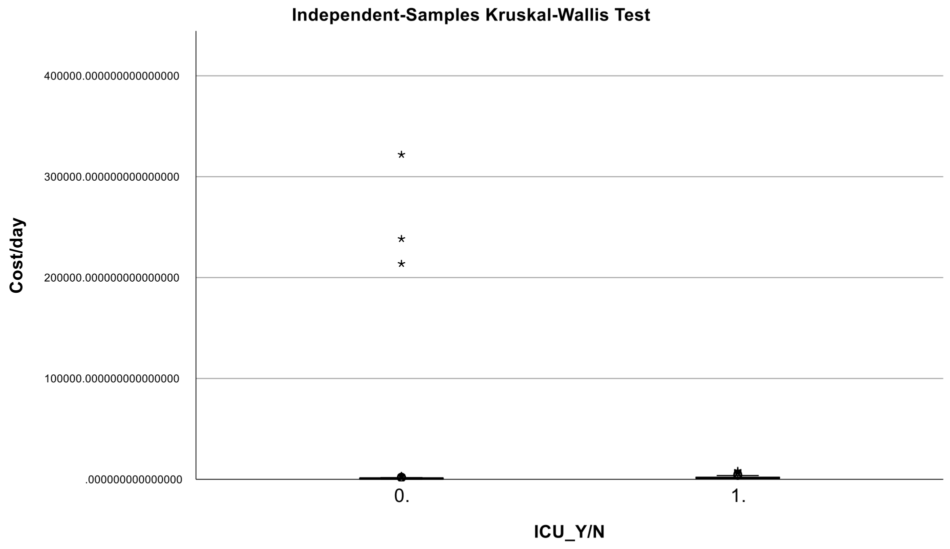

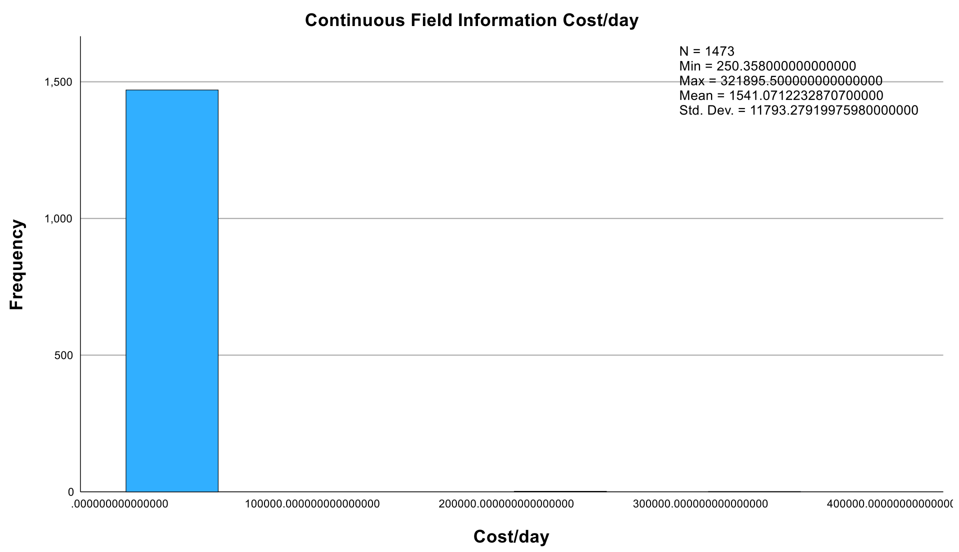

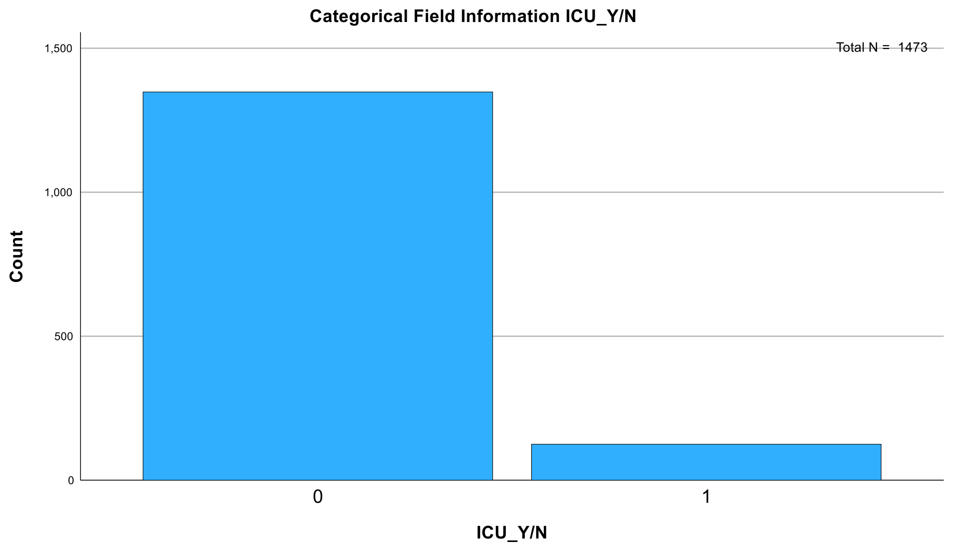

Explore MALES

Notes

Output Created		09-MAY-2023 11:44:54
Comments		
Input	Data	C:\Users\paho9\OneDrive\Documente\Doctorat - Stratificarea severității și predicția prognosticului în faza incipientă a Pancreatitei Acute\Registru Pancreatite Acute - BUC-API\Baza date nou\Articole\Articol cost\DB_corect_COST.sav
	Active Dataset	DataSet1
	Filter	Sex = 1 (FILTER)
	Weight	<none>
	Split File	<none>
	N of Rows in Working Data File	911
Missing Value Handling	Definition of Missing	User-defined missing values for dependent variables are treated as missing.
	Cases Used	Statistics are based on cases with no missing values for any dependent variable or factor used.
Syntax		EXAMINE VARIABLES=Costday /PLOT BOXPLOT STEMLEAF HISTOGRAM NPPLOT /COMPARE GROUPS /STATISTICS DESCRIPTIVES EXTREME /CINTERVAL 95 /MISSING LISTWISE

		/NOTOTAL.
Resources	Processor Time	00:00:00.45
	Elapsed Time	00:00:00.83

Case Processing Summary

	Cases					
	Valid		Missing		Total	
	N	Percent	N	Percent	N	Percent
Cost/day	911	100.0%	0	0.0%	911	100.0%

Descriptives

			Statistic	Std. Error
Cost/day	Mean		970.95947124 6977400	18.818306727 160320
	95% Confidence Interval for Mean	Lower Bound	934.02714632 4353200	
		Upper Bound	1007.8917961 69601400	
	5% Trimmed Mean		898.86558656 6277300	
	Median		840.74000000 0000000	
	Variance		322611.217	
	Std. Deviation		567.98874691 1935800	
	Minimum		250.35800000 0000000	
	Maximum		7595.1600000 00000000	
	Range		7344.8020000 00000000	
	Interquartile Range		427.81173333 3333340	
	Skewness		5.286	.081
	Kurtosis		42.273	.162

Extreme Values

			Case Number	Value
Cost/day	Highest	1	1274	7595.160000000000000
		2	702	6290.557500000000000
		3	1014	5974.465000000000000
		4	1109	4713.045000000000000
		5	678	4320.065000000000000
	Lowest	1	492	250.358000000000000
		2	206	252.000000000000000
		3	257	277.444444444444460
		4	464	278.723333333333300
		5	232	291.500000000000000

Tests of Normality

	Kolmogorov-Smirnov[a]			Shapiro-Wilk		
	Statistic	df	Sig.	Statistic	df	Sig.
Cost/day	.191	911	<.001	.583	911	<.001

a. Lilliefors Significance Correction

Cost/day

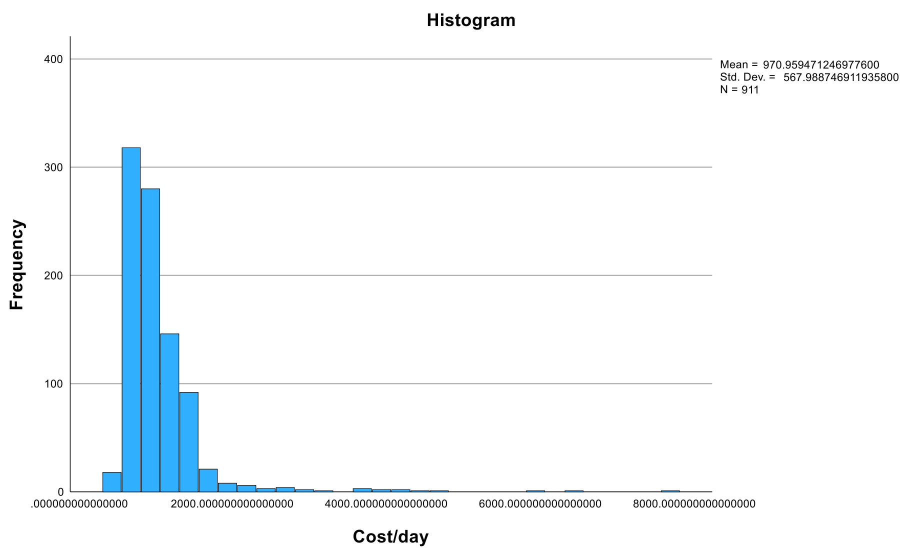


```
Cost/day Stem-and-Leaf Plot

 Frequency    Stem &  Leaf

     5.00        2 .  57&
     4.00        3 .  2&
     9.00        4 .  499&
    75.00        5 .  0223333444555566666677778888999999&
   168.00        6 .  0000001111111222222222223333333333444444444555555555666666666677777777788888899999999
   147.00        7 .  000000011111112222222223333444444444455555555566666666677777788888899999
   113.00        8 .  0000111111222222233334444444455555555666677777788889999
    95.00        9 .  0000011222222333444555556666666777788888899999
    61.00       10 .  0000111222233444677888889999&
    66.00       11 .  000012233333444555566666777778899
    52.00       12 .  001133345566777788899999&
    39.00       13 .  000002223345677889&
    20.00       14 .  0013458&&
    12.00       15 .  1139&
     7.00       16 .  27&
     2.00       17 .  &
    36.00 Extremes    (>=1770)

 Stem width:  100.0000
 Each leaf:        2 case(s)
```

& denotes fractional leaves.

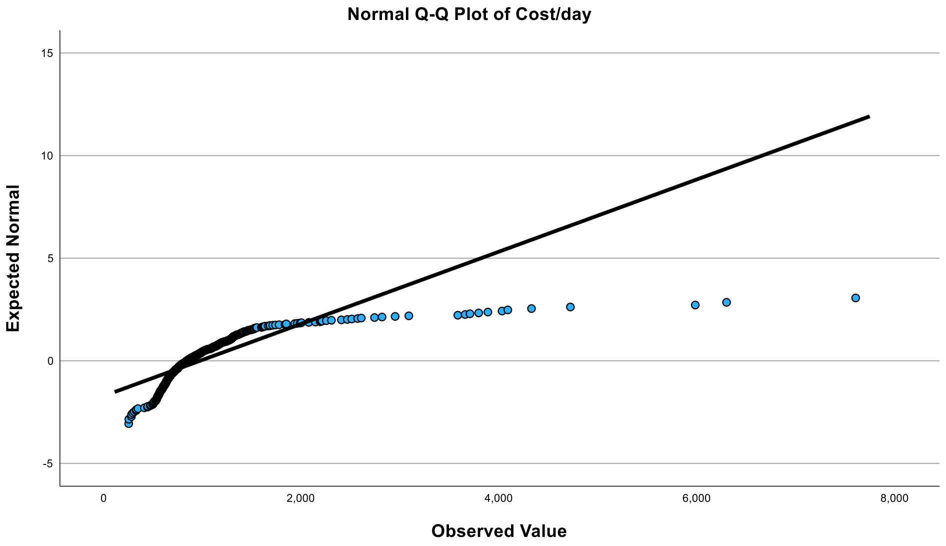

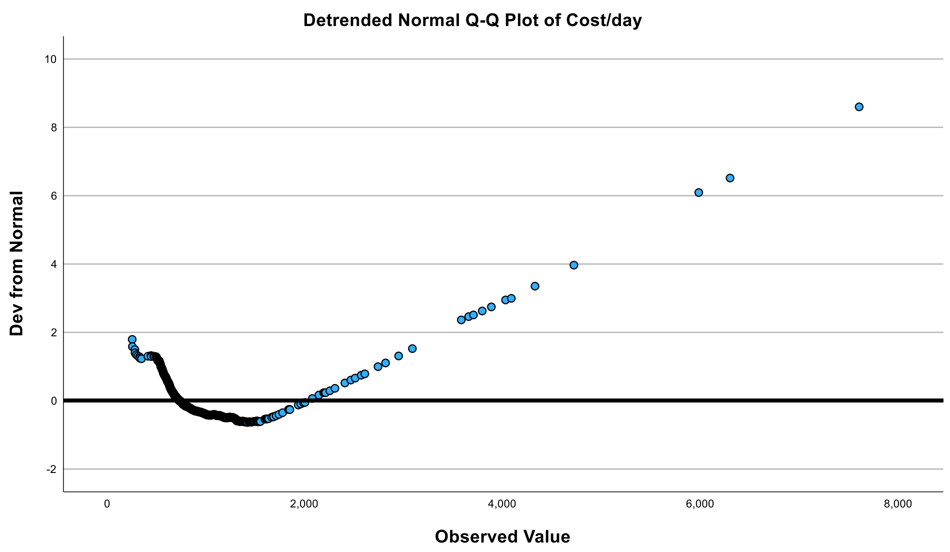

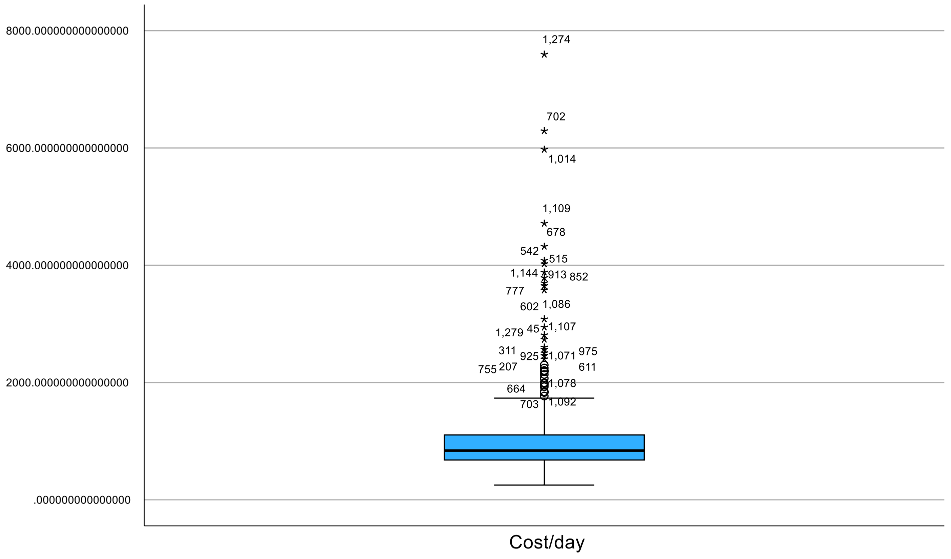

Explore FEMALES

Notes

Output Created		09-MAY-2023 11:45:20
Comments		
Input	Data	C:\Users\paho9\OneDrive\Documente\Doctorat - Stratificarea severității și predicția prognosticului în faza incipientă a Pancreatitei Acute\Registru Pancreatite Acute - BUC-API\Baza date nou\Articole\Articol cost\DB_corect_COST.sav
	Active Dataset	DataSet1
	Filter	Sex = 2 (FILTER)
	Weight	<none>
	Split File	<none>
	N of Rows in Working Data File	562
Missing Value Handling	Definition of Missing	User-defined missing values for dependent variables are treated as missing.
	Cases Used	Statistics are based on cases with no missing values for any dependent variable or factor used.
Syntax		EXAMINE VARIABLES=Costday /PLOT BOXPLOT STEMLEAF HISTOGRAM NPPLOT /COMPARE GROUPS /STATISTICS DESCRIPTIVES EXTREME /CINTERVAL 95 /MISSING LISTWISE

		/NOTOTAL.
Resources	Processor Time	00:00:00.37
	Elapsed Time	00:00:00.73

Case Processing Summary

	Cases					
	Valid		Missing		Total	
	N	Percent	N	Percent	N	Percent
Cost/day	562	100.0%	0	0.0%	562	100.0%

Descriptives

			Statistic	Std. Error
Cost/day	Mean		2465.220344476620000	803.713718617385800
	95% Confidence Interval for Mean	Lower Bound	886.564560565809400	
		Upper Bound	4043.876128387432000	
	5% Trimmed Mean		1013.529859784099500	
	Median		993.845833333333300	
	Variance		363027126.720	
	Std. Deviation		9223.372036854777000	
	Minimum		346.000000000000000	
	Maximum		9223.372036854777000	
	Range		9223.372036854777000	
	Interquartile Range		403.286604554865330	
	Skewness		14.267	.103
	Kurtosis		208.952	.206

Extreme Values

			Case Number	Value
Cost/day	Highest	1	1049	9223.372036854777000
		2	1125	9223.372036854777000
		3	1018	9223.372036854777000
		4	1219	8662.000000000000000
		5	909	7655.230000000000000
	Lowest	1	47	346.000000000000000
		2	1473	355.864285714285760
		3	802	371.770000000000000
		4	251	388.500000000000000
		5	543	493.481111111111100

Tests of Normality

	Kolmogorov-Smirnov[a]			Shapiro-Wilk		
	Statistic	df	Sig.	Statistic	df	Sig.
Cost/day	.473	562	<.001	.055	562	<.001

a. Lilliefors Significance Correction

Cost/day

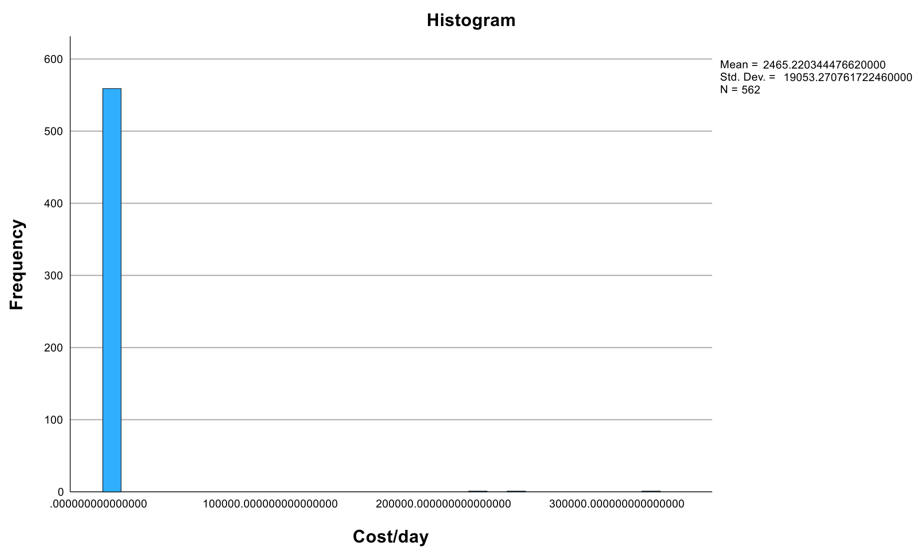


```
Cost/day Stem-and-Leaf Plot

 Frequency    Stem &  Leaf

     4.00        3 .  4578
     1.00        4 .  9
    14.00        5 .  56667777889999
    58.00        6 .  0011111222233333333444445555555555666667777778888999999999
    67.00        7 .  0000001111112222233333333333334444444555555555556666777888888888999
    61.00        8 .  0000000111111223333333334444444444555556666666667778999999999
    84.00        9 .  000000111223333333333333333344444444444555555556666666667777777788888888889999999999999
    89.00       10 .  00000001111111111111112222222222333334444444444455555556666666667777777777777778888999999999
    47.00       11 .  00000000111112222233344444555555666777888899999
    47.00       12 .  0000001111222222222333444444555666677777777788889
    22.00       13 .  0111122233445566778889
    15.00       14 .  334445566667789
    11.00       15 .  01125777889
     8.00       16 .  14567778
     5.00       17 .  02269
    29.00 Extremes    (>=1999)

 Stem width:  100.0000
 Each leaf:        1 case(s)
```

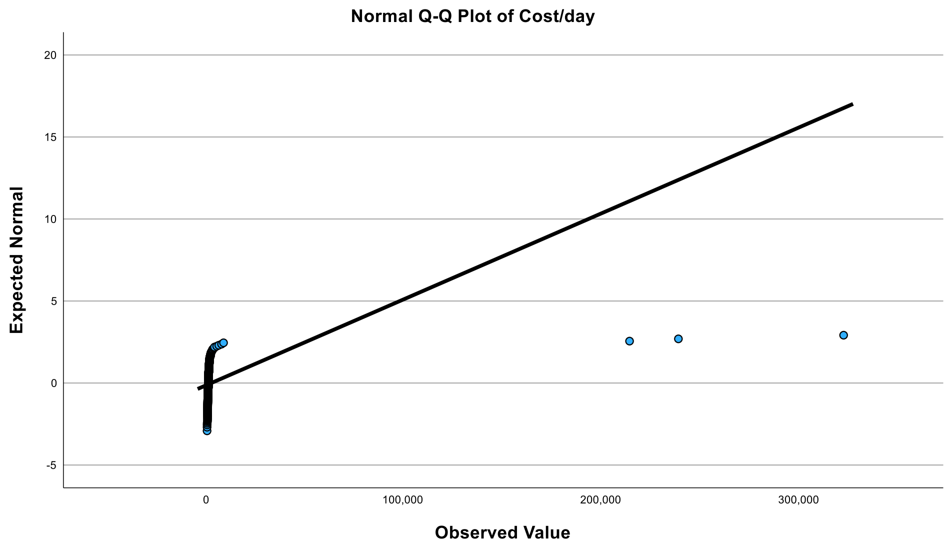
Normal Q-Q Plot of Cost/day
Expected Normal
Observed Value
20
15
10
5
0
-5
0
100,000
200,000
300,000

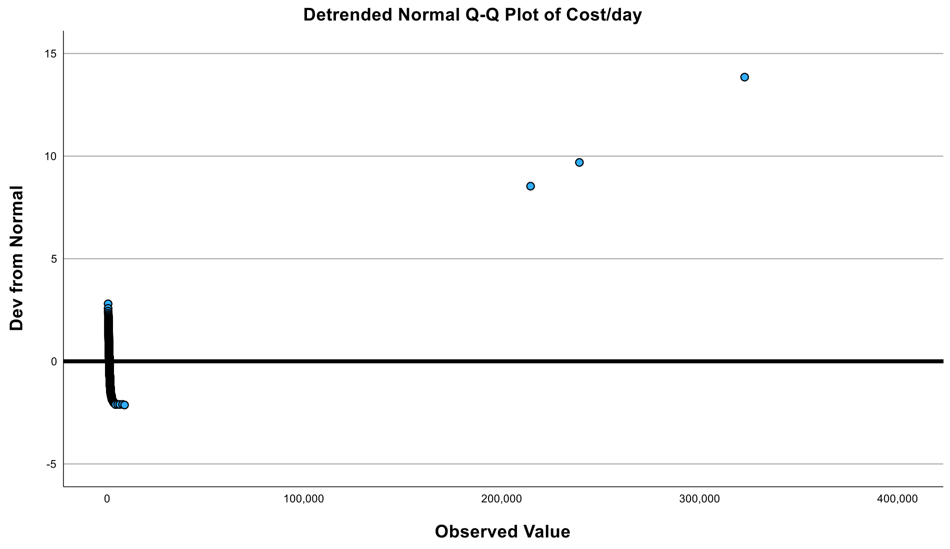
Detrended Normal Q-Q Plot of Cost/day
Dev from Normal
Observed Value
15
10
5
0
-5
0
100,000
200,000
300,000
400,000

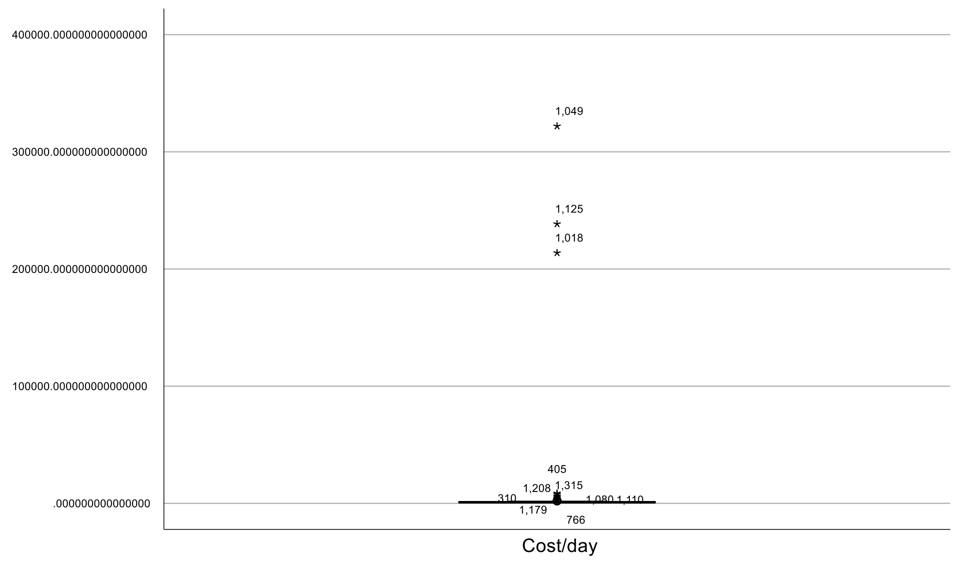

Nonparametric Tests

Notes

Output Created		09-MAY-2023 11:47:07
Comments		
Input	Data	C:\Users\paho9\OneDrive\Documente\Doctorat - Stratificarea severității și predicția prognosticului în faza incipientă a Pancreatitei Acute\Registru Pancreatite Acute - BUC-API\Baza date nou\Articole\Articol cost\DB_corect_COST.sav
	Active Dataset	DataSet1
	Filter	<none>

	Weight	<none>
	Split File	<none>
	N of Rows in Working Data File	1473
Syntax		NPTESTS /INDEPENDENT TEST (Costday) GROUP (Sex) KRUSKAL_WALLIS(COMPARE=PAIRWISE) /MISSING SCOPE=ANALYSIS USERMISSING=EXCLUDE /CRITERIA ALPHA=0.05 CILEVEL=95.
Resources	Processor Time	00:00:00.37
	Elapsed Time	00:00:00.63

Hypothesis Test Summary

	Null Hypothesis	Test	Sig.[a,b]
1	The distribution of Cost/day is the same across categories of Sex.	Independent-Samples Kruskal-Wallis Test	<.001

Hypothesis Test Summary

	Decision
1	Reject the null hypothesis.

a. The significance level is .050.

b. Asymptotic significance is displayed.

Independent-Samples Kruskal-Wallis Test

Cost/day across Sex
(1 – Male / 2 – Female)

Independent-Samples Kruskal-Wallis Test Summary

Total N	1473
Test Statistic	54.529[a,b]
Degree Of Freedom	1
Asymptotic Sig.(2-sided test)	<.001

a. The test statistic is adjusted for ties.

b. Multiple comparisons are not performed because there are less than three test fields.

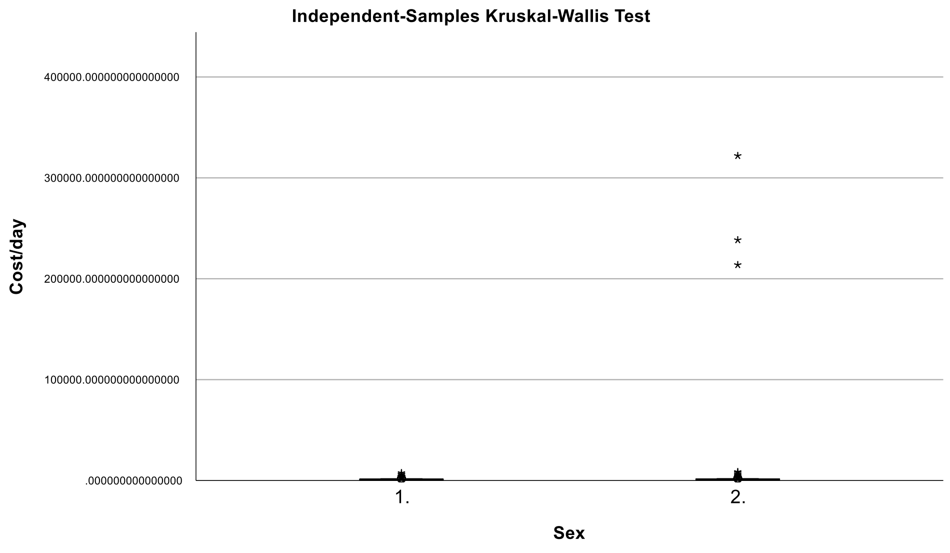

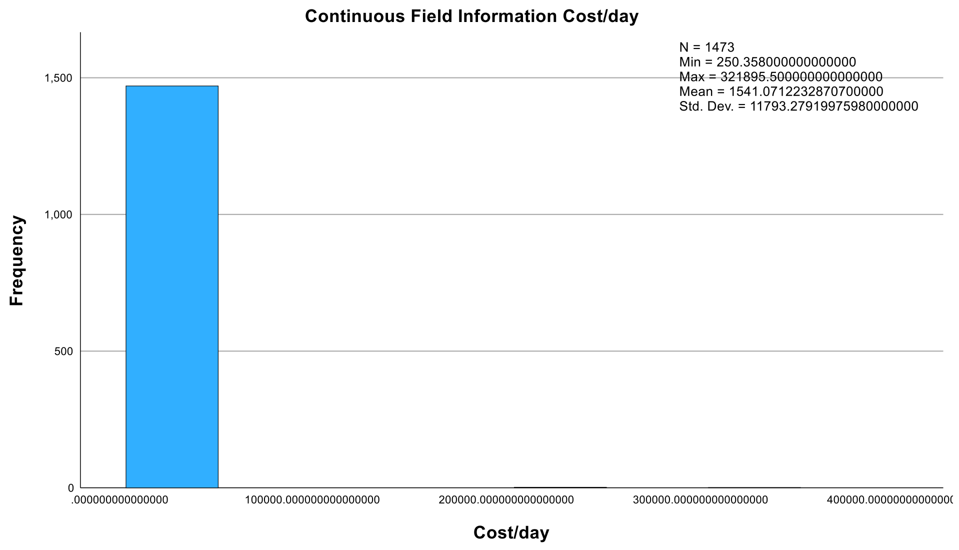

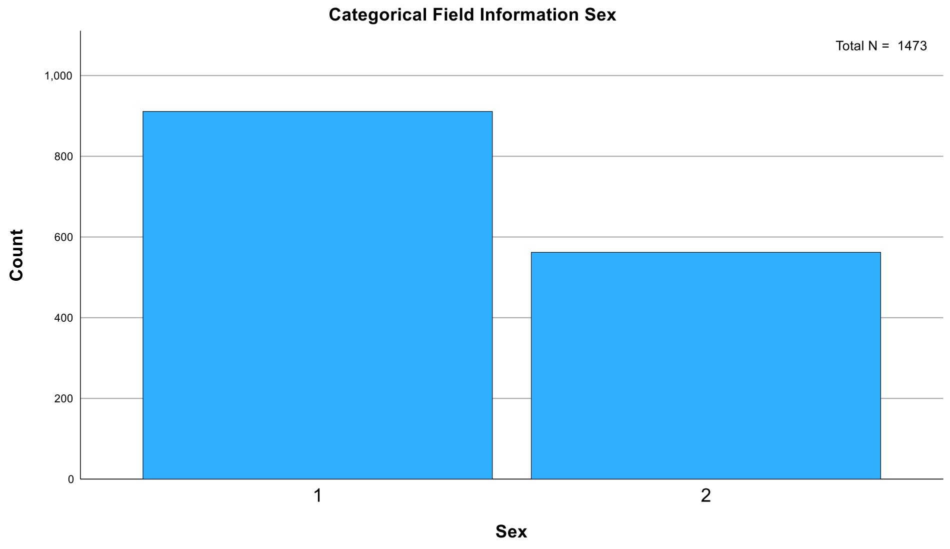

Explore GASTROENTEROLOGY

Notes

Output Created		09-MAY-2023 11:50:45
Comments		
Input	Data	C:\Users\paho9\OneDrive\Documente\Doctorat - Stratificarea severității și predicția prognosticului în faza incipientă a Pancreatitei Acute\Registru Pancreatite Acute - BUC-API\Baza date nou\Articole\Articol cost\DB_corect_COST.sav
	Active Dataset	DataSet1
	Filter	GastroChir = 1 (FILTER)
	Weight	<none>
	Split File	<none>
	N of Rows in Working Data File	860
Missing Value Handling	Definition of Missing	User-defined missing values for dependent variables are treated as missing.
	Cases Used	Statistics are based on cases with no missing values for any dependent variable or factor used.
Syntax		EXAMINE VARIABLES=Costday /PLOT BOXPLOT STEMLEAF HISTOGRAM NPPLOT /COMPARE GROUPS /STATISTICS DESCRIPTIVES EXTREME /CINTERVAL 95

		/MISSING LISTWISE<br>/NOTOTAL.
Resources	Processor Time	00:00:00.53
	Elapsed Time	00:00:00.81

Case Processing Summary

	Cases					
	Valid		Missing		Total	
	N	Percent	N	Percent	N	Percent
Cost/day	860	100.0%	0	0.0%	860	100.0%

Descriptives

			Statistic	Std. Error
Cost/day	Mean		814.080728285132200	12.251182678337099
	95% Confidence Interval for Mean	Lower Bound	790.034970879037900	
		Upper Bound	838.126485691226500	
	5% Trimmed Mean		766.352522545794600	
	Median		745.479000000000000	
	Variance		129078.670	
	Std. Deviation		359.275201253118440	
	Minimum		250.358000000000000	
	Maximum		4080.590000000000000	
	Range		3830.232000000000000	
	Interquartile Range		209.171969696969770	
	Skewness		4.706	.083
	Kurtosis		30.145	.167

Extreme Values

			Case Number	Value
Cost/day	Highest	1	515	4080.590000000000000
		2	542	4022.368571428571600
		3	370	3663.000000000000000
		4	408	3189.595000000000000
		5	1368	3097.000000000000000
	Lowest	1	492	250.358000000000000
		2	206	252.000000000000000
		3	257	277.444444444444460
		4	464	278.723333333333300
		5	232	291.500000000000000

Tests of Normality

	Kolmogorov-Smirnov[a]			Shapiro-Wilk		
	Statistic	df	Sig.	Statistic	df	Sig.
Cost/day	.215	860	<.001	.578	860	<.001

a. Lilliefors Significance Correction

Cost/day

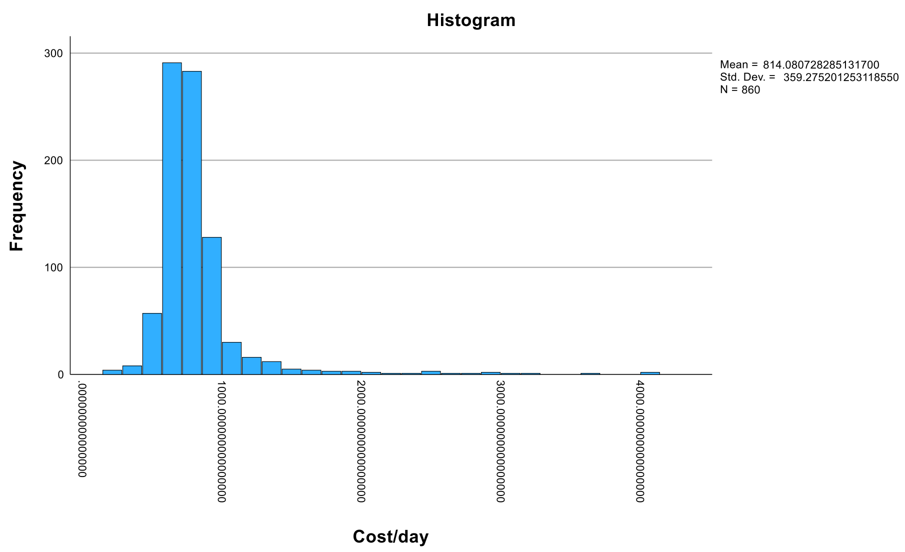


```
Cost/day Stem-and-Leaf Plot

 Frequency    Stem &  Leaf

     7.00 Extremes    (=<328)
     2.00        3 .  4
     2.00        3 .  &
     3.00        4 .  4&
     7.00        4 .  99&
    21.00        5 .  022333344&
    65.00        5 .  5555566666667777778888899999999
   109.00        6 .  0000000111111112222222222222233333333333333444444444
   117.00        6 .  55555555555555666666666666777777777777788888888999999999999
   110.00        7 .  0000000000111111111111222222222222333333333444444444444
   100.00        7 .  55555555555555566666666667777777788888888888999999
    86.00        8 .  000000011111111222222223333333344444444444
    65.00        8 .  5555555556666666677777778888899999
    38.00        9 .  000001122222333344 4
    39.00        9 .  555556666777888899
    13.00       10 .  00012&
     8.00       10 .  677&
    11.00       11 .  0234&
     4.00       11 .  55
    53.00 Extremes    (>=1191)
```

```
Stem width:  100.0000
Each leaf:         2 case(s)

& denotes fractional leaves.
```

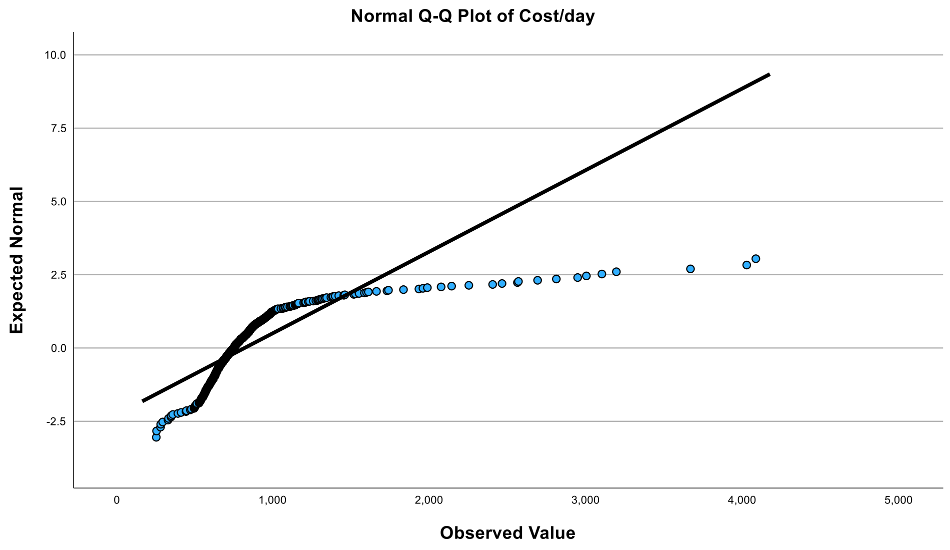

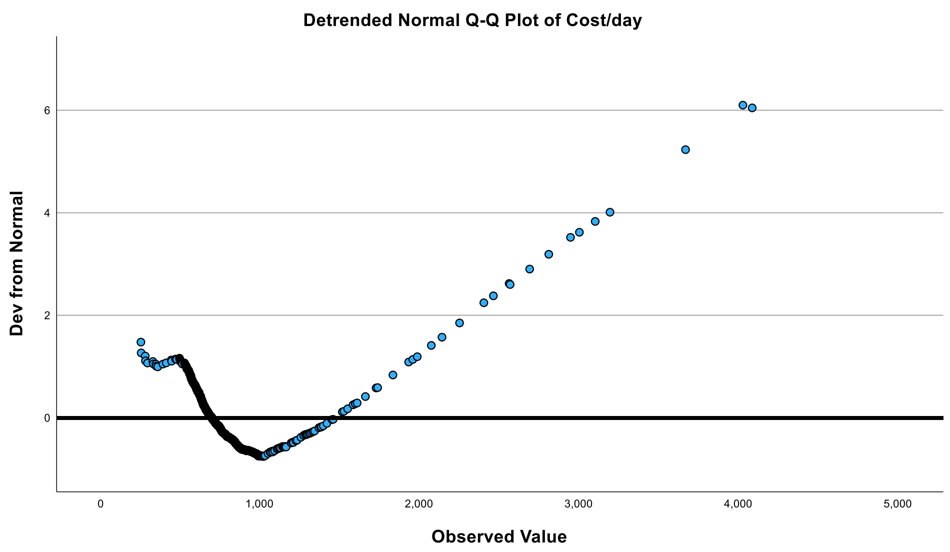

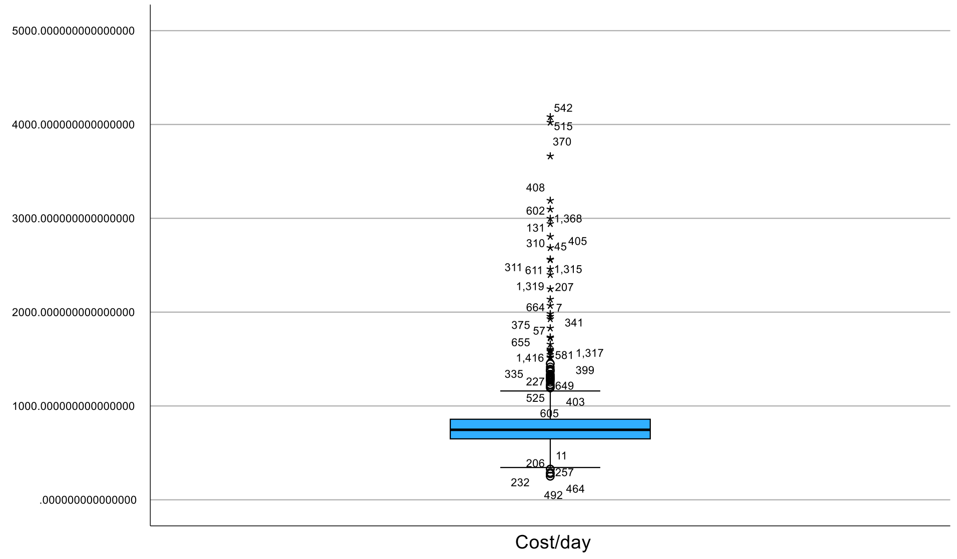

Explore SURGERY

Notes

Output Created		09-MAY-2023 11:51:16
Comments		
Input	Data	C:\Users\paho9\OneDrive\Documente\Doctorat - Stratificarea severității și predicția prognosticului în faza incipientă a Pancreatitei Acute\Registru Pancreatite Acute - BUC-API\Baza date nou\Articole\Articol cost\DB_corect_COST.sav
	Active Dataset	DataSet1
	Filter	GastroChir = 2 (FILTER)
	Weight	<none>
	Split File	<none>
	N of Rows in Working Data File	613
Missing Value Handling	Definition of Missing	User-defined missing values for dependent variables are treated as missing.
	Cases Used	Statistics are based on cases with no missing values for any dependent variable or factor used.
Syntax		EXAMINE VARIABLES=Costday /PLOT BOXPLOT STEMLEAF HISTOGRAM NPPLOT /COMPARE GROUPS /STATISTICS DESCRIPTIVES EXTREME /CINTERVAL 95 /MISSING LISTWISE

		/NOTOTAL.
Resources	Processor Time	00:00:00.44
	Elapsed Time	00:00:00.76

Case Processing Summary

	Cases					
	Valid		Missing		Total	
	N	Percent	N	Percent	N	Percent
Cost/day	613	100.0%	0	0.0%	613	100.0%

Descriptives

			Statistic	Std. Error
Cost/day	Mean		2560.9926355 24704000	736.55082937 2172300
	95% Confidence Interval for Mean	Lower Bound	1114.5189157 81567300	
		Upper Bound	4007.4663552 67840300	
	5% Trimmed Mean		1195.1076622 45426400	
	Median		1142.5641666 66666800	
	Variance		332556867.16 5	
	Std. Deviation		9223.3720368 54777000	
	Minimum		306.45266666 6666700	
	Maximum		9223.3720368 54777000	
	Range		9223.3720368 54777000	
	Interquartile Range		304.38767857 1428500	
	Skewness		14.896	.099
	Kurtosis		228.005	.197

Extreme Values

			Case Number	Value
Cost/day	Highest	1	1049	9223.372036854777000
		2	1125	9223.372036854777000
		3	1018	9223.372036854777000
		4	1219	8662.000000000000000
		5	909	7655.230000000000000
	Lowest	1	916	306.452666666666700
		2	802	371.770000000000000
		3	752	533.016666666666700
		4	1097	541.335789473684100
		5	1200	575.363636363636400

Tests of Normality

	Kolmogorov-Smirnov[a]			Shapiro-Wilk		
	Statistic	df	Sig.	Statistic	df	Sig.
Cost/day	.465	613	<.001	.053	613	<.001

a. Lilliefors Significance Correction

Cost/day

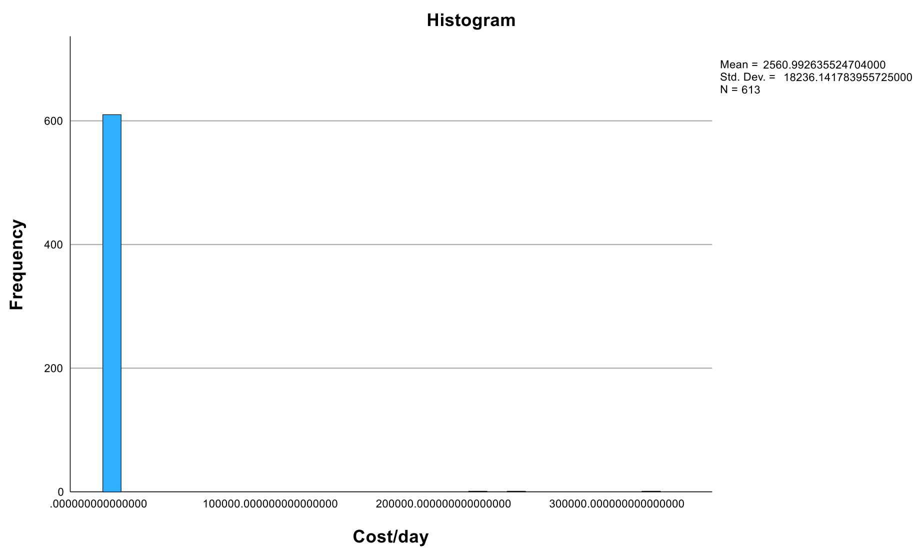


```
Cost/day Stem-and-Leaf Plot

 Frequency    Stem &  Leaf

     4.00 Extremes    (=<541)
     1.00        5 .  &
      .00        6 .
     4.00        7 .  &&
    23.00        8 .  2455679999&
   102.00        9 .  0001222333333444444555566666677777888889999999999
   129.00       10 .  000001111111122222223333344444445555666677777777888889999999999
    96.00       11 .  000000011122233333344445555566666777778888999
    89.00       12 .  0000111122233333444455566666777777788899999
    52.00       13 .  000011222233445566778889
    32.00       14 .  0013344566789&
    18.00       15 .  0111379&
    13.00       16 .  1277&&
     5.00       17 .  06&
    45.00 Extremes    (>=1793)

 Stem width:  100.0000
 Each leaf:        2 case(s)
```

& denotes fractional leaves.

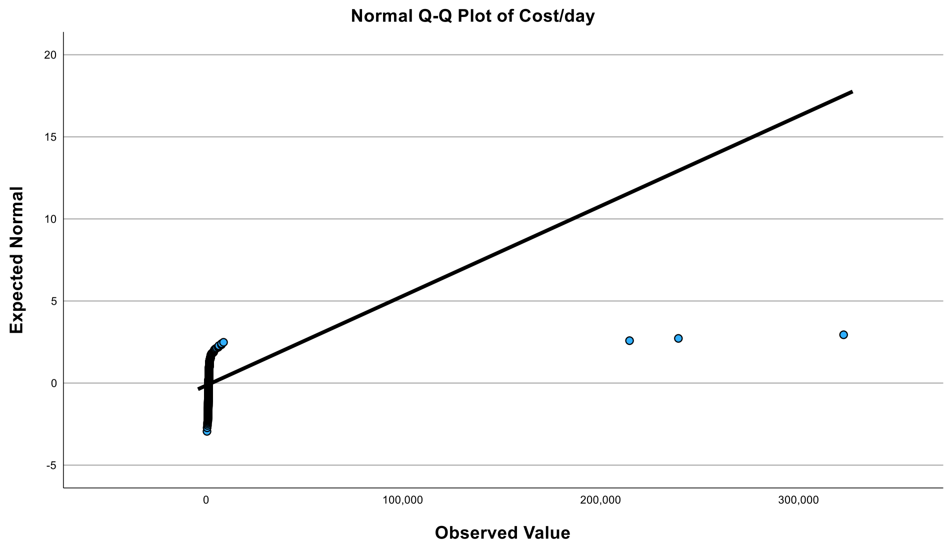

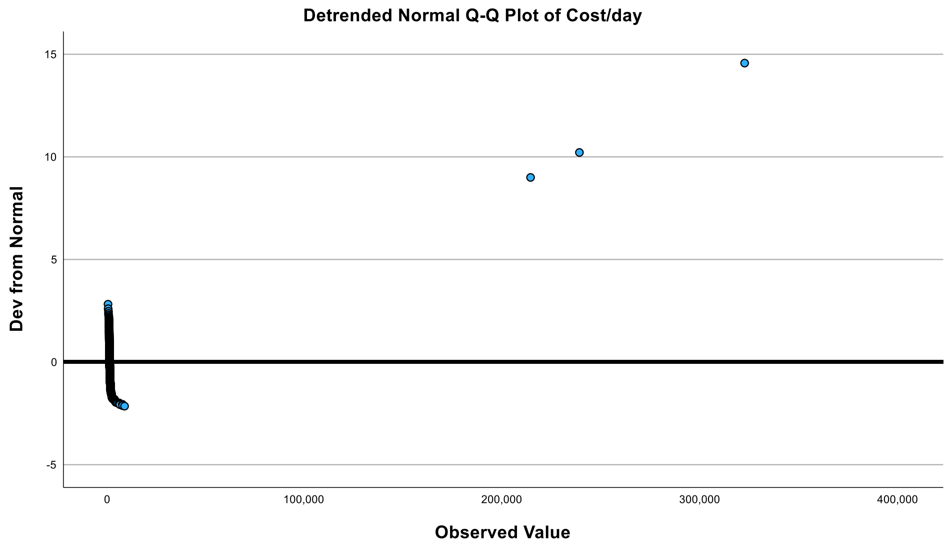

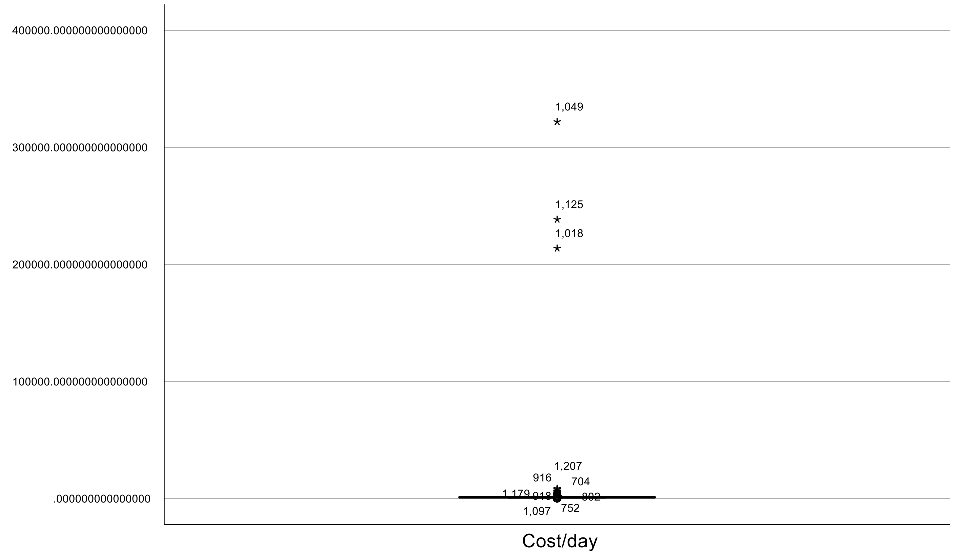

Nonparametric Tests

Notes

Output Created		09-MAY-2023 11:53:43
Comments		
Input	Data	C:\Users\paho9\OneDrive\Documente\Doctorat - Stratificarea severității și predicția prognosticului în faza incipientă a Pancreatitei Acute\Registru Pancreatite Acute - BUC-API\Baza date nou\Articole\Articol cost\DB_corect_COST.sav
	Active Dataset	DataSet1

	Filter	<none>
	Weight	<none>
	Split File	<none>
	N of Rows in Working Data File	1473
Syntax		NPTESTS /INDEPENDENT TEST (Costday) GROUP (GastroChir) KRUSKAL_WALLIS(COMPARE=PAIRWISE) /MISSING SCOPE=ANALYSIS USERMISSING=EXCLUDE /CRITERIA ALPHA=0.05 CILEVEL=95.
Resources	Processor Time	00:00:00.41
	Elapsed Time	00:00:00.63

Hypothesis Test Summary

	Null Hypothesis	Test	Sig.[a,b]
1	The distribution of Cost/day is the same across categories of Gastro/Chir.	Independent-Samples Kruskal-Wallis Test	<.001

Hypothesis Test Summary

	Decision
1	Reject the null hypothesis.

a. The significance level is .050.

b. Asymptotic significance is displayed.

Independent-Samples Kruskal-Wallis Test

Cost/day across Gastro/Chir

(1 – GASTROENTEROLOGY / 2 – SURGERY)

Independent-Samples Kruskal-Wallis Test Summary

Total N	1473
Test Statistic	728.612[a,b]
Degree Of Freedom	1
Asymptotic Sig.(2-sided test)	<.001

a. The test statistic is adjusted for ties.

b. Multiple comparisons are not performed because there are less than three test fields.

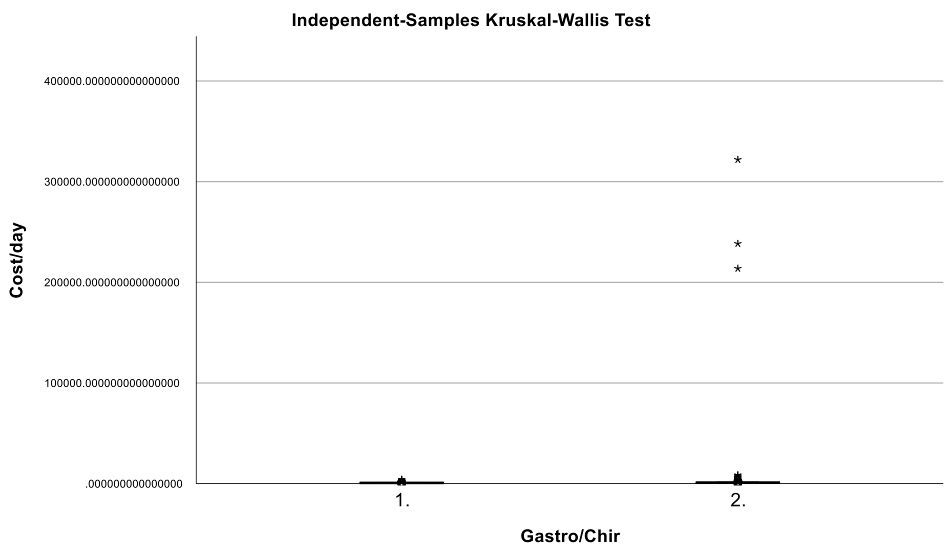

Independent-Samples Kruskal-Wallis Test
Cost/day
400000.0000000000000000
300000.0000000000000000
200000.0000000000000000
100000.0000000000000000
.0000000000000000
1.
2.
Gastro/Chir

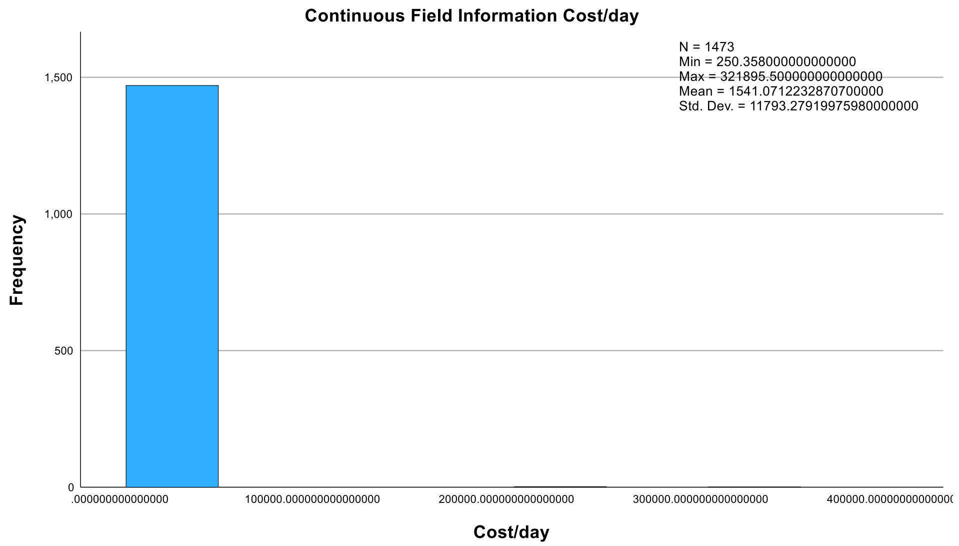

Continuous Field Information Cost/day
N = 1473
Min = 250.358000000000000
Max = 321895.500000000000000
Mean = 1541.0712232870700000
Std. Dev. = 11793.27919975980000000
Frequency
1,500
1,000
500
0
.0000000000000000
100000.0000000000000000
200000.0000000000000000
300000.0000000000000000
Cost/day

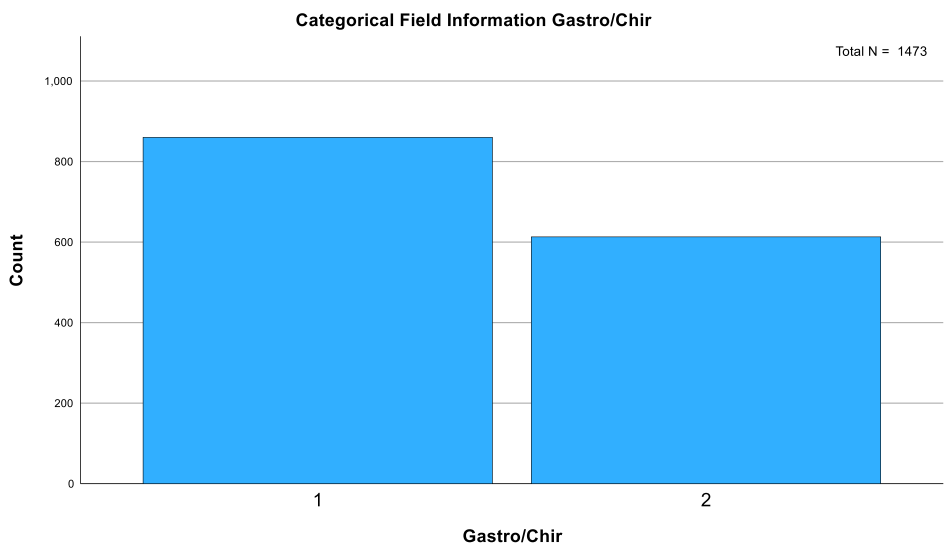

Correlations AGE

Notes

Output Created		09-MAY-2023 12:24:49
Comments		
Input	Active Dataset	DataSet1
	Filter	<none>
	Weight	<none>
	Split File	<none>
	N of Rows in Working Data File	1473
Missing Value Handling	Definition of Missing	User-defined missing values are treated as missing.
	Cases Used	Statistics for each pair of variables are based on all the cases with valid data for that pair.
Syntax		CORRELATIONS /VARIABLES=Age

		Costday /PRINT=TWOTAIL NOSIG FULL /STATISTICS DESCRIPTIVES /MISSING=PAIRWISE.
Resources	Processor Time	00:00:00.02
	Elapsed Time	00:00:00.04

Descriptive Statistics

	Mean	Std. Deviation	N
Age	56.46	16.849	1473
Cost/day	1541.0712232 87070800	9223.3720368 54777000	1473

Correlations

		Age	Cost/day
Age	Pearson Correlation	1	.062*
	Sig. (2-tailed)		.017
	N	1473	1473
Cost/day	Pearson Correlation	.062*	1
	Sig. (2-tailed)	.017	
	N	1473	1473

*. Correlation is significant at the 0.05 level (2-tailed).

Correlations LoS

Notes

Output Created		09-MAY-2023 12:26:09
Comments		
Input	Active Dataset	DataSet1
	Filter	<none>

	Weight	<none>
	Split File	<none>
	N of Rows in Working Data File	1473
Missing Value Handling	Definition of Missing	User-defined missing values are treated as missing.
	Cases Used	Statistics for each pair of variables are based on all the cases with valid data for that pair.
Syntax		CORRELATIONS /VARIABLES=Costday DoA /PRINT=TWOTAIL NOSIG FULL /STATISTICS DESCRIPTIVES /MISSING=PAIRWISE.
Resources	Processor Time	00:00:00.03
	Elapsed Time	00:00:00.04

Descriptive Statistics

	Mean	Std. Deviation	N
Cost/day	1541.0712232 87070800	9223.3720368 54777000	1473
DoA	8.70	7.781	1473

Correlations

		Cost/day	DoA
Cost/day	Pearson Correlation	1	-.029
	Sig. (2-tailed)		.260
	N	1473	1473
DoA	Pearson Correlation	-.029	1
	Sig. (2-tailed)	.260	
	N	1473	1473

Frequencies

Notes

Output Created		09-MAY-2023 12:47:46
Comments		
Input	Active Dataset	DataSet1
	Filter	<none>
	Weight	<none>
	Split File	<none>
	N of Rows in Working Data File	1473
Missing Value Handling	Definition of Missing	User-defined missing values are treated as missing.
	Cases Used	Statistics are based on all cases with valid data.
Syntax		FREQUENCIES VARIABLES=Age DoA /FORMAT=NOTABLE /NTILES=4 /STATISTICS=STDDEV MINIMUM MAXIMUM MEAN MEDIAN SKEWNESS SESKEW KURTOSIS SEKURT /ORDER=ANALYSIS.
Resources	Processor Time	00:00:00.02
	Elapsed Time	00:00:00.02

Statistics

		Age	DoA
N	Valid	1473	1473
	Missing	0	0

Mean		56.46	8.70
Median		56.00	7.00
Std. Deviation		16.849	7.781
Skewness		.056	4.693
Std. Error of Skewness		.064	.064
Kurtosis		-.728	36.568
Std. Error of Kurtosis		.127	.127
Minimum		18	1
Maximum		97	101
Percentiles	25	44.00	5.00
	50	56.00	7.00
	75	69.00	10.00
